# Dairy Consumption and Risk of Cardiovascular and Bone Health Outcomes in Adults: An Umbrella Review and Updated Meta-Analyses

**DOI:** 10.3390/nu17172723

**Published:** 2025-08-22

**Authors:** Payam Sharifan, Roshanak Roustaee, Mojtaba Shafiee, Zoe L. Longworth, Pardis Keshavarz, Ian G. Davies, Richard J. Webb, Mohsen Mazidi, Hassan Vatanparast

**Affiliations:** 1 College of Pharmacy and Nutrition, University of Saskatchewan, Saskatoon, SK S7N 5E5, Canada; payam.sharifan@usask.ca (P.S.); mojtaba.shafiee@usask.ca (M.S.); zoe.longworth@usask.ca (Z.L.L.); pak526@mail.usask.ca (P.K.); 2 Department of Food and Nutrition Policy and Planning Research, National Nutrition and Food Technology Research Institute, Faculty of Nutrition Sciences and Food Technology, Shahid Beheshti University of Medical Sciences, Tehran 19816-19573, Iran; r_roustaee@yahoo.com; 3 School of Public Health, University of Saskatchewan, Saskatoon, SK S7N 5E5, Canada; 4 Margaret Ritchie School of Family and Consumer Sciences, College of Agricultural and Life Sciences, University of Idaho, Moscow, ID 83844, USA; 5 Research Institute of Sport and Exercise Sciences, Faculty of Science, Liverpool John Moores University, Liverpool L3 3AF, UK; i.g.davies@ljmu.ac.uk; 6 Nutrition and Food Science, School of Health and Sport Sciences, Liverpool Hope University, Liverpool L16 9JD, UK; webbr1@hope.ac.uk; 7 Clinical Trial Service Unit, Nuffield Department of Population Health, University of Oxford, Oxford OX3 7LF, UK; mazidi_ns@yahoo.com

**Keywords:** dairy consumption, cardiovascular disease, bone health, meta-analysis, umbrella review

## Abstract

Background/Objectives: The relationship between dairy consumption and cardiovascular or bone health outcomes remains controversial, with inconsistent findings across existing meta-analyses. In this study, we aimed to systematically evaluate and synthesize the evidence from published meta-analyses on dairy consumption and cardiovascular and bone health outcomes in adults, and to conduct updated meta-analyses incorporating recently published prospective cohort studies. Methods: We performed an umbrella review following PRISMA guidelines, searching published and grey literature up to April 2024. Meta-analyses evaluating dairy intake and its impact on cardiovascular and bone health outcomes were included. Updated meta-analyses were conducted for cardiovascular outcomes, while bone health outcomes were synthesized qualitatively. Methodological quality was assessed using the Joanna Briggs Institute checklist. Random-effects models were applied, and heterogeneity, small-study effects, excess significance, and prediction intervals were evaluated. Results: We included 33 meta-analyses (26 on cardiovascular, 7 on bone health outcomes). Updated meta-analyses showed that total dairy (RR: 0.96), milk (RR: 0.97), and yogurt (RR: 0.92) were significantly associated with reduced CVD risk. Total dairy and low-fat dairy were inversely linked to hypertension (RRs: 0.89, 0.87), and milk and low-fat dairy were associated with reduced stroke risk. Small-study effects were absent for most associations. Credibility was rated as “weak” for most associations, with total dairy and stroke, and total dairy and hypertension showing "suggestive" evidence. For bone health, dairy—especially milk—was linked to higher bone mineral density (BMD). Evidence on osteoporosis risk was mixed, and while total dairy and milk showed inconsistent associations with fractures, cheese and yogurt showed more consistent protective effects. Limited evidence suggested milk may reduce bone resorption markers. Conclusions: This review suggests that dairy consumption, particularly milk and yogurt, is modestly associated with reduced cardiovascular risk, while dairy intake appears to benefit BMD and fracture prevention. However, further research is needed to confirm these associations.

## 1. Introduction

Cardiovascular disease (CVD) is the leading cause of death worldwide, affecting more than half a billion people around the globe and accounting for over 20 million deaths in 2021, which is close to a third of all deaths globally [1]. CVDs are chronic diseases, and they encompass a wide range of conditions, including coronary heart disease (CHD) and stroke and peripheral blood vessels [2]. The etiology of CVD includes atherosclerosis, an inflammatory process that leads to hardening and narrowing of blood vessels, the buildup of fatty deposits in vessel walls, and eventually heart attack or stroke [3,4]. Osteoporosis is also a chronic disease that affects both men and women, particularly those over 50, with one in three women and one in five men in this age group worldwide expected to experience an osteoporotic fracture [5]. Osteoporosis involves loss of bone mass, weakening of bone structure, and reduced strength, making bones more fragile and prone to fractures [6,7]. Although the etiology of osteoporosis is still unknown, sex, age, genetic factors, and lifestyle play a role in its development [8].

Osteoporosis and CVD were long viewed as independent of each other [9]. However, numerous epidemiological studies have provided evidence of a link between CVD and osteoporosis [10,11]. This is due, in part, to shared conventional risk factors such as aging, smoking, physical inactivity, diabetes, alcohol consumption, and dietary factors, such as dairy intake [9,12,13]. However, a growing body of biological and epidemiological evidence indicates that there are common pathophysiological mechanisms underlying these diseases that are beyond shared risk factors [9,12,13]. For example, inflammation, oxidative stress, dyslipidemia, and hyperhomocystinuria are associated with both the atherosclerotic process and bone remodeling impairment and might explain, in part, the co-existence of osteoporosis and CVD [14].

Diet, as a modifiable risk factor, plays an important role in the development and management of both CVD and osteoporosis. Among dietary components, dairy products have received particular attention for their complex relationship with chronic diseases [15]. Dairy products are rich in high-quality protein (e.g., casein and whey), which supports muscle mass and may improve vascular function [16]; vitamins (e.g., riboflavin, vitamin D (via fortification), and vitamin B-12), which contribute to bone mineralization, neuromuscular function, and cardiovascular health [17,18]; and minerals such as calcium, potassium, and magnesium [19]. Calcium is a major structural component of bone and also plays a role in vascular tone, while potassium aids vasodilation and sodium balance [20], and magnesium supports endothelial function and glucose metabolism [21]. The saturated fat content in dairy products, on the other hand, may adversely affect cardiovascular outcomes like CHD [22,23]. Several meta-analyses have investigated the relationship between dairy consumption and various cardiovascular outcomes, yet the findings were inconsistent [24,25,26,27,28]. Some studies, like Chen et al. (2022), found total dairy intake was linked to lower risks of hypertension, CHD, and stroke, though results varied by dairy type [24]. Others, such as Jakobsen et al. (2021) [25] and Naghshi et al. (2022) [26], reported mixed effects depending on the type and fat content of dairy. While some showed protective associations for cheese or total dairy [25], others noted increased risks with high-fat milk [26]. Similarly, Mazidi et al. (2019) found inverse associations for total dairy and cheese intake with all-cause and cerebrovascular mortality but also reported increased CHD mortality with milk consumption [27]. In contrast, Bechthold et al. (2019) found no significant associations between dairy intake and CHD or stroke risk [28]. These varied and sometimes contradictory results highlight the importance of an umbrella review that systematically synthesizes existing evidence and updates it with newly available data.

In terms of osteoporosis, current evidence suggests that a good nutritional status and adequate intake of protein, calcium, and vitamin D have a positive influence on bone health [29]. Dairy products, being rich sources of these key nutrients, are therefore considered important dietary components for maintaining bone integrity and reducing osteoporosis risk [30]. We have previously shown that milk and its alternatives supply almost 40% of dietary calcium and 35% of dietary vitamin D in Canadians [31,32]. While numerous meta-analyses have examined the relationship between dairy intake and bone health outcomes, their findings remain mixed and, at times, contradictory [33,34,35]. Hidayat et al. (2022) found milk supplementation improved bone mineral density (BMD) at the hip and spine, but not at the femoral neck or whole body [33]. Shi et al. (2020) reported positive associations between dairy and bone mineral density at multiple sites [34]. Malmir et al. (2020) saw a protective link between total dairy intake and osteoporosis risk, but not with milk alone [35]. These inconsistencies across meta-analyses highlight the need for a comprehensive umbrella review to clarify the role of dairy products in the prevention of osteoporosis and related bone health outcomes.

Given the inconsistencies in existing meta-analyses regarding the role of dairy consumption on cardiovascular and bone health outcomes, a comprehensive synthesis of the evidence is warranted. Moreover, recent original studies not included in previous meta-analyses may provide additional insights and help clarify these associations. Therefore, the aim of this umbrella review is to systematically evaluate and summarize the available meta-analyses on dairy intake in relation to cardiovascular disease and bone health outcomes and, where appropriate, to conduct updated meta-analyses incorporating newly published prospective studies.

## 2. Materials and Methods

The protocol for this umbrella review was registered in Open Science Framework (DOI: 10.17605/OSF.IO/J792H). Our umbrella review adopted the Preferred Reporting Items for Systematic Reviews and Meta-Analyses (PRISMA) guidelines [36]. We systematically searched for white and gray literature from inception until April 2022 to evaluate the impact of dairy product consumption, including total dairy, milk, yogurt, cheese, and butter, on various cardiovascular and bone health outcomes. Databases searched included MEDLINE and In-Process, In-Data-Review & Other Non-Indexed Citations via Ovid, EMBASE via Ovid, Cochrane Database for Systematic Reviews via Ovid, CINAHL via EBSCO, Web of Science (Current Contents Connect, CAB Abstracts, Data Citation Index, Derwent Innovation Index, FSTA—the food science resource, KCI Korean Journal Database, MEDLINE, Russian Science Citation Index, SciELO Citation Index, Zoological Record) via Clarivate, LILACS via the Virtual Health Library, and the systematic review registry PROSPERO. Additionally, we searched gray literature sources from relevant organizations, including Dairy Farmers of Canada, the International Osteoporosis Foundation, and the American Heart Association. In April 2024, the literature search was updated in MEDLINE and In-Process, In-Data-Review & Other Non-Indexed Citations via Ovid. Keywords and MeSH descriptors are summarized in Supplementary Table S1.

### 2.1. Inclusion and Exclusion Criteria

Studies that met the following criteria were included in our umbrella review: (i) quantitative systematic reviews and meta-analyses of empirical research quality; (ii) adult population (19 years and older); (iii) the exposure or intervention under study is the intake of dairy products; (iv) outcomes under study included a range of cardiovascular health outcomes (e.g., total CVD, CHD, stroke, and hypertension) and bone health-related outcomes (e.g., BMD, osteoporosis risk, fracture risk, and bone turnover biomarkers). There were no restrictions on sex or demographic characteristics nor on the comparison/control group. We excluded congenital cardiovascular outcomes, viral or bacterial infection cardiovascular outcomes, or trauma-induced cardiovascular outcomes. We also excluded any study reporting dairy products fortified with probiotics.

### 2.2. Study Screening and Critical Appraisal

All retrieved studies were imported into EndNote, and duplicate records were removed using the de-duplication method. The remaining studies were independently screened in three stages by two reviewers (ZL, EK) using Covidence systematic review software. First, titles and abstracts were screened against the inclusion/exclusion criteria. When the reviewers were unable to determine the eligibility of a study by the title and abstract alone, the study progressed to the next stage. Second, once studies passed the initial screening, the two reviewers (ZL, EK) independently critically appraised the studies based on their methodological quality using the Joanna Briggs Institute (JBI) critical appraisal checklist for systematic reviews and research syntheses (Supplementary Table S2). A study was deemed high quality if the two reviewers agreed to select ‘yes’ on the JBI critical appraisal checklist ten or more times, moderate quality if ‘yes’ was selected seven to nine times, and low quality if ‘yes’ was selected less than seven times. Studies were excluded from the umbrella review if the reviewers deemed the study low quality. Finally, studies that were deemed empirical research quality progressed to full-pass review, where the two reviewers (ZL, EK) independently screened the studies against the inclusion/exclusion criteria. When disagreements arose during study selection, either at the title and abstract stage or the full text screening stage, or during the critical appraisal, a third reviewer (MS) independently assessed the eligibility of the study to reach a consensus. Inter-rater reliability between reviewers (ZL, EK) in both stage 1 (title and abstract screening) and stage 3 (full-pass review) was assessed using Cohen’s Kappa statistic and computed using Covidence software (https://www.covidence.org/).

### 2.3. Identification of New Original Studies for Updated Meta-Analyses

To perform an updated meta-analysis, we focused on exposure–outcome pairs for which at least five new original studies meeting our inclusion criteria had been published since the most recent study included in the previous meta-analysis. For this purpose, two reviewers (PS and RR) conducted a systematic hand search in PubMed and independently screened recent original articles. The screening process involved three stages for each exposure-outcome: title/abstract review, full-text review, and critical appraisal. Critical appraisal was performed using the Newcastle–Ottawa Scale (NOS) [37]. Based on the NOS scores, studies were classified as high quality (scores of 7 to 9), medium quality (scores of 4 to 6), or low quality (scores below 4) (Supplementary Table S3). Data extracted from the studies, including title, author names, year of publication, study population, exposure(s), outcome(s), Relative Risks (RR) or Hazard Ratios (HR), 95% confidence intervals (CI), number of cases, sample size, and NOS scores, were entered into Excel spreadsheets. Any discrepancies between the two Excel files were resolved through joint re-evaluation of the data by both reviewers (PS and RR). The characteristics and the description of the original studies included in the updated meta-analyses are presented in Supplementary Tables S4 and S5. Notably, cheese was not included in our analysis, as a recently published umbrella review and updated meta-analysis of prospective studies has already comprehensively addressed cheese consumption in relation to multiple health outcomes, including CVD [38].

### 2.4. Data Extraction and Synthesis

Data extraction was conducted in two stages. Initially, two independent investigators (ZL and PK) extracted general information from the included meta-analyses. This included information such as study type, search strategy, objectives, exposures and outcomes assessed, study population, and appraisal instruments (Supplementary Table S6). Subsequently, a more detailed data extraction was performed by two other independent investigators (PS and RR), who collected comprehensive information from each meta-analysis into a structured spreadsheet. This included the first author’s name, publication year, number of included primary studies, number of participants, number of cases, exposure type, exposure definition, comparison, outcome, outcome definition, model of synthesis, model of weight, heterogeneity statistics (*I*^2^, Q-test *p*-value), methods for assessing publication bias (e.g., funnel plot, Egger’s test, Begg’s test) and their results, as well as sensitivity analysis methods (e.g., leave-one-out analysis) and corresponding findings. Any discrepancies during either stage were resolved through discussion.

The second spreadsheet contained data from the primary studies included in each meta-analysis. This included the first author’s name, publication year, number of participants, number of cases, type of effect size measurement (RR, HR, OR), effect estimates, and 95% confidence intervals. To synthesize the data, each meta-analysis was reanalyzed by pooling the primary studies that were included in the original meta-analyses. Effect sizes and their corresponding confidence intervals were extracted from forest plots of each meta-analysis, and a new meta-analysis was conducted for each exposure-outcome pair after removing duplicates. When two or more similar primary studies were found, we selected data derived from the most recent meta-analyses.

### 2.5. Data Analysis

All statistical analyses were performed using R software (version 2024.04.2+764). For each meta-analysis, we estimated the summary effect size and its 95% confidence intervals (CI) using both fixed-effect and random-effects models to reflect different assumptions about study variability. In cases where necessary, we converted effect sizes to ensure consistency across studies, assuming hazard ratios (HR) to be equivalent to relative risks (RR) when analyzing incidence outcomes [39]. Additionally, we estimated the 95% prediction interval (PI) to account for between-study heterogeneity and assess the uncertainty in the expected effect in future studies. To quantify between-study heterogeneity, we used the *I*^2^ statistic, which ranges from 0% to 100%. Values between 0% and 40% might not be important, 30% to 60% may indicate moderate heterogeneity, 50% to 90% substantial heterogeneity, and 75% to 100% considerable heterogeneity [40]. The statistical power of each meta-analysis was assessed by calculating the power of the largest primary study (based on sample size, typically with the smallest standard error [SE]), using its effect size and SE (<0.10 as a precision criterion).

To assess potential small-study effects, where smaller studies might show greater effect sizes compared to larger studies, we applied Egger’s regression asymmetry test, considering a *p*-value of less than 0.10 as indicative of potential publication bias. We also evaluated excess significance bias, potentially arising from publication bias or selective reporting, using a chi-square test to compare the observed number of studies with nominally significant results (*p* < 0.05) against the expected number. The expected count was calculated based on the effect size of the largest study (with the smallest standard error) using a noncentral t-distribution. Excess statistical significance was indicated if the two-sided *p*-value was less than 0.10 and the observed significant studies exceeded the expected number.

### 2.6. Sensitivity Analysis and Small Study Error Correction

If publication bias was detected, we applied statistical adjustments to correct for its potential impact on meta-analytic results. Specifically, we used the trim-and-fill method, which estimates the number of missing studies due to publication bias and imputes their effect sizes to restore funnel plot symmetry, thereby providing an adjusted summary effect size. Additionally, we employed the Copas selection model, which accounts for the probability of study publication based on effect size and standard error, offering a sensitivity analysis to assess the robustness of findings under varying levels of selection bias.

### 2.7. Assessment of Credibility

The credibility of the associations reported in these meta-analyses was assessed using a combination of criteria, including sample size, number of cases, heterogeneity, prediction intervals, small-study effects (Egger’s test), and excess significance bias, in accordance with previous umbrella reviews [41,42]. Associations were graded into five levels of credibility based on strength and quality of evidence. *Convincing* (Class I) evidence required ≥1000 cases, *p* < 10^−6^, low heterogeneity (*I*^2^ < 50%), a 95% prediction interval excluding the null, no bias, and a significant largest study. *Highly suggestive* (Class II) associations met most of these criteria, including a significant largest study and *p* < 10^−6^. *Suggestive* (Class III) evidence required ≥1000 cases and *p* < 0.001. *Weak* (Class IV) evidence included associations with *p* < 0.05 but without stronger supporting criteria. Associations with *p* ≥ 0.05 were considered *non-significant* (Class V), indicating no reliable evidence of an effect.

## 3. Results and Discussion

**Figure 1:**
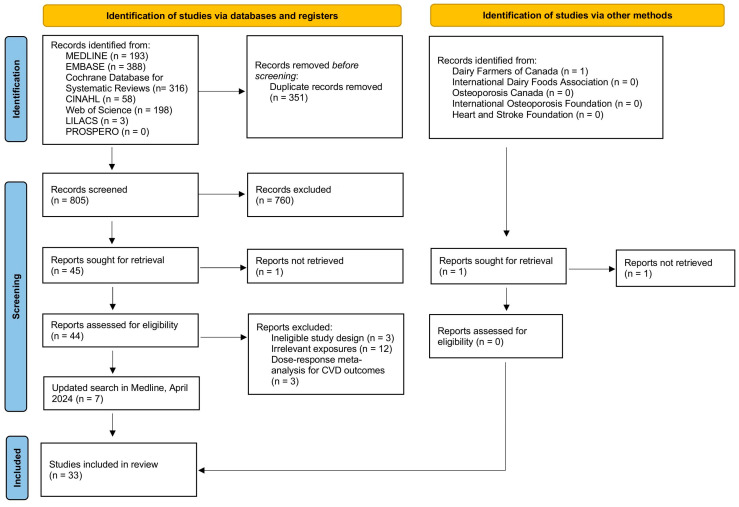
Flow diagram of the study search and selection process.

Figure 1 illustrates the flowchart of the study identification and selection process. In the present study, a total of 1156 records were identified through database searches. After removing 351 duplicates, 805 records remained for screening. Of these, 760 were excluded based on title and abstract review, leaving 45 reports sought for retrieval. Among these, one report could not be retrieved, and 18 were excluded for reasons such as ineligible study design (*n* = 3); focus on irrelevant exposures (*n* = 12); and use of dose–response meta-analysis (*n* = 3) (Supplementary Table S7). An April 2024 MEDLINE update added seven more studies, leading to the final inclusion of 33 meta-analyses (26 on cardiovascular events, 7 on bone health outcomes). A manual PubMed search also identified seven meta-analyses (Supplementary Tables S4 and S5), which were added to the re-analysis.

**Methodological quality:** The evaluation of methodological quality for previous meta-analyses using the JBI checklist is summarized in Supplementary Table S8. A total of 13 meta-analyses [24,26,27,34,35,43,44,45,46,47,48,49,50] were rated as high quality, 15 meta-analyses [25,28,33,51,52,53,54,55,56,57,58,59,60,61,62,63] as moderate quality, and the remaining four meta-analyses [64,65,66,67] as low quality, which were excluded from further analysis.

### 3.1. Cardiovascular Outcomes

**Figure 2:**
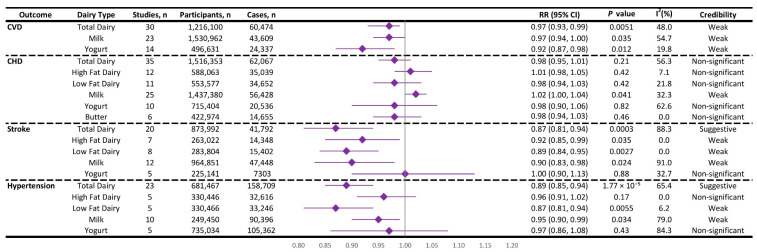
Association between dairy consumption (highest compared with lowest intake level) and CVD outcomes based on updated meta-analyses. RR: Relative Risk.

In this umbrella review and updated meta-analysis, we evaluated the association between dairy consumption—including total dairy, milk, yogurt, and butter—and CVD outcomes—including total CVD, CHD, stroke, and hypertension—through 23 meta-analyses (Supplementary Table S9). These covered 15 meta-analyses on total CVD [26,44,46,47,48,51,52,53,54,56,59,60,61,62,63], 13 on CHD [24,25,27,28,44,47,48,51,53,54,57,59,62], 11 on stroke [24,25,28,47,48,51,53,54,57,59,62], and 4 on hypertension [24,43,55,58]. Regarding dairy subtypes, 15 meta-analyses assessed total dairy [24,25,26,27,28,43,44,48,51,52,54,55,56,58,59], 11 assessed milk [24,25,26,27,43,44,51,55,57,58,59], 13 assessed yogurt [24,25,43,44,46,48,51,55,58,60,61,62,63], 10 assessed cheese [24,25,43,44,48,51,53,55,58,63], and 3 assessed butter [25,47,48]. In addition, high-fat and low-fat dairy subgroups were analyzed in 11 meta-analyses [24,25,26,27,28,43,44,48,55,58,59]. All primary studies from these meta-analyses were extracted and re-analyzed using a highest versus lowest category comparison approach, specifically for cardiovascular outcomes. This re-analysis yielded 19 unique associations (Figure 2, Supplementary Table S10), which will be presented and discussed below.

#### 3.1.1. Cardiovascular Disease (CVD) Risk

***Total Dairy Consumption****:* A total of 30 prospective cohort studies examining total dairy consumption and CVD risk were re-analyzed, including 1,216,100 participants and 60,474 cases. The highest-versus-lowest consumption comparison yielded a random effect pooled RR of 0.96 (95% CI: 0.93, 0.99, *p* = 0.005), suggesting a *weak* inverse association with moderate heterogeneity (*I*^2^ = 48%, Figure 2, Supplemental Figure S1). However, our findings revealed a 95% prediction interval (PI: 0.87–1.06), indicating uncertainty in the effect size for future studies (Supplementary Table S10). The absence of small-study effects or excess statistical significance suggests minimal publication bias, enhancing data reliability. However, the largest study’s near-perfect power (99.99%) yet non-significant result highlights the association’s fragility, contributing to its weak credibility. Given the weak credibility, the small effect size likely holds limited clinical significance.

Our findings support the hypothesis that dairy consumption may be associated with a small reduction in CVD risk. This result is consistent with several previous meta-analyses [26,48,54,56]. Naghshi et al. (2022) [26], Mishali et al. (2019) [56], Gholami et al. (2017) [54], and Qin et al. (2015) [48] found inverse associations, with some indicating up to a 10% risk reduction and relatively low to moderate heterogeneity. These findings suggest a possible beneficial effect of dairy intake, although Naghshi et al. also noted that the relationship may not be strictly linear [26]. In contrast, other studies such as Guo et al. (2017) [44] and Alexander et al. (2016) [51] found weaker or non-significant associations, with wider confidence intervals and moderate heterogeneity, indicating less certainty about the protective effect. Bhandari et al. (2023) found no significant association with cardiovascular mortality and reported very high heterogeneity, which indicates substantial inconsistency among the included studies [52]. This discrepancy may arise from a very small true effect size, methodological differences (e.g., varying adjustments for confounders), or heterogeneity in dairy types (e.g., fermented vs. high-fat) and dietary patterns across populations, which likely dilute the effect. The collective evidence—including our findings—suggests a weak inverse association between total dairy intake and CVD risk. This potential benefit may be mediated by various components in dairy products, such as calcium, potassium, bioactive peptides, and probiotics, which may exert favorable effects on blood pressure [68,69,70,71], lipid metabolism [72,73], and vascular function [74,75,76,77]. However, unlike previous reviews, our umbrella analysis incorporated a broader evaluation of study quality, credibility, and bias. Notably, while earlier studies reported statistically significant associations, our analysis revealed that the 95% prediction interval included the null, suggesting that future studies might yield inconsistent findings.

***Milk Consumption****:* In our updated meta-analysis of 23 prospective cohort studies involving 1,530,962 participants and 43,609 cases, higher milk consumption was associated with a modest, statistically significant reduction in CVD risk. The random effect pooled RR for the highest versus lowest intake category was 0.969 (95% CI: 0.941, 0.998, *p* = 0.035), suggesting a small but statistically significant protective effect with moderate heterogeneity (*I*^2^ = 54.7%, Figure 2, Supplemental Figure S2). This is consistent with Soedamah-Muthu et al. (2011) [59], who reported a modest inverse association with no heterogeneity, and Alexander et al. (2016) [51], who found a non-significant but directionally similar result. However, other large-scale meta-analyses, including those by Naghshi et al. (2022) [26] and Guo et al. (2017) [44], reported null associations, with Naghshi et al. also noting high heterogeneity in both categorical and dose–response analyses. These discrepancies may be attributed to differences in the definition of milk intake (e.g., total milk vs. specific types such as whole or skim milk), outcome (CVD incidence vs. mortality), serving sizes, and geographic differences in milk consumption patterns. The beneficial components of milk, such as calcium, potassium, magnesium, and bioactive peptides such as lactotripeptides (LTPs), may decrease the risk of hypertension [68,69,78,79] and improve vascular health [74,75,76,80], yet the saturated fat content in certain milk types may counteract these benefits in some populations [81,82].

While the unadjusted results indicate a small protective association between milk consumption and CVD risk, this effect is undermined by several methodological concerns. The 95% prediction interval included the null (PI: 0.892–1.054), suggesting uncertainty in replicability (Supplementary Table S10). The largest study had low statistical power (15.8%), and Egger’s test revealed small-study effects (*p* < 0.10), pointing to potential publication bias. Adjustments using the trim-and-fill method and the Copas selection model both attenuated the association, rendering it statistically non-significant (Supplementary Table S11). No excess statistical significance was found, but the evidence was classified as *weak* based on established criteria [41]. These findings suggest that the observed protective effect is likely overstated and not robust, highlighting the need for more rigorous, high-powered studies to clarify the relationship between milk intake and CVD risk.

***Yogurt Consumption****:* Our updated meta-analysis of 14 prospective cohort studies, including 496,631 participants and 24,337 cases, demonstrated a statistically significant inverse association between higher yogurt consumption and CVD risk with a random effect pooled RR of 0.92 (95% CI: 0.87, 0.98, *p* = 0.012) and low heterogeneity (*I*^2^ = 19.8%, Figure 2, Supplemental Figure S3). These findings suggest that yogurt consumption may offer cardiovascular benefits, aligning with several recent meta-analyses [46,60,61,63]. Kazemi et al. (2023) reported an 8% reduction in CVD mortality and a 14% reduction per 200 g/day increase in yogurt intake [46]. Similar protective effects were observed by Tutunchi et al. (2023) [61] and Sun et al. (2023) [60], the latter noting a significant reduction in CVD mortality. In contrast, earlier meta-analyses by Guo et al. (2017) [44], Wu et al. (2017) [62], and Alexander et al. (2016) [51] found non-significant or mixed results, likely due to fewer included studies or smaller sample sizes, potentially limiting statistical power. Overall, more recent evidence supports a beneficial role of yogurt in reducing CVD risk. The relatively consistent and favorable findings for yogurt may be attributable to its unique nutritional and microbial profile. Yogurt is a fermented dairy product that not only provides calcium, potassium, vitamin D (through fortification), and high-quality protein [83,84], but also delivers live probiotic cultures that may influence cardiometabolic risk factors, such as lipid profiles, blood pressure, and systemic inflammation [85,86,87]. These potential mechanisms may explain the observed protective effect and suggest that yogurt may offer cardiovascular benefits beyond those of non-fermented milk or total dairy intake.

Unlike previous reviews, our umbrella review incorporated a broader evaluation framework, which provides a more rigorous assessment of the evidence base. Despite the observed protective association, the 95% prediction interval (PI: 0.80–1.07) includes the null, indicating uncertainty about the effect’s consistency in future studies (Supplementary Table S10). The lack of small-study effects and excess significance suggests minimal publication bias, which supports the reliability of the data. However, the largest study had low statistical power (34%) and a non-significant result, and the association was classified as having *weak* credibility. These factors—along with the modest effect size and methodological limitations—warrant cautious interpretation. Given the weak credibility and small effect size, the clinical significance of the association is likely limited.

#### 3.1.2. Coronary Heart Disease (CHD) Risk

***Total Dairy Consumption****:* A re-analysis of 35 primary studies assessing total dairy consumption and CHD risk, including 1,516,353 participants and 62,067 cases, found no significant association between total dairy consumption and CHD risk (RR = 0.98, 95% CI: 0.95–1.01, *p* = 0.21, *I*^2^ = 56.3%; Figure 2, Supplemental Figure S4). For high-fat dairy, 12 studies involving 588,063 participants and 35,039 cases showed no significant association with CHD risk, with a pooled RR of 1.01 (95% CI: 0.98, 1.05, *p* = 0.42, *I*^2^ = 7.1%, Figure 2, Supplemental Figure S5). Similarly, for low-fat dairy consumption, 11 studies with 553,577 participants and 34,652 cases yielded a pooled, non-significant RR of 0.98 (95% CI: 0.94, 1.03, *p* = 0.42, *I*^2^ = 21.8%, Figure 2, Supplemental Figure S6). No small-study effects or excess statistical significance were observed across all analyses, supporting the robustness of these null findings (Supplementary Table S10).

Our findings align with most prior meta-analyses, which reported no significant association between total dairy consumption and CHD [24,25,27,28,44,48,54]. Studies by Chen et al. (2022) [24], Jakobsen et al. (2021) [25], Mazidi et al. (2019) [27], Bechthold et al. (2019) [28], Gholami et al. (2017) [54], Guo et al. (2017) [44], and Qin et al. (2015) [48] consistently found null associations across total, high-fat, and low-fat dairy intake. These results suggest no differential CHD risk based on dairy fat content. Although Alexander et al. (2016) reported a possible protective effect at higher intake levels, these findings were based on fewer studies and may reflect population-specific or nonlinear associations [51]. Overall, the evidence indicates that dairy intake, regardless of fat content, does not significantly influence CHD risk. These findings are important in light of ongoing debates about the role of saturated fat in dairy products and cardiovascular health [88,89]. The lack of observed harm may be partly due to the complex nutritional composition of dairy, which includes potentially cardioprotective components such as calcium, potassium, magnesium, and bioactive peptides.

***Milk Consumption****:* Milk consumption, based on 25 primary studies with 1,437,380 participants and 56,428 cases, showed a slight but statistically significant increase in CHD risk, with a relative risk of 1.0189 (95% CI: 1.0007, 1.0374, *p* = 0.041), with low heterogeneity (*I*^2^ = 32.3%) (Figure 2, Supplemental Figure S7). This finding is in contrast with most previous meta-analyses, which largely reported no association [24,25,44,57,59]. In contrast, Mazidi et al. (2019) reported a significant positive association between milk intake and CHD mortality (RR = 1.04, 95% CI: 1.02, 1.06), though their analysis was based on only three studies [27]. While this study may support our results, differences in outcome definitions (CHD mortality vs. incidence) and study inclusion criteria should be considered. Despite statistical significance, several factors necessitate a cautious interpretation of our finding. First, a very small effect size (a ~2% increase in relative risk), suggesting limited clinical relevance. Second, the finding lacks robustness, as evidenced by a 95% confidence interval that barely excludes the null value of 1.0. This proximity to the null implies that the conclusion is fragile and sensitive to the inclusion of future data. Furthermore, the 95% predictive interval (PI: 0.979–1.061), which forecasts the range of potential outcomes in future studies, broadly encompasses the null (Supplementary Table S10). This indicates that the observed association may not be consistently reproducible, and a future study could plausibly report a null or even a slightly protective effect. While tests for small-study effects (Egger test) did not suggest the presence of publication bias, a test for excess statistical significance was positive. This latter finding indicates that the number of “significant” results among the included studies is higher than would be expected by chance, which may point to selective reporting or other methodological biases in the literature.

One possible explanation for the slight increase in CHD risk is the saturated fat content in milk, particularly palmitic acid, which has been shown to raise LDL cholesterol levels [90,91]—a well-established risk factor for CHD. Supporting this, a prospective cohort study of women in the Nurses’ Health Study found that whole-milk consumption was associated with a significantly increased risk of CHD. In contrast, greater consumption of skim milk has been associated with a lower risk of CHD, but this did not reach statistical significance [23]. However, as mentioned earlier, milk contains beneficial components that may contribute to cardiovascular improvements. These include calcium, which may lower blood pressure [92]; potassium, which supports vascular health [75]; and bioactive peptides that may have anti-hypertensive, anti-inflammatory, or lipid-lowering effects [79,80]. These protective elements could mitigate some of the harmful effects of saturated fat, which may potentially explain the modest effect size observed in the current analysis. Considering these mixed nutritional effects, there is a need for subgroup analyses to determine whether the association with CHD risk differs between low-fat and high-fat milk.

***Yogurt Consumption*:** In our updated meta-analysis of 11 studies with 715,404 participants and 20,536 cases, yogurt consumption was not associated with CHD risk (RR = 0.98, 95% CI: 0.91, 1.07, *p* = 0.82), with substantial heterogeneity (*I*^2^ = 62.6%) (Figure 2, Supplemental Figure S8). This null association is well-supported by several key indicators. We found no evidence of small-study effects or excess statistical significance, suggesting the literature is not skewed by publication bias or selective reporting. Additionally, the 95% predictive interval was wide and centered on the null (PI: 0.83–1.16), indicating that a clinically meaningful effect is unlikely to be found in future studies (Supplementary Table S10). This conclusion is further reinforced by the fact that the largest study in our analysis also reported a clear null result (*p* = 0.58). This finding is consistent with multiple prior meta-analyses [24,25,44,48,51,62]. Chen et al. (2022) reported no association in both high vs. low and dose–response analyses [24]. Jakobsen et al. (2021) similarly found no association with CHD comparing the highest with the lowest category of yogurt intake [25]. Earlier meta-analyses by Qin et al. (2015) [48], Alexander et al. (2016) [51], Wu et al. (2017) [62], and Guo et al. (2017) [44] also failed to detect a significant relationship between yogurt consumption and CHD risk, which shows the stability of this finding across time and methodologies. Despite yogurt’s nutritional profile, its intake does not appear to significantly influence CHD risk based on current epidemiologic evidence. However, population-level variability in yogurt types (e.g., plain vs. sweetened, full-fat vs. low-fat) and consumption patterns may dilute potential effects.

***Butter Consumption*:** In our updated meta-analysis of 6 studies with 422,974 participants and 14,655 cases, we observed no significant association between butter consumption and CHD risk (RR = 0.99, 95% CI: 0.94, 1.03, *p* = 0.46), with no observed heterogeneity across studies (*I*^2^ = 0%) (Figure 2, Supplemental Figure S9). The narrow 95% predictive interval (PI: 0.94–1.04) suggests that future studies are also likely to find no meaningful effect (Supplementary Table S10). Additionally, no evidence of small-study effects was detected, and the largest study in our analysis reported a clear null result (*p* = 0.74). Together, these findings provide strong support for a stable and robust null association.

Our findings align with findings from other recent systematic reviews and meta-analyses [25,47,48]. Jakobsen et al. (2021) found no association between butter intake and CHD in both high vs. low and dose–response analyses [25]. Qin et al. (2015) [48] and Pimpin et al. (2016) [47] similarly showed no significant association between butter intake and CHD. The lack of a link between butter intake and CHD risk might seem surprising given butter’s relatively high saturated fat content, which has historically been linked to increased cardiovascular risk [93]. However, butter contains not only saturated fats but also short-chain fatty acids (SCFAs), which may help regulate metabolic diseases by influencing glucose metabolism, lipid accumulation, and fat oxidation [94]. Although our findings indicate that butter consumption is not significantly associated with CHD risk, the relatively small number of studies limits our ability to conduct meaningful subgroup or dose–response analyses.

#### 3.1.3. Total Stroke Risk

***Dairy Consumption*:** In our updated meta-analysis of 20 prospective cohort studies including 873,992 participants and 41,792 cases, we found that total dairy consumption was significantly associated with a reduced risk of stroke (RR = 0.87, 95% CI: 0.81, 0.94, *p* = 0.0003), although heterogeneity was considerable (*I*^2^ = 88.2%) (Figure 2, Supplemental Figure S10). The 95% prediction interval (PI: 0.65–1.17) included the null, indicating uncertainty in the effect’s reproducibility across future studies (Supplementary Table S10). No evidence of small-study effects was detected (Egger’s test *p* = 0.49), and no excess statistical significance was observed. The association was rated as *suggestive* in credibility, supported by the high power (99%) and statistical significance of the largest contributing study. Subgroup analyses further showed inverse associations for both high-fat and low-fat dairy. High-fat dairy consumption was associated with reduced stroke risk (RR = 0.92, 95% CI: 0.85–0.99, *p* = 0.035; Figure 2, Supplemental Figure S11), though the 95% PI (0.84–0.99) was narrow and close to the null. The largest study in this subgroup was underpowered (power = 65%) and non-significant, and the association was classified as having *weak* credibility due to limited robustness. Similarly, low-fat dairy intake was inversely associated with stroke risk (RR = 0.89, 95% CI: 0.84–0.95, *p* = 0.0027; Figure 2, Supplemental Figure S12), with a 95% prediction interval of 0.83–0.96. While the largest study in this group was statistically significant and had moderate power (79%), the overall evidence was again deemed *weak* in credibility, and no small-study effects or excess significance were detected for either dairy subtype.

Our findings support previous meta-analyses reporting an inverse association between total dairy intake and stroke risk [24,48,51,54]. Studies by Chen et al. (2022) [24], Alexander et al. (2016) [51], Qin et al. (2015) [48], and Gholami et al. (2017) [54] consistently observed protective effects, including for both high-fat and low-fat dairy subtypes. These findings are in line with our own results showing reduced stroke risk across dairy types. However, not all studies agree. Alexander et al. (2016) [51] and Bechthold et al. (2019) [28] reported null or inconsistent associations, with the latter noting a modest 5% risk reduction for stroke with dairy intake up to ~500 g/day, but no significant differences by fat content. These findings collectively suggest that both dairy types may offer cardiovascular benefits, despite the concerns about the saturated fat content in high-fat dairy products. This protective effect against stroke may, at least in part, be attributed to the blood pressure–lowering properties of dairy products, as hypertension remains the most prevalent risk factor for stroke [95].

***Milk Consumption:*** In our meta-analysis of 12 prospective cohort studies with 964,851 participants and 47,448 cases, milk consumption was significantly associated with a reduced risk of stroke (RR = 0.90, 95% CI: 0.83, 0.98, *p* = 0.024). However, heterogeneity across studies was considerable (*I*^2^ = 91%), which indicates variability in study designs, populations, and exposure assessments (Figure 2, Supplemental Figure S13). Our findings are partly consistent with those of Jakobsen et al. (2021), who found a significant effect in high vs. low comparisons but not in dose–response [25]. The results of our analysis contrast with those of several other meta-analyses that have reported non-significant associations between milk intake and stroke risk [24,51,57,59]. The discrepancies between our findings and those of earlier meta-analyses may be attributed to differences in inclusion criteria and updated study pools.

Despite the observed significant association, the 95% prediction interval (0.67–1.22) included the null, indicating uncertainty about the association’s consistency in future studies (Supplementary Table S10). Egger’s test suggested the presence of small-study effects (*p* = 0.07), which may indicate potential publication bias or the influence of smaller studies with stronger effects. However, no evidence of excess statistical significance was observed. The largest study was statistically significant and well-powered (power = 99%), lending some support to the pooled estimate. Sensitivity analyses provided mixed results: the Trim and Fill method, which imputed six potentially missing studies, yielded a null association (RR = 1.00; 95% CI: 0.91–1.11; *p* = 0.90), suggesting that the observed effect may be influenced by publication bias (Supplementary Table S11). In contrast, the Copas selection model did not add any studies and produced a slightly attenuated but still significant result (RR = 0.90; 95% CI: 0.83–0.98) (Supplementary Table S11), reinforcing the possibility of a modest protective effect. Nevertheless, due to the presence of small-study effects, high heterogeneity, and inconsistent sensitivity analyses, the overall credibility of the association was rated as *weak*. These findings suggest that while milk intake may be associated with a reduced risk of stroke, the evidence remains uncertain and should be interpreted with caution.

***Yogurt Consumption:*** In our updated meta-analysis of five prospective cohort studies with 225,141 participants and 7303 cases, yogurt consumption was not significantly associated with stroke risk (RR = 1.00, 95% CI: 0.90, 1.13, *p* = 0.88), and relatively low heterogeneity (*I*^2^ = 32.7%) suggests moderate consistency among the included studies (Figure 2, Supplemental Figure S14). The 95% prediction interval (0.81–1.24) spanned the null, indicating substantial uncertainty in the association’s replicability in future research (Supplementary Table S10). No small-study effects were assessed (Egger’s test not applicable), and no excess statistical significance was detected. While the largest study was highly powered (power = 99%), it did not yield a statistically significant result. Based on these factors, the overall credibility of the association was classified as *non-significant*, indicating that current evidence does not support a meaningful relationship between yogurt intake and stroke risk.

Our results align with those of previous meta-analyses that have also found no significant association between yogurt intake and stroke [25,28,48,62]. Chen et al. (2022) reported a non-significant risk estimate in both high vs. low comparisons and dose–response analyses [24]. Similarly, Jakobsen et al. (2021), using data from three prospective studies, found no evidence of an association between yogurt consumption and ischemic stroke risk in either high vs. low intake or dose–response analyses [25]. Some older meta-analyses like Qin et al. (2015) and Wu et al. (2017) also supported the null association [48,62]. The consistency of null results across both our findings and those from previous meta-analyses suggests that yogurt may not play a significant role in the etiology of stroke. However, limited numbers of included studies and varying levels of yogurt intake across populations highlight the need for further well-designed prospective studies to clarify whether specific subtypes of yogurt could influence cerebrovascular outcomes.

#### 3.1.4. Hypertension

***Total Dairy Consumption:*** Our updated meta-analysis involving 23 primary studies, including 681,467 participants and 158,709 cases, demonstrated that total dairy consumption is significantly associated with a reduced risk of hypertension (RR = 0.89, 95% CI: 0.85, 0.94, *p* = 0.000017). However, there was substantial heterogeneity (*I*^2^ = 65.4%) among the included studies (Figure 2, Supplemental Figure S15). When dairy was stratified by fat content, low-fat dairy, analyzed in six studies with 330,466 participants and 33,246 cases, demonstrated a protective association against hypertension, with a relative risk of 0.87 (95% CI: 0.81, 0.94, *p* = 0.005) and low heterogeneity (*I*^2^ = 6.2%) (Figure 2, Supplemental Figure S16). In contrast, high-fat dairy consumption, based on 6 studies with 330,446 participants and 32,616 cases, was not significantly associated with hypertension risk (RR = 0.96, 95% CI: 0.91, 1.02, *p* = 0.17) (Figure 2, Supplemental Figure S17).

Our findings are in line with those from previous meta-analyses [24,43,55,58]. Chen et al. (2022) reported similar associations, showing that higher total and low-fat dairy intake were linked to a reduced risk of hypertension [24]. In contrast, high-fat dairy intake showed no association with hypertension risk, aligning with the current results. Their dose–response analyses further supported these findings, with each additional serving per day of total or low-fat dairy associated with reduced hypertension risk, while high-fat dairy remained non-significant [24]. These findings are consistent with those of Heidari et al. (2021) [55], Feng et al. (2022) [43], and Ralston et al. (2012) [58], all of whom reported inverse associations for total and low-fat dairy, but no association for high-fat dairy. Overall, the consistent findings across multiple meta-analyses support a protective role of total and particularly low-fat dairy products in reducing the risk of hypertension. However, unlike prior reviews, our umbrella review applied a broader evaluation framework to assess the robustness and credibility of the evidence.

For total dairy and hypertension, the 95% prediction interval (0.73–1.09) included the null, suggesting uncertainty in generalizability to future populations (Supplementary Table S10). No small-study effects (Egger’s test, *p* = 0.76) or excess significance bias were detected, and the largest study was statistically significant with high power (99%), strengthening confidence in the pooled estimate. This association was rated as *suggestive* based on the credibility criteria, indicating moderate but not definitive evidence of a protective effect. For low-fat dairy and hypertension, the 95% prediction interval (0.81–0.94) excluded the null, suggesting consistency across studies (Supplementary Table S10). Again, no evidence of bias was observed, and the largest study had adequate power (70%). Despite this, the credibility was rated as *weak* due to limited study numbers, warranting cautious interpretation. In contrast, high-fat dairy showed no significant association with hypertension (RR = 0.96), with a prediction interval (0.90–1.03) that included the null, no bias detected, and low power in the largest study (13%) (Supplementary Table S10). This association was not considered credible, indicating that any potential effect of high-fat dairy is likely negligible or inconsistent.

The lack of association with high-fat dairy may reflect differences in nutritional profiles, particularly the potential adverse effects of saturated fats. This is further supported by evidence from the National Heart, Lung, and Blood Institute Family Heart Study, which found an inverse association between dairy consumption and prevalent hypertension [96]. Notably, this association was primarily observed among individuals with lower saturated fat intake, which highlights the potential benefits of low-fat dairy products in preventing hypertension [96]. These findings support the idea that the protective effects of dairy may be more pronounced when saturated fat intake is limited, which strengthens dietary recommendations that promote low-fat dairy consumption as part of a heart-healthy diet [97]. Additionally, the bioavailability of calcium, which is a key nutrient in dairy thought to influence blood pressure regulation, may be compromised in the presence of high dietary fat [98]. The capacity of calcium to form insoluble soaps increases with higher fat intake, which means whole-fat dairy products might hinder calcium absorption and therefore reduce its physiological benefits [98]. Additional evidence on the impact of consuming a high-fat diet further supports this view [99]. A randomized, repeated-measures, crossover study found that even a single high-fat meal containing 17 g of saturated fat can temporarily impair vascular function and increase cardiovascular reactivity to stress [99]. Compared to an isocaloric low-fat meal containing 0.8 g saturated fat, participants who consumed the high-fat meal showed significantly higher increases in both systolic and diastolic blood pressure, as well as total peripheral resistance [99]. These findings suggest that consuming saturated fat can negatively affect vascular responses, shedding light on how high-fat dairy might counteract the benefits usually linked to dairy nutrients. Thus, reducing saturated fat intake by choosing low-fat dairy options may be essential to realizing the cardiovascular benefits of dairy consumption.

Several biological mechanisms may explain the protective effects of total and low-fat dairy products on hypertension risk. As discussed earlier, dairy products are rich sources of essential nutrients such as calcium, potassium, and magnesium, which are known to support healthy blood pressure regulation [68,69,78]. Low calcium intake can stimulate parathyroid hormone (PTH) and calcitriol production, both of which increase intracellular calcium in vascular smooth muscle cells, raising vascular reactivity and blood pressure [100]. Additionally, PTH can trigger renin release and elevate levels of angiotensin II and aldosterone, contributing further to hypertension [100]. However, results from the National Heart, Lung, and Blood Institute Family Heart Study revealed no association between calcium intake and hypertension [96]. Additionally, the association between dairy intake and hypertension was not mediated through dietary calcium, suggesting the possible involvement of other nutrients (e.g., potassium and magnesium) in the observed association [96]. Potassium aids in vasodilation and promotes sodium excretion, which both can help with lowering blood pressure [20]. Magnesium also plays a critical role in vascular tone and contractility, and its deficiency has been linked to oxidative stress, inflammation, endothelial dysfunction, and insulin resistance—all of which can contribute to elevated blood pressure [21]. Further, bioactive peptides formed during the digestion of dairy proteins have been shown to exert angiotensin I-converting enzyme (ACE)-inhibitory effects, similar to the action of some antihypertensive medications [101,102]. Low-fat dairy products, in particular, provide these beneficial nutrients without the potentially harmful saturated fats found in high-fat dairy. Together, these mechanisms support the observed associations between low-fat dairy intake and reduced hypertension risk.

***Milk Consumption:*** Our updated meta-analysis of 10 prospective cohort studies involving 249,450 participants and 90,396 cases found that milk consumption was significantly associated with a lower risk of hypertension (RR = 0.947, 95% CI: 0.902, 0.995, *p* = 0.034). However, heterogeneity among studies was considerable (*I*^2^ = 79%), suggesting variability in study design, populations, or milk intake measurements (Figure 2, Supplemental Figure S18). Our findings are consistent with those of prior meta-analyses [24,43,55,58]. Chen et al. (2022) reported a similar inverse association in their high vs. low intake analysis, though their dose–response analysis did not reach statistical significance [24]. This protective association is further supported by Heidari et al. (2021) [55] and Feng et al. (2022) [43], who found significant inverse relationships between milk intake and hypertension risk. Earlier findings from Ralston et al. (2012) provide additional support, with fluid dairy foods (milk and yogurt) associated with reduced hypertension risk [58]. Despite variation in analytical approaches and definitions across these studies, the direction and magnitude of effect estimates are consistently protective. Taken together, the evidence suggests that milk consumption may be modestly beneficial in reducing the risk of hypertension.

However, our findings revealed that the 95% prediction interval (0.86–1.04) included the null, suggesting that future studies may not consistently replicate the observed association (Supplementary Table S10). No small-study effects were detected (Egger’s test *p* = 0.69), and there was no evidence of excess statistical significance, reducing concerns about publication bias. The largest study had high power (99%) and a statistically significant result, supporting the observed effect. Nonetheless, given the high heterogeneity, null-inclusive prediction interval, and modest effect size, the association was rated as having *weak* credibility, indicating that while a potential protective effect exists, the evidence is not sufficiently robust to draw firm conclusions.

***Yogurt Consumption*:** In our meta-analysis of five prospective cohort studies including 735,034 participants and 105,362 cases, yogurt consumption was not significantly associated with hypertension risk (RR = 0.97, 95% CI: 0.86, 1.08, *p* = 0.43). Heterogeneity was considerable (*I*^2^ = 84.3%), which suggests meaningful variability across studies (Figure 2, Supplemental Figure S19). The 95% prediction interval (0.72–1.30) also spanned the null, underscoring the uncertainty in extrapolating the findings to future studies (Supplementary Table S10). Although the largest study was statistically significant and had moderate power (51%), the detection of excess statistical significance raises concerns about potential reporting biases. Due to the non-significant pooled estimate, high heterogeneity, and possible bias, the association was rated as having *non-significant* credibility, suggesting the current evidence does not support a reliable link between yogurt consumption and hypertension risk. This finding aligns with the mixed evidence reported in earlier meta-analyses. Chen et al. (2022) found a borderline inverse association in their high vs. low intake analysis and a similar trend in their dose–response analysis [24]. However, in both analyses, the confidence intervals overlapped the null, and heterogeneity was high, which shows inconsistency in the strength of association [24]. Similarly, Heidari et al. (2021) [55] and Feng et al. (2022) [43] reported no significant association between yogurt intake and hypertension risk. Taken together, the results suggest that yogurt consumption is not significantly associated with hypertension risk. The wide confidence intervals and high between-study heterogeneity highlight the need for more consistent and rigorous research designs.

As we previously showed, high-fat dairy consumption is not associated with a reduction in hypertension risk—unlike low-fat dairy, which has demonstrated protective effects. This difference may help explain the lack of observed anti-hypertensive effects for yogurt. Many commercially available yogurts, especially full-fat or flavored varieties, contain high levels of saturated fat and sugar, which have been linked to increased vascular resistance and elevated blood pressure [99,103]. It is therefore possible that the saturated fat content in certain types of yogurt may offset the potential benefits of its nutrients. Given these considerations, subgroup analyses are needed to distinguish between low-fat and high-fat yogurt varieties in relation to hypertension risk.

### 3.2. Bone Health Outcomes

**Figure 3:**
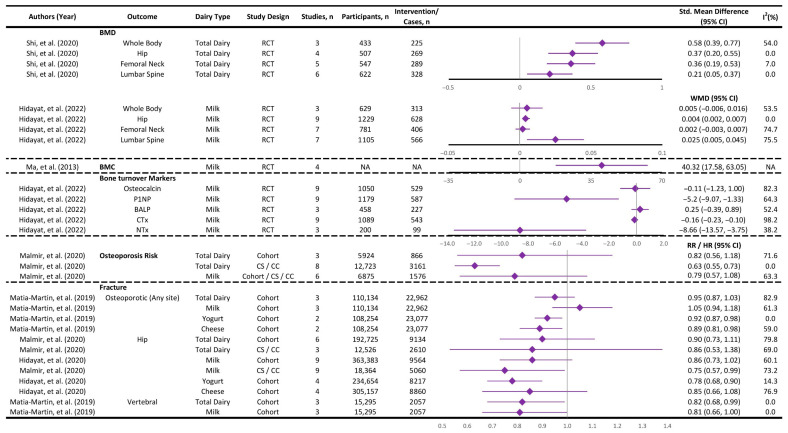
Association between dairy consumption and bone health outcomes, as reported in the most recent literature [33,34,35,45,49,50]. BMD: Bone Mineral Density; BMC: Bone Mineral Content; RCT: Randomized Clinical Trial; CS: Cross-Sectional; CC: Case–Control; WMD: Weighted Mean Difference; RR: Relative Risk; HR: Hazard Ratio.

This umbrella review examined six systematic reviews and meta-analyses investigating the relationship between dairy consumption and bone health (Supplementary Table S12). A meta-analysis could not be conducted due to substantial heterogeneity in study designs (e.g., cohort studies vs. clinical trials), outcome measures, and methodological variability across the included reviews. Reported outcomes included BMD, bone mineral content (BMC), osteoporosis risk, fractures at various sites, and bone turnover markers. Specifically, three studies assessed BMD [33,34,49], one study assessed BMC [49], one study evaluated the risk of osteoporosis [35], three studies analyzed fracture risk [35,45,50], and two studies investigated markers of bone turnover [33,49]. In terms of exposure, three studies included total dairy [34,35,50], five examined milk [33,35,45,49,50], and two focused on yogurt and cheese [45,50]. Although the included studies were generally of moderate to high quality based on JBI assessments, the variability limited quantitative synthesis. Figure 3 shows the most recent meta-analysis evidence on the associations between each exposure (dairy type) and bone health outcome.

#### 3.2.1. Bone Mineral Density (BMD) and Bone Mineral Content (BMC)

A growing body of evidence from RCTs and observational studies suggests that dairy consumption, particularly milk, may have a beneficial impact on BMD and BMC [33,34,49]. The most consistent evidence for a positive effect of dairy intake on BMD comes from RCTs included in meta-analyses by Hidayat et al. (2022) [33] and Shi et al. (2020) [34] (Figure 3, Supplementary Table S12). Hidayat et al. found that milk supplementation led to small but statistically significant improvements in hip BMD (mean difference: 0.004 g/cm^2^; 95% CI: 0.002 to 0.007) and lumbar spine BMD (0.025 g/cm^2^; 95% CI: 0.005 to 0.045) [33]. However, no significant effects were observed for whole-body or femoral neck BMD. These changes, although statistically significant, were small and may not translate into meaningful clinical outcomes. Moreover, subgroup analyses highlighted slightly larger effects in postmenopausal women and Asian populations, especially at the lumbar spine [33]. In line with these findings, Shi et al. (2020) reported standardized mean differences (SMD) for BMD gains across four skeletal sites [34]. The greatest increase was seen in total body BMD (SMD = 0.58; 95% CI: 0.39, 0.77), with smaller yet statistically significant improvements at the total hip (0.37), femoral neck (0.36), and lumbar spine (0.21). These findings suggest that dairy consumption may moderately benefit BMD, as there was consistency across skeletal sites and generally low statistical heterogeneity among the included studies [34]. In contrast, Ma et al. (2013) reported no statistically significant improvement in total body BMD in their pooled analysis [49]. However, when studies using calcium-fortified milk or those with large sample sizes were excluded, small but significant improvements in BMD were observed. In the same meta-analysis, milk consumption was associated with a significant increase in total body BMC [49]. This effect persisted even after excluding calcium-fortified milk studies or those with large sample sizes, which indicates a robust finding. In summary, the collective evidence from these meta-analyses suggests that dairy intake, and milk consumption in particular, exerts a beneficial effect on BMD.

Bone is constantly undergoing remodeling—a balance between resorption (breakdown by osteoclasts) and formation (by osteoblasts) [104]. The beneficial effects of dairy on BMD are often attributed to its high calcium content, a mineral essential for bone remodeling [105]. In addition, adequate calcium intake suppresses circulating levels of PTH, which plays a key role in calcium homeostasis [106]. Chronically elevated PTH can lead to increased bone resorption, resulting in reduced bone mass [106]. By providing a readily bioavailable source of calcium, dairy intake may help lower PTH levels and thereby reduce bone turnover. Supporting this, results from a meta-analysis revealed that milk consumption was associated with a greater reduction in PTH concentrations compared to controls [33]. However, evidence from subgroup analyses by Hidayat et al. (2022) showed that the positive effects on BMD did not significantly differ between milk with high versus low calcium content [33]. This finding suggests that components beyond calcium may also contribute to the observed benefits. Dairy products are rich in other bone-supportive nutrients, including phosphorus [107], magnesium [108], potassium [109], and high-quality protein [110], all of which play roles in bone formation and metabolism. In particular, the presence of bioactive peptides, lactose, and vitamin D (in fortified products) may enhance calcium absorption and retention [111,112]. Furthermore, dairy protein has been shown to stimulate the production of insulin-like growth factor 1 (IGF-1) [113], a hormone that promotes bone formation and growth [114]. Supporting this, the meta-analysis by Hidayat et al. (2022) found that milk consumption was associated with a significant increase in circulating IGF-1 concentrations compared to controls [33]. Taken together, these findings suggest that the beneficial effects of dairy on bone health are likely mediated by multiple mechanisms, which act synergistically to support bone accrual and maintenance.

#### 3.2.2. Osteoporosis Risk

Evidence from observational studies has provided mixed results regarding the association between dairy intake and the risk of osteoporosis [35] (Supplementary Table S12). Malmir et al. (2020) found no significant association between total dairy intake and osteoporosis risk in cohort studies (RR = 0.82; 95% CI: 0.56, 1.18), although a significant inverse association was found in older adults aged >70 years (RR = 0.69; 95% CI: 0.48, 0.98) [35]. Interestingly, findings from cross-sectional and case–control studies revealed that total dairy intake was associated with a 37% reduction in osteoporosis risk (RR = 0.63; 95% CI: 0.55, 0.73), with no between-study heterogeneity [35]. Moreover, Malmir et al. (2020) conducted dose–response analyses, which further clarified these findings [35]. While moderate dairy intake (50–250 g/day) was associated with reduced osteoporosis risk, higher intakes (>250 g/day) may increase the risk, which suggests a nonlinear association. As for milk specifically, the results were again mixed. The meta-analysis found no statistically significant association between milk intake and osteoporosis risk in cohort studies (RR = 0.79; 95% CI: 0.57, 1.08) [35]. However, when data from all study types were pooled in a linear meta-regression, each 200 g per day increase in milk intake was associated with a substantial 39% reduction in osteoporosis risk. This effect remained significant when the analysis was restricted to cross-sectional and case–control studies [35].

The observed nonlinear association between dairy intake and osteoporosis risk may be partly explained by biological mechanisms linked to excessive calcium and saturated fat intake. First, when calcium intake from dairy becomes very high, it may lead to competitive absorption with several other nutrients in the gut, where high calcium can interfere with the absorption of magnesium [115] and possibly zinc [116] or iron [117]. Second, higher intakes of full-fat dairy products can contribute to increased saturated fat consumption, which has been associated with chronic low-grade inflammation [118], a known risk factor for bone loss. Inflammatory processes can promote osteoclast activity, increasing bone resorption and potentially offsetting any protective effects of calcium [119]. Thus, even though dairy provides essential bone nutrients, consuming it in excess may shift the physiological balance in ways that are not entirely beneficial.

#### 3.2.3. Osteoporotic Fractures

***Total Dairy Consumption:*** Across the included meta-analyses, total dairy intake did not demonstrate a significant protective effect against fractures, particularly hip fractures (Figure 3, Supplementary Table S12). Malmir et al. (2020) found no association between total dairy intake and hip fracture risk in cohort studies (RR = 0.90; 95% CI: 0.73, 1.11), nor in cross-sectional and case–control studies (RR = 0.86; 95% CI: 0.53, 1.37) [35]. Dose–response analysis showed no significant linear or nonlinear association, although intakes above 400 g/day were non-significantly associated with lower risk [35]. Matía-Martín et al. (2019) also reported no significant association between highest versus lowest total dairy intake and risk of osteoporotic fractures at any site (HR = 0.95; 95% CI: 0.87, 1.03) or hip fracture (HR = 0.87; 95% CI: 0.75, 1.01) [50]. However, when analyzing vertebral fractures, they reported a significant reduction in risk associated with high total dairy intake (HR = 0.82; 95% CI: 0.68, 0.99), based on limited data with low heterogeneity [50]. In addition, the linear dose–response analysis of increased total dairy intake did not reveal a significant relationship with hip fracture risk (HR = 0.98; 95% CI: 0.95, 1.01) [50]. The current evidence suggests that total dairy intake is not significantly associated with a reduced risk of hip or total fractures, based on data from both cohort and case–control studies. However, the high between-study heterogeneity observed in multiple analyses limits the strength of conclusions.

Interestingly, a potential protective effect was observed for vertebral fractures, where Matía-Martín et al. reported a statistically significant 18% reduction in risk (HR = 0.82, 95% CI: 0.68, 0.99) among those with the highest total dairy intake [50]. This site-specific benefit may indicate that dairy’s impact on bone varies by skeletal site. This can be partly explained by the differences in bone composition and structure. Vertebral bones are rich in trabecular (spongy) bone, which is more active in remodeling than the cortical (compact) bone predominant in the hip [120]. As a result, vertebral bone may respond more rapidly to dietary changes, including increased calcium and other nutrients from dairy. Furthermore, vertebral fractures are more strongly associated with underlying BMD, whereas hip fractures are more influenced by falls [121].

***Milk Consumption:*** Findings regarding milk intake and fracture risk were largely inconsistent (Figure 3, Supplementary Table S12). Both Malmir et al. (2020) [35] and Hidayat et al. (2020) [45] reported no significant association between milk consumption and hip fracture in cohort studies (RR = 0.93; 95% CI: 0.75, 1.15; *I*^2^ = 86.7% and RR = 0.86; 95% CI: 0.73, 1.02; *I*^2^ = 60.1%, respectively). However, cross-sectional and case–control studies showed a modest protective effect (RR = 0.75; 95% CI: 0.57, 0.99; *I*^2^ = 73.2%). Paradoxically, dose–response meta-regression indicated that each 200 g/day increase in milk intake was associated with a 9% increase in hip fracture risk (RR = 1.09; 95% CI: 1.07, 1.11) [35]. Similarly, Matía-Martín et al. (2019) found no significant association between high versus low milk intake and risk of osteoporotic fractures (overall HR = 1.05; 95% CI: 0.94, 1.18) and hip fractures (HR = 0.91; 95% CI: 0.69, 1.21) [50]. However, milk intake was non-significantly associated with reduced risk of vertebral fractures (HR = 0.81; 95% CI: 0.66, 1.00). Incremental milk intake was also not associated with hip fracture risk (HR = 1.01; 95% CI: 0.96, 1.06) [50]. The relationship between milk intake and fracture risk remains inconclusive and may even be adverse at higher intake levels. While some observational studies suggest a small benefit, dose–response data indicate that greater milk consumption could be associated with increased hip fracture risk. This paradox may be explained by potential biological mechanisms, such as the high intake of *D*-galactose from milk sugars, which has been implicated in promoting oxidative stress and inflammation—both known to be detrimental to bone health [122].

***Cheese and Yogurt Consumption***: Compared to milk and total dairy, cheese and yogurt showed more consistent protective associations (Figure 3, Supplementary Table S12). Matía-Martín et al. (2019) found that higher cheese consumption was significantly associated with a reduced risk of osteoporotic fractures at any site (HR = 0.89; 95% CI: 0.81, 0.98; *I*^2^ = 59%) and hip fractures (HR = 0.80; 95% CI: 0.62, 1.03; *I*^2^ = 86.5%) [50]. For yogurt, higher intake was associated with a reduced risk of osteoporotic fractures at any site (HR = 0.92; 95% CI: 0.87, 0.98; *I*^2^ = 0%) and a non-significantly reduced hip fracture risk (HR = 0.87; 95% CI: 0.71, 1.05; *I*^2^ = 66.8%) [50]. Incremental intake analyses showed no significant associations but directionally trended toward benefit for both dairy types. Supporting these findings, Hidayat et al. (2020) conducted a meta-analysis of prospective cohort studies and found that higher yogurt consumption was significantly associated with a lower risk of hip fracture (RR = 0.78; 95% CI: 0.68, 0.90; *I*^2^ = 14.3%), while cheese consumption was not significantly associated with hip fracture risk (RR = 0.85; 95% CI: 0.66, 1.08; *I*^2^ = 76.9%) [45].

This is further supported by a large, two-year cluster randomized controlled trial (RCT) involving over 7000 older adults in 60 Australian aged care facilities, which demonstrated that increasing dietary calcium and protein intake via additional dairy servings, including milk, cheese, and yogurt, significantly reduced the incidence of hip fractures by 33%, all fractures by 46%, and falls by 11% compared to usual care [123]. Collectively, these results suggest that cheese and yogurt may offer protective effects against fractures, particularly non-hip fractures. These fermented dairy products differ from milk in their composition, as they are lower in lactose [124], higher in bioactive peptides [125], and may have more favorable effects on gut microbiota and inflammation [126]. Moreover, fermented dairy products enhance the bioavailability of minerals, particularly calcium, by lowering intestinal pH, and SCFAs produced by probiotics further support calcium absorption [126]. However, given the observational nature of the included studies, further research is warranted to confirm these benefits and explore potential mechanisms.

#### 3.2.4. Bone Turnover Markers

Two meta-analyses—Hidayat et al. (2022) [33] and Ma et al. (2013) [49]—have assessed the effects of milk intake on key bone turnover markers, including osteocalcin, procollagen type 1 N-terminal propeptide (P1NP), bone-specific alkaline phosphatase (BALP), C-terminal telopeptide of type I collagen (CTx), and N-terminal telopeptide (NTx) (Supplementary Table S12). The findings suggest that milk consumption may exert favorable effects on bone metabolism, primarily through reduction in bone resorption markers, although the evidence is somewhat mixed for bone formation markers [33,49].

Osteocalcin, P1NP, and BALP are commonly used indicators of bone formation. Osteocalcin showed mixed results across the two meta-analyses. Hidayat et al. (2022) [33] found no significant change in osteocalcin concentrations between milk-supplemented and control groups (mean difference: −0.11 ng/mL; 95% CI: −1.23 to 1.00). However, in postmenopausal women, the reduction approached significance (−3.87 ng/mL; 95% CI: −8.02 to 0.27) [33]. In contrast, Ma et al. (2013) [49] reported a significant reduction in osteocalcin levels associated with milk intake (−5.90 ng/mL; 95% CI: −7.23 to −4.57; *p* < 0.00001). The effect was stronger when calcium-fortified milk was excluded (−7.90 ng/mL) and remained robust across shorter-duration interventions [49]. Subgroup analyses revealed a particularly strong reduction in males (−17.28 ng/mL) and Caucasians (−5.98 ng/mL), while the effect was not significant in Asians or children [49]. P1NP was consistently reduced following milk intake. Hidayat et al. found a significant reduction in P1NP concentrations (−5.20 ng/mL; 95% CI: −9.07 to −1.33) [33]. The reduction was even larger among postmenopausal women (−6.21 ng/mL) and Asian participants (−5.59 ng/mL). When milk provided ≥1000 mg of calcium daily, the P1NP-lowering effect was further enhanced (−6.87 ng/mL; 95% CI: −11.02 to −2.71) [33]. Evidence for BALP was limited and not statistically significant. According to Hidayat et al., there was no significant difference in BALP levels between groups (0.25 μg/L; 95% CI: −0.39 to 0.89) [33], which suggests that milk consumption may have minimal effects on this particular marker of bone formation.

The bone resorption markers—CTx and NTx—consistently showed reductions with milk intake. Hidayat et al. reported a significant reduction in CTx (−0.16 ng/mL; 95% CI: −0.23 to −0.10), with larger effects observed in postmenopausal women (−0.21 ng/mL) and Asian participants (−0.12 ng/mL) [33]. The effect remained significant in calcium-rich milk trials (≥1000 mg/day: −0.15 ng/mL; 95% CI: −0.20 to −0.10). In the same meta-analysis, NTx decreased by −8.66 nmol BCE/mmol creatinine (95% CI: −13.57 to −3.75), and by −7.94 among postmenopausal women [33]. Ma et al. confirmed this effect (−5.41 nmol/mmol; 95% CI: −10.35 to −0.47; *p* = 0.03). When studies on calcium-fortified milk were excluded, the reduction was more pronounced (−18.91 nmol/mmol; 95% CI: −33.73 to −4.09) [49]. Therefore, milk consumption appears to have small yet consistent effects in reducing bone resorption markers, which suggests a potential benefit in slowing bone loss. The evidence for bone formation markers appears to be more variable, as osteocalcin showed mixed results, P1NP consistently decreased, and BALP was unaffected. These findings indicate that milk intake may primarily attenuate bone turnover by suppressing resorption rather than stimulating formation. It is also important to note that the benefit was more apparent when calcium fortification was excluded, which suggests a potential independent effect of milk proteins or other bioactive components.

***Strengths and Limitations:*** This umbrella review has several important strengths. First, rather than just evaluating dairy intake as a single, aggregated dietary exposure, we also conducted separate analyses for specific dairy products, including milk, yogurt, and butter, as well as for subtypes such as low-fat and high-fat dairy, if feasible. This detailed approach allowed for a better understanding of how individual dairy components may differently influence CVD and bone health outcomes. Second, the review takes a dual-focus approach by examining both cardiovascular disease and osteoporosis—two major non-communicable diseases that share overlapping risk factors and physiological mechanisms [9,12,13]. Third, we provide an up-to-date synthesis of the evidence on dairy consumption and cardiovascular disease by conducting an updated meta-analysis of prospective cohort studies. In contrast to conventional umbrella reviews that rely solely on previously published meta-analyses, we systematically identified and incorporated newly published prospective studies into the existing evidence base, ensuring a more current and comprehensive analysis. Finally, to ensure methodological rigor, all included meta-analyses were critically appraised using the JBI tool. This quality assessment supports the credibility of our findings and the robustness of our conclusions.

Despite its strengths, this umbrella review has several limitations that should be acknowledged. First, although we included only meta-analyses that were rated as moderate to high quality using the JBI tool, the overall strength of the evidence for cardiovascular outcomes remains limited. Notably, none of the observed associations met the criteria for convincing or highly suggestive evidence. This may be attributed to considerable heterogeneity across studies, the presence of small-study effects, and limited statistical power in the largest contributing cohorts. Second, for bone health outcomes, the wide variation in outcome definitions, population characteristics, and analytical methods across existing meta-analyses posed a significant challenge. These inconsistencies prevented us from conducting updated re-analyses, which in turn limited our ability to synthesize findings or draw definitive conclusions in this domain. Third, it is important to note that all meta-analyses assessing cardiovascular outcomes were derived from observational cohort studies. While such studies are valuable for identifying associations, they limit our ability to establish causal relationships due to potential residual confounding and reverse causation. RCTs of dairy intake and cardiovascular outcomes are scarce, and none meeting our inclusion criteria were identified. Similarly, Mendelian randomization (MR) studies—although promising for causal inference—were not included, as our protocol focused exclusively on meta-analyses of empirical studies. Future umbrella reviews incorporating MR analyses and pooled RCT data, where available, could help clarify causality and strengthen the evidence base. Fourth, we did not conduct dose–response analyses within this review. Although several of the included studies reported dose–response relationships, synthesizing these data across exposures and outcomes was not feasible due to variation in reported units and categorization. Finally, while we aimed to differentiate between types of dairy products, the available evidence often lacked detailed information on specific subtypes of milk and yogurt, particularly in terms of nutrient composition, fat content, and processing. This lack of information, along with the limited number of studies for certain subgroups, restricted our ability to perform a stratified analysis which may have provided further insights into the role of dairy subtypes in disease risk.

***Implications and Future Research:*** The findings of this umbrella review have important implications for public health and clinical nutrition. The observed inverse associations between total dairy, milk, and yogurt consumption and CVD outcomes, particularly stroke and hypertension, suggest that incorporating dairy products into the diet may contribute to cardiovascular risk reduction. However, the modest effect sizes and lack of strong associations for CHD call for cautious interpretation. The evidence highlights the need for more specific dietary guidance that distinguishes between different types of dairy products rather than treating them as a homogenous entity. In particular, the differential associations observed across types of dairy products (e.g., milk, yogurt, butter) and their fat content (e.g., low-fat vs. high-fat) highlight the importance of specificity in dietary recommendations. Emphasizing low-fat dairy products in public health guidelines could allow individuals to gain cardiovascular and bone health benefits whilst limiting the potential adverse effects of saturated fat. Tailored dietary strategies may also be warranted based on individual health profiles and disease risk factors. Further, because most meta-analyses lacked stratification by specific saturated fat content, dairy processing methods, or population-level dietary patterns, future studies with harmonized definitions and detailed dietary data are needed to more comprehensively assess these factors and clarify the mechanisms underlying differential effects. Moreover, future meta-analyses that integrate stratified results by age, sex, or baseline disease risk could provide valuable insight into whether specific subpopulations derive greater benefit from dairy consumption in relation to cardiovascular outcomes. Finally, we excluded fortified and probiotic-enriched dairy products from our synthesis to maintain focus on conventional dairy and avoid conflating effects of added bioactive components with those of the dairy matrix itself. Future umbrella reviews or updates could incorporate such products, as they may confer different or additional cardiovascular and bone health benefits compared with conventional dairy.

The inconclusive findings regarding bone health outcomes emphasize the need for more high-quality, well-controlled studies in this domain. Future research should aim to address inconsistencies in outcome definitions and population characteristics and explore potential differential effects of individual dairy products and their components (e.g., saturated fat). Long-term RCTs examining clinical endpoints such as fracture incidence and osteoporosis diagnoses are especially needed. Moreover, dose–response relationships remain underexplored, particularly in the context of subtypes of dairy and specific disease outcomes. Future meta-analyses and primary studies should incorporate standardized exposure assessments and stratify findings by fat content, fermentation status, and population demographics (e.g., age, sex, baseline health status) to better inform personalized nutrition advice. Only two meta-analyses have quantitatively examined the impact of dairy intake on bone turnover markers, and both reported considerable variability in biomarker selection, assay methodology, and units of measurement [33,49]. This lack of standardization reduces the ability to compare results across studies and limits the feasibility of large-scale meta-analyses. Future research would benefit from adopting internationally recognized biomarker panels (e.g., those recommended by the International Osteoporosis Foundation), with standardized sample collection protocols, assay methods, and uniform reporting units. Finally, future research should also assess dairy consumption within the context of overall dietary patterns rather than in isolation. As nutritional epidemiology moves toward examining whole dietary patterns, it is important to consider how dairy interacts with other foods and nutrients. This approach may offer a more accurate understanding of dairy’s impact on overall diet quality and health outcomes.

## 4. Conclusions

This umbrella review and updated meta-analysis provide a comprehensive assessment of the associations between dairy product consumption and CVD and bone health outcomes. Overall, higher consumption of total dairy, milk, and yogurt was associated with modest but statistically significant reductions in CVD risk, particularly for stroke and hypertension. Both high-fat and low-fat dairy were inversely associated with stroke risk, while low-fat dairy demonstrated a protective association against hypertension. However, findings for CHD were more heterogeneous. While milk consumption was slightly associated with increased CHD risk, no significant associations were observed for total dairy, yogurt, or butter. In contrast, the evidence linking dairy consumption to bone health outcomes remains inconclusive. Although several studies suggest beneficial effects of dairy—especially milk—on BMD, osteoporosis risk, and bone metabolism, the associations with fracture risk, particularly hip fractures, were inconsistent. Cheese and yogurt appeared more consistently protective in some analyses, but the overall body of evidence does not support a definitive protective effect of dairy intake against fractures. Importantly, because CVD and osteoporosis share several common risk factors and underlying mechanisms, including inflammation, oxidative stress, and metabolic dysregulation [9,12,13], it is plausible that dairy products exert protective effects on both conditions through overlapping biological pathways.

Taken together, these findings emphasize the importance of distinguishing between different types of dairy products, such as milk, yogurt, and butter, as well as by fat content (e.g., high-fat vs. low-fat dairy), both in research and in dietary recommendations. In particular, dietary guidance should emphasize the consumption of low-fat dairy products, which may offer the health benefits associated with dairy intake while minimizing the potential harms related to saturated fat. Tailoring recommendations in this way can help optimize the protective effects of dairy consumption across multiple health domains.

## Data Availability

Data sharing is not applicable to this article as no new data were created or analyzed in this study.

## Supplementary Materials

The following supporting information can be downloaded at https://www.mdpi.com/article/10.3390/nu17172723/s1: Supplementary Table S1. Search strategy for systematic reviews and meta-analyses on dairy consumption and cardiovascular and bone health outcomes; Supplementary Table S2. Agreed upon critical appraisal scores of studies which passed initial title and abstract screening; Supplementary Table S3. Detailed assessment of methodological quality of the studies identified through hand searching using the Newcastle–Ottawa Scale; Supplementary Table S4. Characteristics of the original studies on the association between dairy consumption and cardiovascular outcomes, identified through hand-searching, included in the updated meta-analyses; Supplementary Table S5. Detailed description of the original studies on the association between dairy consumption and cardiovascular outcomes, identified through hand-searching, included in the updated meta-analyses; Supplementary Table S6. Characteristics of meta-analyses on dairy consumption and cardiovascular and bone health outcomes included in the umbrella review; Supplementary Table S7. List of studies excluded from the umbrella review and reasons for exclusion; Supplementary Table S8. The detailed assessments of methodological quality of the included systematic reviews and meta-analyses; Supplementary Table S9. Description of meta-analyses on dairy consumption and cardiovascular outcomes included in the umbrella review; Supplementary Table S10. Association between dairy consumption (highest vs. lowest intake level) and cardiovascular outcomes; Supplementary Table S11. Adjusted relative risk for milk and CVD and milk and stroke using two methods of SSE correction; Supplementary Table S12. Description of meta-analyses on dairy consumption and bone health outcomes included in the umbrella review; Supplementary Figure S1. Association between total dairy consumption (highest vs. lowest intake level) and CVD risk; Supplementary Figure S2. Association between milk consumption (highest vs. lowest intake level) and CVD risk; Supplementary Figure S3. Association between yogurt consumption (highest vs. lowest intake level) and CVD risk; Supplementary Figure S4. Association between total dairy consumption (highest vs. lowest intake level) and CHD risk; Supplementary Figure S5. Association between high-fat dairy consumption (highest vs. lowest intake level) and CHD risk; Supplementary Figure S6. Association between low-fat dairy consumption (highest vs. lowest intake level) and CHD risk; Supplementary Figure S7. Association between milk consumption (highest vs. lowest intake level) and CHD risk; Supplementary Figure S8. Association between yogurt consumption (highest vs. lowest intake level) and CHD risk; Supplementary Figure S9. Association between butter consumption (highest vs. lowest intake level) and CHD risk; Supplementary Figure S10. Association between total dairy consumption (highest vs. lowest intake level) and stroke risk; Supplementary Figure S11. Association between high-fat dairy consumption (highest vs. lowest intake level) and stroke risk; Supplementary Figure S12. Association between low-fat dairy consumption (highest vs. lowest intake level) and stroke risk; Supplementary Figure S13. Association between milk consumption (highest vs. lowest intake level) and stroke risk; Supplementary Figure S14. Association between yogurt consumption (highest vs. lowest intake level) and stroke risk; Supplementary Figure S15. Association between total dairy consumption (highest vs. lowest intake level) and hypertension; Supplementary Figure S16. Association between high-fat dairy consumption (highest vs. lowest intake level) and hypertension; Supplementary Figure S17. Association between low-fat dairy consumption (highest vs. lowest intake level) and hypertension; Supplementary Figure S18. Association between milk consumption (highest vs. lowest intake level) and hypertension; Supplementary Figure S19. Association between yogurt consumption (highest vs. lowest intake level) and hypertension. References [127,128,129,130,131,132,133,134,135,136,137,138,139,140,141,142,143,144,145,146,147,148,149,150,151,152] are cited in the supplementary materials.

## Abbreviations

The following abbreviations are used in this manuscript:

CVD	Cardiovascular disease
CHD	Coronary heart disease
PRISMA	Preferred Reporting Items for Systematic Reviews and Meta-Analyses
JBI	Joanna Briggs Institute
NOS	Newcastle–Ottawa Scale
RR	Relative risks
HR	Hazard ratios
CI	Confidence intervals
SE	Standard error
LTPs	Lactotripeptides
SCFAs	Short-chain fatty acids
PTH	Parathyroid hormone
ACE	Angiotensin I-converting enzyme (ACE)
BMC	Bone mineral content
BMD	Bone mineral density
P1NP	Procollagen type 1 N-terminal propeptide
BALP	Bone-specific alkaline phosphatase
CTx	C-terminal telopeptide of type I collagen
NTx	N-terminal telopeptide
MR	Mendelian randomization
