# Supplementary material for: Dairy Consumption and Risk of Cardiovascular and Bone Health Outcomes in Adults: An Umbrella Review and Updated Meta-Analyses
Source: Nutrients. 2025 Aug 22;17(17):2723. doi: 10.3390/nu17172723 (PMC12430323; doi:10.3390/nu17172723)
Supplement: Supplementary file 1 [file nutrients-17-02723-s001.zip › nutrients-3810563-supplementary.pdf]

Supplementary Table S1. Search strategy for systematic reviews and meta-analyses on dairy consumption and cardiovascular and bone health outcomes

**Descriptors**	**Key Words**	**MeSH Terms**
Diet	dairy, milk, cream, cheese, ice cream, butter, yogurt, yoghurt, yoghourt, buttermilk, sour cream, fermented dairy, fermented milk products, kefir, koumiss, ghee, quark, skyr, viili, ymer, zincica	Dairy Products (explode)
Disease	osteoporosis, bone loss, osteopenia, 'osteoporotic fracture', 'osteopenic fracture'	Osteoporosis (explode)
	arterial occlusive diseases, cardiac arrest, cerebrovascular disorders, coronary artery disease, diabetic angiopathies, heart failure, hyperemia, hypertension, hypotension, ischemic colitis, mesenteric ischemia, myocardial ischemia, peripheral vascular diseases, prehypertension, pulmonary heart disease, reperfusion injury, retinal vein occlusion, spinal cord vascular diseases, varicose veins, ventricular dysfunction, venous insufficiency	Cardiovascular Diseases [with hierarchy restrictions representing key word list]
Study Design	meta-analysis, systematic review, systematic literature review, systematic scoping review, systematic narrative review, systematic qualitative review, systematic evidence review, systematic mixed studies review, systematic mapping review, systematic Cochrane review, systematic search and review, systematic integrative review	Meta-Analysis, 'Meta-Analysis as Topic', 'Systematic Review'

Supplementary Table S2. Agreed upon critical appraisal scores of studies which passed initial title and abstract screening

Authors, year	Title	Critical appraisal score
**CVD outcomes**		
Alexander, et al. (2016) [1]	Dairy consumption and CVD: a systematic review and meta-analysis	Moderate quality (7/11)
Bechthold, et al. (2019) [2]	Food groups and risk of coronary heart disease, stroke and heart failure: A systematic review and dose-response meta-analysis of prospective studies	Moderate quality (8/11)
Bhandari, et al. (2023) [3]	Long-term consumption of 10 food groups and cardiovascular mortality: A systematic review and dose response meta-analysis of prospective cohort studies	Moderate quality (9/11)
Chen, et al. (2017) [4]	Cheese consumption and risk of cardiovascular disease: a meta-analysis of prospective studies	Moderate quality (9/11)
Chen, et al. (2022) [5]	Dairy product consumption and cardiovascular health: a systematic review and meta-analysis of prospective cohort studies	High quality (10/11)
Feng, et al. (2022) [6]	Consumption of dairy products and the risk of overweight or obesity, hypertension, and type 2 diabetes mellitus: A dose-response meta-analysis and systematic review of cohort studies	High quality (10/11)
Gao, et al. (2020) [7]	Yogurt intake reduces all-cause and cardiovascular disease mortality: a meta-analysis of eight prospective cohort studies	Low quality (5/11)*
Gholami, et al. (2017) [8]	The effect of dairy consumption on the prevention of cardiovascular diseases: A meta-analysis of prospective studies	Moderate quality (9/11)
Guo, et al. (2017) [9]	Milk and dairy consumption and risk of cardiovascular diseases and all-cause mortality: dose-response meta-analysis of prospective cohort studies	High quality (10/11)
Heidari, et al. (2021) [10]	Dairy products consumption and the risk of hypertension in adults: An updated systematic review and dose-response meta-analysis of prospective cohort studies	Moderate quality (7/11)
Jakobsen, et al. (2021) [11]	Intake of dairy products and associations with major atherosclerotic cardiovascular diseases: a systematic review and meta-analysis of cohort studies	Moderate quality (9/11)
Kazemi, et al. (2023) [12]	The relationship between major food sources of fructose and cardiovascular disease, cancer, and all-cause mortality: a systematic review and dose-response meta-analysis of cohort studies	High quality (10/11)
Mazidi, et al. (2019) [13]	Consumption of dairy product and its association with total and cause specific mortality - A population-based cohort study and meta-analysis	High quality (10/11)
Mishali, et al. (2019) [14]	Association between dairy intake and the risk of contracting type 2 diabetes and cardiovascular diseases: a systematic review and meta-analysis with subgroup analysis of men versus women	Moderate quality (7/11)

Mullie, et al. (2016) [15]	Daily milk consumption and all-cause mortality, coronary heart disease and stroke: a systematic review and meta-analysis of observational cohort studies	Moderate quality (8/11)
Naghshi, et al. (2022) [16]	High vs. low-fat dairy and milk differently affects the risk of all-cause, CVD, and cancer death: a systematic review and dose-response meta-analysis of prospective cohort studies	High quality (10/11)
Pimpin, et al. (2016) [17]	Is butter back? A systematic review and meta-analysis of butter consumption and risk of cardiovascular disease, diabetes, and total mortality	High quality (10/11)
Qin, et al. (2015) [18]	Dairy consumption and risk of cardiovascular disease: an updated meta-analysis of prospective cohort studies	High quality (10/11)
Ralston, et al. (2012) [19]	A systematic review and meta-analysis of elevated blood pressure and consumption of dairy foods	Moderate quality (8/10)
Soedamah-Muthu, et al. (2011) [20]	Milk and dairy consumption and incidence of cardiovascular diseases and all-cause mortality: dose-responses meta-analysis of prospective cohort studies	Moderate quality (8/11)
Soedamah-Muthu, et al. (2018) [21]	Dairy consumption and cardiometabolic diseases: systematic review and updated meta-analysis of prospective cohort studies	Low quality (6/11)*
Sun, et al. (2023) [22]	The relationship between major food sources of fructose and cardiovascular outcomes: A systematic review and dose-response meta-analysis of prospective studies	Moderate quality (9/11)
Trieu, et al. (2021) [23]	Biomarkers of dairy fat intake, incident cardiovascular disease, and all-cause mortality: a systematic review and meta-analysis	Low quality (6/11)*
Tutunchi, et al. (2023) [24]	Yogurt consumption and risk of mortality from all causes, CVD and cancer: a comprehensive systematic review and dose-response meta-analysis of cohort studies	Moderate quality (9/11)
Wu, et al. (2017) [25]	Consumption of yogurt and the incident risk of cardiovascular disease: a meta-analysis of nine cohort studies	Moderate quality (9/11)
Zhang, et al. (2020) [26]	Fermented Dairy Foods Intake and Risk of Cardiovascular Diseases: A Meta-analysis of Cohort Studies	Moderate quality (8/11)
**Bone health outcomes**		
Goncerz, et al. (2022) [27]2025-08-22 5:45:00 PM	Higher milk consumption is not associated with fracture risk reduction: systematic review and meta-analysis	Low quality (4/11)*
Hidayat, et al. (2020) [28]	Systematic review and meta-analysis of the association between dairy consumption and the risk of hip fracture: critical interpretation of the currently available evidence	High quality (10/11)
Hidayat, et al. (2022) [29]	The effects of milk supplementation on bone health indices in adults: a meta-analysis of randomized controlled trials	Moderate quality (9/11)

Ma, et al. (2013) [30]	Milk intake increases bone mineral content through inhibiting bone resorption: Meta-analysis of randomized controlled trials	High quality (11/11)
Malmir, et al. (2020) [31]	Consumption of milk and dairy products and risk of osteoporosis and hip fracture: a systematic review and meta-analysis	High quality (10/11)
Matia-Martin, et al. (2019) [32]	Effects of milk and dairy products on the prevention of osteoporosis and osteoporotic fractures in Europeans and Non-Hispanic Whites from North America: A systematic review and updated meta-analysis	High quality (11/11)
Shi, et al. (2020) [33]	Effects of dairy products on bone mineral density in healthy postmenopausal women: a systematic review and meta-analysis of randomized controlled trials	High quality (10/11)

*Excluded from further analysis. CVD: Cardiovascular Disease.

Supplementary Table S3. Detailed assessment of methodological quality of the studies identified through hand searching using the Newcastle–Ottawa Scale

	#1. Representativeness of Cohort	#2. Selection of Non-Exposed Cohort	#3. Ascertainment of Exposure	#4. Outcome Not Present at Start	#5. Comparability of Cohorts	#6. Assessment of Outcome	#7. Follow-Up Long Enough	#8. Adequacy of Follow-Up	#9. Total Score	Quality Class
Dukuzimana, et al. (2024) [34]	1	1	1	0	1	2	1	1	8	High
Guo, et al. (2022) [35]	1	1	1	0	1	1	1	1	7	High
Koskinen, et al. (2018) [36]	1	1	1	1	2	1	1	1	9	High
Olsson, et al. (2022) [37]	1	1	1	0	2	1	1	1	8	High
Sellem, et al. (2022) [38]	1	0	1	1	2	1	1	1	8	High
Talaei, et al. (2017) [39]	1	0	1	1	1	2	1	1	8	High
Um, et al. (2019) [40]	1	1	1	0	1	1	1	1	7	High
Wang, et al. (2020) [41]	1	1	1	0	2	1	1	1	8	High

Zhou, et al. (2023) [42]	1	1	1	0	2	1	1	1	8	High

Supplementary Table S4. Characteristics of the original studies on the association between dairy consumption and cardiovascular outcomes, identified through hand-searching, included in the updated meta-analyses.

Authors, year	Title	Study type	Objective	Exposure assessed	Outcomes assessed	Study population
Dukuzimana, et al. (2024) [34]	High consumption of dairy products and risk of major adverse coronary events and stroke in a Swedish population	Cohort	To examine the association between intake of dairy products and risk of major adverse coronary events and stroke	Non-fermented milk, fermented milk, cheese, butter, and cream	Major adverse coronary events, CHD, stroke	Adults aged 44–74 years
Guo, et al. (2022) [35]	Association between dairy consumption and cardiovascular disease events, bone fracture and all-cause mortality	Cohort	To investigate the association of total dairy, fermented dairy, and dairy subtypes with CVD, CHD, fracture, and all-cause mortality	Total dairy, fermented dairy, milk (low-/high-fat), yogurt, cheese, butter, cream	CVD, CHD, fracture, all-cause mortality	Adults aged 30–60 years
Koskinen, et al. (2018) [36]	Intake of fermented and non-fermented dairy products and risk of incident CHD: the Kuopio Ischaemic Heart Disease Risk Factor Study	Cohort	To investigate whether fermented and non-fermented dairy products have distinct associations with the risk of incident CHD	Fermented and non-fermented dairy products (including low-fat and high-fat subtypes), total milk, cheese, butter	CHD	Adult men aged 42–60 years

Olsson, et al. (2022) [37]	Milk and Fermented Milk Consumption and Risk of Stroke: Longitudinal Study	Cohort	To investigate associations of milk and fermented milk consumption with risk of stroke subtypes	Milk and fermented milk intake	Total stroke, cerebral infarction, hemorrhagic stroke	Adults
Sellem, et al. (2022) [38]	Consumption of dairy products and CVD risk: results from the French prospective cohort NutriNet-Santé	Cohort	To investigate the associations between consumption of dairy foods (overall and specific types) and cardiovascular disease (CVD) risk in a large cohort of French adults	Total dairy intake and types of dairy: milk, cheese, yogurts, fermented dairy, non-fermented dairy, reduced-fat and high-fat dairy	Total CVD, CHD, and stroke	Adults aged ≥18 years
Talaei, et al. (2017) [39]	The association between dairy product intake and cardiovascular disease mortality in Chinese adults	Cohort	To evaluate the relation of dairy intake with risk of cardiovascular disease (CVD) mortality in middle-aged and elderly Chinese in Singapore	Total dairy intake, including milk, Milo/Ovaltine/Horlicks, Yakult/Vitagen, butter, ice cream	CVD, CHD, and Stroke mortality	Adults aged 45–74 years
Um, et al. (2019) [40]	Associations of calcium and dairy product intakes with all-cause, all-cancer, colorectal cancer and CHD mortality among older women in the Iowa	Cohort	To examine associations between calcium and dairy product intakes and mortality outcomes (all-cause, cancer, CHD)	Total, dietary, and supplemental calcium; total dairy, milk (whole, low-/non-fat), yogurt, cheese	all-cancer, CHD mortality	Older women aged 55–69 years

	Women's Health Study					
Wang, et al. (2020) [41]	Milk consumption and risk of mortality from all-cause, cardiovascular disease and cancer in older people	Cohort	To examine the association between milk consumption and the risk of all-cause, CVD, and cancer mortality in older Chinese adults	Milk consumption (none, moderate [1–3 portions/week], high [≥3 portions/week])	All-cause, CVD, CHD, stroke, and cancer mortality	Older adults aged ≥50 years
Zhou, et al. (2023) [42]	Association of milk consumption with all-cause mortality and cardiovascular outcomes: a UK Biobank based large population cohort study	Cohort	To examine the association of different milk types (full cream, semi-skimmed, skimmed, soy) with all-cause and CVD outcomes	Full cream milk, semi-skimmed milk, skimmed milk, soy milk, other milk	All-cause mortality, CVD mortality, myocardial infarction, stroke, CVD events	Adults aged 40–69 years

CVD: Cardiovascular Disease; CHD: Coronary Heart Disease; UK: United Kingdom.

Supplementary Table S5. Detailed description of the original studies on the association between dairy consumption and cardiovascular outcomes, identified through hand-searching, included in the updated meta-analyses

Authors, year	Outcome	Exposure	Study design	Comparison/Intake	Cases/Total (Highest Vs Lowest)	Metric	Effect size (95% CI)	P value
Dukuzimana, et al. (2024) [34]	CHD	Milk	Cohort	High vs. low	71/392 vs. 1149/11,655	HR	1.30 (1.01–1.68)	0.03
Guo, et al. (2022) [35]	CVD	Total dairy	Cohort	High vs. low intake	200/436 vs. 239/437	HR	1.13 (0.90–1.43)	0.24
	CHD	Total dairy	Cohort	High vs. low	65/436 vs. 105/437	HR	0.65 (0.45–0.95)	0.13
	CVD	Milk	Cohort	High vs. low	196/436 vs. 260/437	HR	0.84 (0.68–1.03)	0.03
	CHD	Milk	Cohort	High vs. low	69/436 vs. 104/437	HR	0.82 (0.59–1.16)	0.04
Koskinen, et al. (2018) [36]	CHD	Total dairy	Cohort	High vs. low	122/495 vs. 112/495	HR	0.97 (0.71–1.34)	0.92
Olsson, et al. (2022) [37]	Stroke	Milk	Cohort	High vs. low	7573 cerebral infarctions, 1470 hemorrhagic strokes /79,618	HR	Cerebral infarction: 1.01 (95% CI: 0.94–1.07)	NA

							Hemorrhagic stroke: 1.19 (95% CI: 1.03–1.36)	
Sellem, et al. (2022) [38]	CVD	Total dairy	Cohort	High vs. low intake	480/25,722 vs 486/25,715	HR	0.99 (0.88–1.13)	0.62
	CHD	Total dairy	Cohort	High vs. low	293/25,908 vs 287/25,914	HR	0.95 (0.80–1.12)	0.30
	Stroke	Total dairy	Cohort	High vs. low	217/25,984 vs 212/25,989	HR	0.93 (0.76–1.13)	0.19
	CVD	Milk	Cohort	High vs. low	486/25,716 vs 484/25,717	HR	1.00 (0.89–1.14)	0.70
	CHD	Milk	Cohort	High vs. low	275/25,926 vs 296/25,905	HR	1.10 (0.93–1.30)	0.40
	Stroke	Milk	Cohort	High vs. low	196/26,005 vs 207/25,994	HR	1.13 (0.92–1.38)	0.19
	CVD	Yogurt	Cohort	High vs. low	473/24,722 vs 530/30,180	HR	1.09 (0.96–1.23)	0.10
	CHD	Yogurt	Cohort	High vs. low	321/25,599 vs 297/30,135	HR	0.87 (0.74–1.02)	0.12
	Stroke	Yogurt	Cohort	High vs. low	235/25,685 vs 187/30,245	HR	0.92 (0.76–1.12)	0.30
Talaei, et al. (2017) [39]	CVD mortality	Total dairy	Cohort	High vs. low intake	1145/219,722 vs 1380/221,744	HR	0.95 (0.87–1.04)	0.64
Um, et al. (2019) [40]	CHD	Total dairy	Cohort	High vs. low intake	756/7467 vs. 733/6976	HR	0.99 (0.88–1.12)	0.65

Wang, et al. (2020) [41]	CVD	Milk	Cohort	High vs. low	NA/18,214	HR	0.72 (0.57–0.92)	
Zhou, et al. (2023) [42]	CVD	Milk	Cohort	Milk users vs. non-milk users	3679/450,507	HR	Full cream: 0.93 (0.77–1.12)<br><br>Semi-skimmed: 0.75 (0.63–0.88)<br><br>Skimmed: 0.74 (0.62–0.88)	Full cream: 0.552<br><br>Semi-skimmed:<br><br>0.007<br><br>Skimmed: 0.003

CVD: Cardiovascular Disease; CHD: Coronary Heart Disease; HR: Hazard Ratio.

Supplementary Table S6. Characteristics of meta-analyses on dairy consumption and cardiovascular and bone health outcomes included in the umbrella review

Authors, year	Title	Study type & search strategy	Objective	Exposure assessed	Outcomes assessed	Study population	Appraisal instrument(s)
**CVD outcomes**							
Alexander, et al. (2016) [1]	Dairy consumption and CVD: a systematic review and meta-analysis	• Systematic review and meta-analysis<br>• Search conducted from inception to March 2015	(i) To estimate summary associations between total dairy intake and specific dairy products and CVD, CHD and stroke; (ii) to conduct sub-group and sensitivity analyses by descriptive study characteristics to identify potential sources of heterogeneity and to evaluate patterns of associations; (iii) evaluate dose-response relationships using categorical intake analyses and linear splines; and (iv) to evaluate the potential for publication bias.	• Total dairy<br>• Milk<br>• Cheese<br>• Yogurt	• Total CVD<br>• Stroke<br>• CHD	Adults	N/A
Bechthold, et al. (2019) [2]	Food groups and risk of coronary heart disease, stroke and heart failure: A systematic review and dose-response meta-analysis of	• Systematic review and dose-response meta-analysis<br>• Search conducted from inception to March 2017	To summarize findings of the association between 12 a priori defined food groups (including whole grains, refined grains, vegetables, fruits, nuts, legumes, eggs, dairy, fish, red meat, processed meat, and sugar-sweetened beverages) with	• Total dairy	• Stroke<br>• CHD	18 years or above; healthy	NutriGrade

	prospective studies		risk of CHD, stroke, and heart failure.				
Bhandari, et al. (2023) [3]	Long-Term Consumption of 10 Food Groups and Cardiovascular Mortality: A Systematic Review and Dose Response Meta-Analysis of Prospective Cohort Studies	• Systematic review and dose-response meta-analysis<br>• Comprehensive search conducted in Medline, Embase, Scopus, CINAHL, and Web of Science until January 2022	To evaluate the relationship between the long-term consumption of 10 food groups and cardiovascular mortality.	• Dairy products	• Cardiovascular mortality	General adult population aged ≥18 years	Newcastle–Ottawa scale
Chen, et al. (2017) [4]	Cheese consumption and risk of cardiovascular disease: a meta-analysis of prospective studies	• Meta-analysis<br>• Search conducted from inception to December 30th, 2015	To evaluate the risks of total CVD, CHD, and stroke associated with cheese consumption.	• Cheese	• Total CVD<br>• Stroke<br>• CHD	Not specified	Newcastle Ottawa Scale
Chen, et al. (2022) [5]	Dairy product consumption and cardiovascular health: a systematic review and meta-analysis of prospective cohort studies	• Systematic review and meta-analysis<br>• Search conducted from inception to August 1st, 2020	Aimed to assess the associations between total and specific dairy consumption and the risk of hypertension, CHD, and stroke as well as to evaluate the quality of evidence supporting the investigated relations using NutriGrade.	• Total dairy<br>• Milk<br>• Yogurt<br>• Cheese	• Total CVD<br>• Stroke CHD<br>• CVD risk factors	Healthy	Newcastle Ottawa Scale, NutriGrade
Feng, et al. (2022) [6]	Consumption of Dairy Products and the Risk of	• Meta-analysis and systematic review	To examine the associations between dairy product consumption and the risk of	• Total dairy<br>• Low-fat dairy	• Overweight or obesity<br>• Hypertension	General adult population aged ≥18 years	NUtrition QUality Evaluation

	Overweight or Obesity, Hypertension, and Type 2 Diabetes Mellitus: A Dose-Response Meta-Analysis and Systematic Review of Cohort Studies	• Comprehensive search conducted using PubMed, Embase, and Web of Science up to April 2021	overweight or obesity, hypertension, and type 2 diabetes mellitus (T2DM), and to test for dose–response relations.	• High-fat dairy<br>• Fermented dairy<br>• Milk<br>• Yogurt<br>• Cheese	• T2DM		Strengthening Tools (NUQUEST)
Gholami, et al. (2017) [8]	The effect of dairy consumption on the prevention of cardiovascular diseases: A meta-analysis of prospective studies	• Meta-analysis<br>• Search conducted from inception to September 2014<br>• N=27	To evaluate the effect of the consumption of dairy products on cardiovascular diseases, including stroke and CHD.	• Total dairy	• Total CVD<br>• Stroke<br>• CHD	Healthy	STROBE checklist
Guo, et al. (2017) [9]	Milk and dairy consumption and risk of cardiovascular diseases and all-cause mortality: dose-response meta-analysis of prospective cohort studies	• Dose-response meta-analysis<br>• Search conducted from inception to September 2016<br>• N=29	To examine linear and non-linear associations between milk and dairy products with all-cause mortality, CHD, and CVD events using existing prospective cohort studies of adequate quality.	• Total dairy<br>• Milk<br>• Fermented dairy<br>• Cheese<br>• Yogurt	• Total CVD<br>• CHD	18 years and above; no prior CVDs	Newcastle Ottawa Scale
Heidari, et al. (2021) [10]	Dairy products consumption and the risk of hypertension in	• Systematic review and dose-response meta-analysis	To conduct an updated meta-analysis, summarizing the relationship between dairy products and the risk	• Total dairy<br>• Milk	• CVD risk factors	Adults	Newcastle Ottawa Scale, AMSTAR 2, and GRADE

	adults: An updated systematic review and dose-response meta-analysis of prospective cohort studies	• Search conducted from inception to October 2020 and updated December 2020<br>• N=16	of hypertension to better inform public health recommendations and determine any possible threshold effects or dose-response relationships.	• Fermented dairy			
Jakobsen, et al. (2021) [11]	Intake of dairy products and associations with major atherosclerotic cardiovascular diseases: a systematic review and meta-analysis of cohort studies	• Systematic review and meta-analysis<br>• Search conducted from inception to August 2019<br>• N=33	To summarize the findings on the association between total intake of dairy products and intake of dairy product subgroups (milk, yogurt, cheese, butter) and the risk of major atherosclerotic CVDs (total CHD, total ischemic stroke, and peripheral artery disease) in the general adult population.	• Total dairy<br>• Milk<br>• Yogurt<br>• Cheese<br>• Butter	• Stroke<br>• CHD	Adults	Newcastle Ottawa Scale and NutriGrade
Kazemi, et al. (2023) [12]	The relationship between major food sources of fructose and cardiovascular disease, cancer, and all-cause mortality: a systematic review and dose-response meta-analysis of cohort studies	• Systematic review and dose-response meta-analysis<br>• Search strategy: Comprehensive search conducted using PubMed, Scopus, and Web of Science up to November 2020	To summarize the associations between food sources of fructose and the risk of CVD, cancer, and all-cause mortality.	• Yogurt	• CVD<br>• Cancer<br>• All-cause mortality	General adult population aged ≥18 years	Newcastle–Ottawa scale

Mazidi, et al. (2019) [13]	Consumption of dairy product and its association with total and cause specific mortality - A population-based cohort study and meta-analysis	• Population-based cohort study and meta-analysis<br>• Search conducted from inception to December 31st, 2017<br>• N=13	To examine whether consumption of total dairy and dairy subgroups was associated with total and cause specific (CHD, cerebrovascular and cancer) mortality.	• Total dairy<br>• Milk<br>• Cheese<br>• Yogurt	• CVD mortality	20 years and above; healthy	Newcastle Ottawa Scale
Mishali, et al. (2019) [14]	Association between dairy intake and the risk of contracting type 2 diabetes and cardiovascular diseases: a systematic review and meta-analysis with subgroup analysis of men versus women	• Systematic review and meta-analysis<br>• Search conducted from2006 to November 2016<br>• N=16	To examine whether the effects of dairy consumption on T2D and CVD are different in men vs women.	• Total dairy	• Total CVD	18 years and above	N/A
Mullie, et al. (2016) [15]	Daily milk consumption and all-cause mortality, coronary heart disease and stroke: a systematic review and meta-analysis of	• Systematic review<br>• Search conducted from inception to June 30th, 2015<br>• N=21	To clarify the reasons underlying the contradicting results on milk intake and all-cause mortality, CHD, and stroke.	• Milk	• Stroke<br>• CHD	Not specified	N/A

	observational cohort studies						
Naghshi, et al. (2022) [16]	High vs. low-fat dairy and milk differently affects the risk of all-cause, CVD, and cancer death: a systematic review and dose-response meta-analysis of prospective cohort studies	• Systematic review and dose-response meta-analysis<br>• Search conducted from inception to February 2020<br>• N=47	To examine the association between total, low-fat, and high-fat dairy consumption and risk of all-cause, CVD, and cancer mortality.	• Total dairy	• CVD mortality	Healthy	Risk of bias in non-randomized studies of exposure (ROBINS-E) tool
Pimpin, et al. (2016) [17]	Is butter back? A systematic review and meta-analysis of butter consumption and risk of cardiovascular disease, diabetes, and total mortality	• Systematic review and meta-analysis<br>• Search conducted from inception to May 2015<br>• N=9	To systematically review and meta-analyze the association of butter consumption with all-cause mortality, cardiovascular disease, and diabetes in general populations.	• Butter	• Total CVD<br>• Stroke<br>• CHD	18 years and above	Newcastle Ottawa Scale
Qin, et al. (2015) [18]	Dairy consumption and risk of cardiovascular disease: an updated meta-analysis of prospective cohort studies	• Meta-analysis<br>• Search conducted from inception to February 2014<br>• N=22	To examine the association of dairy consumption and its specific subtypes with CVD risk, including the risk of stroke and CHD by a meta-analysis.	• Total dairy<br>• Cheese	• Total CVD<br>• Stroke<br>• CHD	18 years and above	Author defined quality criteria checklist

Ralston, et al. (2012) [19]	A systematic review and meta-analysis of elevated blood pressure and consumption of dairy foods	• Systematic review and meta-analysis<br>• Search conducted from January 2002 to April 2009 with re-run in November 2009 from inception onwards<br>• N=5	To examine the association between dairy food intake during adulthood and the development of elevated blood pressure, specifically comparing the association of elevated blood pressure with consumption of low-fat dairy foods versus high-fat dairy foods, as well as cheese versus fluid dairy foods (milk or yogurt).	• Total dairy<br>• Cheese	• CVD risk factors	Adults	Author defined quality criteria checklist
Soedamah-Muthu, et al. (2011) [20]	Milk and dairy consumption and incidence of cardiovascular diseases and all-cause mortality: dose-responses meta-analysis of prospective cohort studies	• Meta-analysis<br>• Search conducted from February 10th, 2009, to June 1st, 2009<br>• N=17	To examine the associations of milk, total dairy products, and high- and low-fat dairy intakes with the risk of CVD [including CHD and stroke] and total mortality.	• Milk	• Total CVD<br>• Stroke<br>• CHD	18 years and above; healthy	N/A
Sun, et al. (2023) [22]	The Relationship Between Major Food Sources of Fructose and Cardiovascular Outcomes: A Systematic Review and Dose-Response Meta-Analysis of Prospective Studies	• Systematic review and dose-response meta-analysis<br>• Comprehensive search conducted using PubMed, Embase, and the Cochrane Library from inception to February 10, 2022	To examine potential dose-response relationships between major dietary sources of fructose and CVD, CHD, and stroke morbidity and mortality.	• Yogurt	• CVD morbidity and mortality<br>• CHD morbidity and mortality<br>• Stroke morbidity and mortality	General adult population	Newcastle–Ottawa scale

Tutunchi, et al. (2023) [24]	Yogurt consumption and risk of mortality from all causes, CVD and cancer: a comprehensive systematic review and dose-response meta-analysis of cohort studies	• Systematic review and dose-response meta-analysis<br>• Comprehensive search conducted using PubMed/Medline, ISI Web of Science, and Scopus databases through August 2022	To quantify the dose–response relation between yogurt consumption and risk of mortality from all causes, cardiovascular disease (CVD), and cancer.	• Yogurt	• All-cause mortality<br>• CVD mortality<br>• Cancer mortality	General adult population aged ≥18 years	Newcastle–Ottawa scale
Wu, et al. (2017) [25]	Consumption of yogurt and the incident risk of cardiovascular disease: a meta-analysis of nine cohort studies	• Systematic review and meta-analysis<br>• Search conducted from inception to January 10th, 2017<br>• N=9	To pool the evidence from prospective cohort studies on the relationship of yogurt intake and the incident risk of CVD and attempted to evaluate the potential dose-response pattern of the association.	• Yogurt	• Total CVD<br>• Stroke<br>• CHD	Not specified	Newcastle Ottawa Scale
Zhang, et al. (2020) [26]	Fermented Dairy Foods Intake and Risk of Cardiovascular Diseases: A Meta-analysis of Cohort Studies	• Systematic review and meta-analysis of prospective cohort studies<br>• Searched PubMed and CNKI databases for studies published between 1980 and 2018.	To estimate the overall effect of fermented dairy food intake (e.g., cheese and yogurt) on the risk of CVD, using data from cohort studies due to previously inconsistent findings.	• Fermented dairy<br>• Cheese<br>• Yogurt	• CVD risk<br>• Myocardial infarction<br>• CHD<br>• Stroke<br>• Unspecified/ uncategorized CVD events	Not specified	N/A
**Bone health outcomes**							

Hidayat, et al. (2020) [28]	Systematic review and meta-analysis of the association between dairy consumption and the risk of hip fracture: critical interpretation of the currently available evidence	• Systematic review and meta-analysis of prospective cohort studies<br>• The PubMed and Web of Science databases were searched for relevant articles published in any language from their inception to October 31, 2019.	To critically evaluate the association, or lack thereof, between dairy consumption (milk, yogurt, and cheese) and the risk of hip fracture, with a special focus on disentangling the controversy surrounding milk.	• Milk<br>• Yogurt<br>• Cheese	• Hip Fracture	General adult population	Newcastle Ottawa Scale
Hidayat, et al. (2022) [29]	The effects of milk supplementation on bone health indices in adults: a meta-analysis of randomized controlled trials	• Meta-analysis<br>• Search conducted from inception to May 2016<br>• N=7	To clarify the effects of milk supplementation on BMD, bone turnover markers, and hormonal indices related to bone metabolism PTH, 25(OH)D, and IGF-1 in adults.	• Milk	• BMC & BMD<br>• Biochemical markers of bone turnover	Adults	Cochranes risk of bias tool
Ma, et al. (2013) [30]	Milk intake increases bone mineral content through inhibiting bone resorption: Meta-analysis of randomized controlled trials	• Meta-analysis<br>• Search conducted from inception to November 2011<br>• N=11	To clarify the effects of milk and calcium fortified milk intake on bone health and metabolism through analysis of RCTs related to the effects of milk on bone mass and bone turnover markers.	• Milk	• Biochemical markers of bone turnover	Adults	Jadad Scores
Malmir, et al. (2020) [31]	Consumption of milk and dairy products and risk	• Systematic review and meta-analysis	To comprehensively review previous publications about milk and total dairy intake	• Total dairy<br>• Milk	• Osteoporosis<br>• Fracture	18 years and above	Newcastle Ottawa Scale

	of osteoporosis and hip fracture: a systematic review and meta-analysis	• Search conducted from inception to August 2018<br>• N=34	in relation to the risk of osteoporosis and hip fracture and to summarize earlier findings through a meta-analysis in adults.				
Matia-Martin, et al. (2019) [32]	Effects of milk and dairy products on the prevention of osteoporosis and osteoporotic fractures in Europeans and Non-Hispanic Whites from North America: A systematic review and updated meta-analysis	• Systematic review and meta-analysis<br>• Search conducted from January 1st, 2000, to April 20th, 2018<br>• N=13	To update the latest evidence regarding dairy product consumption in adults and osteoporotic fracture risk, including recent epidemiological data not assessed in previous analyses and covering the most prevalent sites of osteoporotic fracture- hip, vertebrae, and wrist.	• Total dairy<br>• Milk<br>• Cheese<br>• Yogurt	• Fracture	18 years and above; non-Hispanic	Newcastle Ottawa Scale
Shi, et al. (2020) [33]	Effects of dairy products on bone mineral density in healthy postmenopausal women: a systematic review and meta-analysis of randomized controlled trials	• Meta-analysis<br>• Search conducted from inception to November 28th, 2019<br>• N=6	To systematically collect all relevant published studies to conduct a meta-analysis to evaluate the effects of dairy product consumption on BMD and provide advice on bone health for postmenopausal women.	• Total dairy	• BMD & BMC	Women, no history of osteoporosis fracture, or chronic disease; not consuming medication impacting bone metabolism	Cochrane risk of bias tool

CVD: Cardiovascular Disease; CHD: Coronary Heart Disease; T2DM: Type 2 Diabetes Mellitus; BMD: Bone Mineral Density; PTH: Parathyroid Hormone; 25(OH)D: 25-hydroxyvitamin D; IGF-1: Insulin-like Growth Factor 1.

Supplementary Table S7. List of studies excluded from the umbrella review and reasons for exclusion

**Author, year**	**Title**	**Reason(s) for exclusion**
Benatar, et al. (2013) [43]	Effects of High and Low Fat Dairy Food on Cardio-Metabolic Risk Factors: A Meta-Analysis of Randomized Studies	Excluded due to study design. Only meta-analyses of prospective cohort studies were eligible for CVD outcomes
Companys, et al. (2020) [44]	Fermented Dairy Products, Probiotic Supplementation, and Cardiometabolic Diseases: A Systematic Review and Meta-analysis	Excluded because fermented milk and probiotic supplementation were not the primary focus of the umbrella review
Dixon, et al. (2020) [45]	Efficacy of Probiotics in Patients of Cardiovascular Disease Risk: a Systematic Review and Meta-analysis	Excluded because the focus was on probiotics rather than dairy products
Dong, et al. (2013) [46]	Effect of probiotic fermented milk on blood pressure: A meta-analysis of randomised controlled trials	Excluded because the study focused on probiotic fermented milk and included only randomized controlled trials
Fabiani, et al. (2019) [47]	Dietary Patterns in Relation to Low Bone Mineral Density and Fracture Risk: A Systematic Review and Meta-Analysis	Excluded because the focus was on dietary patterns rather than specifically on dairy consumption
Fontecha, et al. (2019) [48]	Milk and Dairy Product Consumption and Cardiovascular Diseases: An Overview of Systematic Reviews and Meta-Analyses	Excluded because this study is an overview of existing systematic reviews and meta-analyses, rather than a primary meta-analysis of individual studies
Hidayat, et al. (2017) [49]	Effects of milk proteins on blood pressure: a meta-analysis of randomized control trials	Excluded because the study focused on milk proteins and included only randomized controlled trials
Kanis, et al. (2005) [50]	A meta-analysis of milk intake and fracture risk: Low utility for case finding	Excluded because the study is a pooled analysis rather than a true meta-analysis
Larsson, et al. (2015) [51]	Milk Consumption and Mortality from All Causes, Cardiovascular Disease, and Cancer: A Systematic Review and Meta-Analysis	Excluded because this study used a dose-response meta-analysis, whereas the umbrella review focused on comparisons between the highest and lowest levels of dairy consumption in relation to CVD outcomes
Liang, et al. (2018) [52]	Biomarkers of dairy fat intake and risk of cardiovascular disease: A systematic review and meta analysis of prospective studies	Excluded because the study focused specifically on biomarkers of dairy fat intake
Micha, et al. (2017) [53]	Etiologic effects and optimal intakes of foods and nutrients for risk of cardiovascular diseases and diabetes: Systematic reviews and meta-analyses from the Nutrition and Chronic Diseases Expert Group (NutriCoDE)	Excluded because this study focused on a broader range of foods and nutrients, not specifically on dairy consumption
Mishali, et al. (2019) [54]	Funding sources and outcomes of dairy consumption research - A meta-analysis of cohort studies: The case of type-2 diabetes and cardiovascular diseases	Excluded because the focus of the study was on funding sources and their impact on outcomes, rather than directly

		assessing the relationship between dairy consumption and CVD outcomes
Ong, et al. (2020) [55]	Fermented Milk Products and Bone Health in Postmenopausal Women: A Systematic Review of Randomized Controlled Trials, Prospective Cohorts, and Case-Control Studies	Excluded because the focus was specifically on fermented milk products
Schwingshackl, et al. (2017) [56]	Food Groups and Risk of Hypertension: A Systematic Review and Dose-Response Meta-Analysis of Prospective Studies	Excluded because this study conducted a dose-response meta-analysis, whereas the umbrella review focused on comparisons between the highest and lowest levels of dairy consumption in relation to CVD outcomes
Soedamah-Muthu, et al. (2012) [57]	Dairy consumption and incidence of hypertension: A dose-response meta-analysis of prospective cohort studies	Excluded because this study used a dose-response meta-analysis, whereas the umbrella review focused on comparisons between the highest and lowest levels of dairy consumption in relation to CVD outcomes
Soto-Mendez, et al. (2019) [58]	Role of Functional Fortified Dairy Products in Cardiometabolic Health: A Systematic Review and Meta-analyses of Randomized Clinical Trials	Excluded because the focus was on functional fortified dairy products and cardiometabolic health outcomes
Sun, et al. (2015) [59]	Effects of probiotics consumption on lowering lipids and CVD risk factors: A systematic review and meta-analysis of randomized controlled trials	Excluded because the study focused on probiotics rather than general dairy consumption
Xu, e al. (2008) [60]	Effect of milk tripeptides on blood pressure: a meta-analysis of randomized controlled trials	Excluded because the study focused specifically on milk tripeptides and blood pressure, and included only randomized controlled trials

CVD: Cardiovascular Disease.

Supplementary Table S8. The detailed assessments of methodological quality of the included systematic reviews and meta-analyses

	#1. Clearly stated review question?	#2. Appropriate inclusion criteria?	#3. Appropriate search strategy?	#4. Adequate sources used?	#5. Appropriate appraisal criteria?	#6. Appraisal by ≥2 reviewers?	#7. Error minimization in data extraction?	#8. Appropriate synthesis methods?	#9. Publication bias assessed?	#10. Supported recommendations?	#11. Appropriate future research suggestions?	Quality Class
**CVD outcomes**												
Alexander, et al. (2016) [1]	Yes	Yes	Yes	No	No	No	Yes	Yes	Yes	N/A	Yes	Moderate
Bechthold, et al. (2019) [2]	Yes	Yes	Yes	No	Yes	No	Yes	Yes	Yes	Yes	N/A	Moderate
Bhandari, et al. (2023) [3]	Yes	Yes	Yes	Yes	Yes	Yes	Yes	Yes	Yes	No	No	Moderate
Chen, et al. (2017) [4]	No	Yes	No	No	Yes	Yes	Yes	Yes	Yes	N/A	Yes	Moderate
Chen, et al. (2022) [5]	Yes	Yes	Yes	Yes	Yes	Yes	Yes	Yes	Yes	No	Yes	High
Feng, et al. (2022) [6]	Yes	Yes	Yes	Yes	Yes	Yes	Yes	Yes	Yes	No	Yes	High
Gao, et al. (2020) [7]	Yes	Yes	No	Yes	Yes	No	No	No	Yes	No	Unclear	Low

Gholami, et al. (2017) [8]	No	Yes	Yes	Yes	Yes	Yes	Yes	Yes	Yes	N/A	Yes	Moderate
Guo, et al. (2017) [9]	Yes	Yes	Yes	Yes	Yes	Yes	Yes	Yes	Yes	Yes	No	High
Heidari, et al. (2021) [10]	Yes	Yes	Yes	Yes	Yes	No	Yes	Yes	No	N/A	N/A	Moderate
Jakobsen, et al. (2021) [11]	Yes	Yes	Yes	No	Yes	No	Yes	Yes	Yes	Yes	Yes	Moderate
Kazemi, et al. (2023) [12]	Yes	Yes	Yes	Yes	Yes	Yes	Yes	Yes	Yes	No	Yes	High
Mazidi, et al. (2019) [13]	Yes	Yes	Yes	Yes	Yes	Yes	Yes	Yes	Yes	Yes	N/A	High
Mishali, et al. (2019) [14]	Yes	Yes	No	No	No	No	Yes	Yes	Yes	Yes	Yes	Moderate
Mullie, et al. (2016) [15]	Yes	Yes	Yes	Yes	Yes	No	Yes	Yes	Yes	N/A	N/A	Moderate
Naghshi, et al. (2022) [16]	Yes	Yes	Yes	Yes	Yes	No	Yes	Yes	Yes	Yes	N/A	High

Pimpin, et al. (2016) [17]	Yes	Yes	Yes	Yes	Yes	Yes	Yes	Yes	Yes	Yes	N/A	High
Qin, et al. (2015) [18]	Yes	Yes	Yes	Yes	Yes	Yes	Yes	Yes	Yes	Yes	N/A	High
Ralston, et al. (2012) [19]	Yes	Yes	Yes	Yes	Yes	Unclear	Yes	Yes	No	Yes	N/A	Moderate
Soedamah-Muthu, et al. (2011) [20]	Yes	Yes	No	Yes	No	Unclear	Yes	Yes	Yes	Yes	Yes	Moderate
Soedamah-Muthu, et al. (2018) [21]	Yes	Yes	Yes	No	No	No	Yes	Yes	No	No	Yes	Low
Sun, et al. (2023) [22]	Yes	Yes	Yes	Yes	Yes	Unclear	Yes	Yes	Yes	No	Yes	Moderate
Trieu, et al. (2021) [23]	Yes	Yes	Yes	No	No	No	Yes	Yes	No	N/A	Yes	Low
Tutunchi, et al. (2023) [24]	Yes	Yes	Yes	Yes	Yes	Yes	Yes	Yes	Yes	No	No	Moderate

Wu, et al. (2017) [25]	Yes	Yes	Yes	No	Yes	Unclear	Yes	Yes	Yes	Yes	Yes	Moderate
Zhang, et al. (2020) [26]	Yes	Yes	Yes	No	Unclear	No	Yes	Yes	Yes	Yes	Yes	Moderate
**Bone health outcomes**												
Goncerz, et al. (2022) [27]2025-08-22 5:45:00 PM	Yes	Yes	Yes	No	Yes	Unclear	No	No	No	No	No	Low
Hidayat, et al. (2020) [28]	Yes	Yes	Yes	No	Yes	Yes	Yes	Yes	Yes	Yes	Yes	High
Hidayat, et al. (2022) [29]	Yes	Yes	Yes	No	Yes	No	Yes	Yes	Yes	Yes	Yes	Moderate
Ma, et al. (2013) [30]	Yes	Yes	Yes	Yes	Yes	Yes	Yes	Yes	Yes	Yes	Yes	High
Malmir, et al. (2020) [31]	Yes	Yes	Yes	Yes	Yes	No	Yes	Yes	Yes	Yes	Yes	High

Matia-Martin, et al. (2019) [32]	Yes	Yes	Yes	Yes	Yes	Yes	Yes	Yes	Yes	Yes	Yes	High
Shi, et al. (2020) [33]	Yes	Yes	Yes	Yes	Yes	Yes	Yes	Yes	Yes	Yes	N/A	High

Supplementary Table S9. Description of meta-analyses on dairy consumption and cardiovascular outcomes included in the umbrella review

Authors, year	Outcome	Exposure	Study design	Comparison/Intake	# of studies (populations)	Cases/Total	Metric	Effect size (95% CI)	P value	I^2 (%)
Alexander, et al. (2016) [1]	CVD	Total dairy	Cohort	High vs. low intake	4 (5)	NA	SRRE	0.88 (0.75, 1.04)	NA	52.7
	CVD	Milk	Cohort	High vs. low intake	4	NA	SRRE	0·94 (0·86, 1·03)	NA	38.1
	CVD	Yogurt	Cohort	High vs. low intake	3	NA	SRRE	0.93 (0.78, 1.12)	NA	43.4
	CVD	Cheese	Cohort	High vs. low intake	3	NA	SRRE	0.89 (0.78, 1.01)	NA	13.0
	CHD	Total dairy	Cohort	High vs. low intake	7 (8)	NA	SRRE	0.91 (0.80, 1.04)	NA	52.8
	CHD	Milk	Cohort	High vs. low intake	6	NA	SRRE	1.05 (0.95, 1.16)	NA	5.0
	CHD	Yogurt	Cohort	High vs. low intake	4	NA	SRRE	1.08 (0.91, 1.28)	NA	41.7
	CHD	Cheese	Cohort	High vs. low intake	5	NA	SRRE	0·.82 (0.72, 0.93)	NA	0.0

	Stroke	Total dairy	Cohort	High vs. low intake	7 (9)	NA	SRRE	0.91 (0.83, 0.99)	NA	44.5
	Stroke	Milk	Cohort	High vs. low intake	7	NA	SRRE	0.90 (0.79, 1.02)	NA	79.6
	Stroke	Cheese	Cohort	High vs. low intake	4	NA	SRRE	0.87 (0.77, 0.99)	NA	33.5
Bechthold, et al. (2019) [2]	CHD	Total Dairy	Prospect ive	High vs. low intake	13	15,790	RR	0.99 (0.92, 1.07)	NA	59.0
	CHD	Total Dairy	Prospect ive	Per 200g/day	10	NA	RR	0.99 (0.96, 1.02)	NA	55.0
	CHD	High-fat dairy	Prospect ive	High vs. low intake	7	NA	RR	1.01 (0.96, 1.06)	NA	9.0
	CHD	Low-fat dairy	Prospect ive	High vs. low intake	7	NA	RR	0.96 (0.90, 1.03)	NA	42.0
	Stroke	Total Dairy	Prospect ive	High vs. low intake	12	16,887	RR	0.96 (0.90, 1.01)	NA	43.0
	Stroke	Total Dairy	Prospect ive	Per 200g/day	11	NA	RR	0.98 (0.96, 1.00)	NA	50.0
	Stroke	High-fat dairy	Prospect ive	High vs. low intake	8	NA	RR	0.93 0.87, 0.99	NA	32.0

	Stroke	Low-fat dairy	Prospective	High vs. low intake	8	NA	RR	0.97 0.91, 1.04	NA	39.0
Bhandari, et al. (2023) [3]	CVD	Total dairy	Cohort	High vs. low intake	4 (8)	29,990	HR	1.11 (0.92, 1.34)	0.28	93.0
	CVD	Total dairy	Cohort	Dose–response	4 (8)	29,990	RR	1.003 (0.999, 1.006)	0.13	96.7
Chen, et al. (2017) [4]	CVD	Cheese	Prospective	High vs. low intake	8	NA	RR	0.90 (0.82, 0.99)	NA	0.0
	CVD	Cheese	Prospective	Per 50 g/day	7	NA	RR	0.92 (0.83, 1.02)	NA	16.9
	CHD	Cheese	Prospective	High vs. low intake	8	NA	RR	0.86 (0.77, 0.96)	NA	14.9
	CHD	Cheese	Prospective	Per 50 g/day	8	NA	RR	0.90 (0.84, 0.95)	NA	0.0
	Stroke	Cheese	Prospective	High vs. low intake	6	NA	RR	0.90 (0.84, 0.97)	NA	0.0
	Stroke	Cheese	Prospective	Per 50 g/day	5	NA	RR	0.94 (0.84, 1.04)	NA	63.7
Chen, et al. (2022) [5]	CHD	Total dairy	Cohort	High vs. low intake	18 (24)	34,248/868,821	RR	0.96 (0.92, 1.00)	NA	46.6

	CHD	Total dairy	Cohort	Dose–response (1-serving/d increase)	(13)	15,831/291,132	RR	0.98 (0.95, 1.00)	NA	56.7
	CHD	High-fat dairy	Cohort	High vs. low intake	(8)	6546/218,139	RR	1.02 (0.95, 1.08)	NA	16.8
	CHD	High-fat dairy	Cohort	Dose–response (1-serving/d increase)	(6)	5325/171,587	RR	1.00 (0.96, 1.03)	NA	34.9
	CHD	Low-fat dairy	Cohort	High vs. low intake	(9)	9646/204,435	RR	0.95 (0.87, 1.05)	NA	58.3
	CHD	Low-fat dairy	Cohort	Dose–response (1-serving/d increase)	(8)	8599/192,365	RR	0.99 (0.95, 1.04)	NA	54.9
	CHD	Milk	Cohort	High vs. low intake	(17)	22,614/823,742	RR	1.03 (0.97, 1.08)	NA	37.0
	CHD	Milk	Cohort	Dose–response (1-serving/d increase)	(16)	10,670/313,526	RR	1.00 (1.00, 1.00)	NA	0.0
	CHD	Yogurt	Cohort	High vs. low intake	(7)	8938/497,217	RR	1.02 (0.88, 1.18)	NA	64.7
	CHD	Yogurt	Cohort	Dose–response (1-serving/d increase)	(5)	34,925/119,209	RR	1.00 (1.00, 1.00)	NA	0.0

	CHD	Cheese	Cohort	High vs. low intake	(15)	19,940/684,832	RR	0.90 (0.84, 0.97)	NA	47.1
	CHD	Cheese	Cohort	Dose–response (1-serving/d increase)	(7)	7299/139,207	RR	0.97 (0.94, 1.01)	NA	62.2
	Stroke	Total dairy	Cohort	High vs. low intake	14 (19)	27,775/552,573	RR	0.90 (0.85, 0.96)	NA	60.8
	Stroke	Total dairy	Cohort	Dose–response (1-serving/d increase)	(16)	0.96 (0.93, 0.99)	RR	0.96 (0.93, 0.99)	NA	74.7
	Stroke	High-fat dairy	Cohort	High vs. low intake	(9)	14,869/412,206	RR	0.93 (0.89, 0.98)	NA	0.0
	Stroke	High-fat dairy	Cohort	Dose–response (1-serving/d increase)	(7)	0.99 (0.97, 1.00)	RR	0.99 (0.97, 1.00)	NA	0.0
	Stroke	Low-fat dairy	Cohort	High vs. low intake	(11)	15,923/432,988	RR	0.91 (0.87, 0.96)	NA	0.0
	Stroke	Low-fat dairy	Cohort	Dose–response (1-serving/d increase)	(9)	0.97 (0.95, 1.00)	RR	0.97 (0.95, 1.00)	NA	14.0
	Stroke	Milk	Cohort	High vs. low intake	(15)	27,223/665,044	RR	0.96 (0.88, 1.04)	NA	72.1

	Stroke	Milk	Cohort	Dose–response (1-serving/d increase)	(12)	0.94 (0.89, 0.99)	RR	0.94 (0.89, 0.99)	NA	76.4
	Stroke	Yogurt	Cohort	High vs. low intake	(4)	5471/107,603	RR	1.07 (0.97, 1.18)	NA	0.0
	Stroke	Yogurt	Cohort	Dose–response (1-serving/d increase)	(3)	1.08 (0.85, 1.39)	RR	1.08 (0.85, 1.39)	NA	79.8
	Stroke	Cheese	Cohort	High vs. low intake	(10)	14,799/335,543	RR	0.94 (0.88, 1.00)	NA	23.8
	Stroke	Cheese	Cohort	Dose–response (1-serving/d increase)	(7)	0.96 (0.91, 1.01)	RR	0.96 (0.91, 1.01)	NA	52.1
	HTN	Total dairy	Cohort	High vs. low intake	16 (23)	>136,025/414,148	RR	0.91 (0.86, 0.95)	NA	73.5
	HTN	Total dairy	Cohort	Dose–response (1-serving/d increase)	(19)	>120,254/314,954	RR	0.96 (0.94, 0.97)	NA	66.5
	HTN	High-fat dairy	Cohort	High vs. low intake	(11)	>25,232/103,307	RR	0.99 (0.94, 1.05)	NA	84.2
	HTN	High-fat dairy	Cohort	Dose–response (1-serving/d increase)	(7)	>10,091/35,767	RR	0.98 (0.95, 1.00)	NA	11.8

	HTN	Low-fat dairy	Cohort	High vs. low intake	(11)	>25,232/103,307	RR	0.88 (0.80, 0.96)	NA	58.9
	HTN	Low-fat dairy	Cohort	Dose–response (1-serving/d increase)	(7)	>11,075/38,012	RR	0.95 (0.92, 0.98)	NA	57.6
	HTN	Milk	Cohort	High vs. low intake	(13)	>118,481/340,403	RR	0.94 (0.90, 0.97)	NA	77.6
	HTN	Milk	Cohort	Dose–response (1-serving/d increase)	(7)	34,524/121,507	RR	0.96 (0.90, 1.02)	NA	78.0
	HTN	Yogurt	Cohort	High vs. low intake	(10)	>102,792/263,473	RR	0.95 (0.90, 1.00)	NA	65.6
	HTN	Yogurt	Cohort	Dose–response (1-serving/d increase)	(8)	94,100/232,435	RR	0.95 (0.89, 1.02)	NA	84.7
	HTN	Cheese	Cohort	High vs. low intake	(9)	>95,066/264,514	RR	0.97 (0.94, 1.01)	NA	41.8
	HTN	Cheese	Cohort	Dose–response (1-serving/d increase)	(6)	12,702/50,502	RR	1.00 (0.96, 1.04)	NA	54.0
Feng, et al. (2022) [6]	HTN	Total dairy	Cohort	200-g/d increase	17	133,319/375,975	RR	0.95 (0.93, 0.97)	NA	65.2

	HTN	High-fat dairy	Cohort	200-g/d increase	10	NA/144,624	RR	0.94 (0.90, 0.98)	NA	46.2
	HTN	Low-fat dairy	Cohort	200-g/d increase	10	NA/144,624	RR	0.96 (0.89, 1.03)	NA	61.8
	HTN	Milk	Cohort	200-g/d increase	14	118,766/310,696	RR	0.94 (0.92, 0.97)	NA	64.1
	HTN	Yogurt	Cohort	50 g/d increase	11	103,686/265,684	RR	0.98 (0.96, 1.00)	NA	81.5
	HTN	Cheese		30 g/d increase	11	104,507/267,020	RR	0.99 (0.97, 1.01)	NA	60.4
Gholami, et al. (2017) [8]	CVD	Total dairy	Cohort	High vs. low intake	10 (12)	8,648/140,851	RR	0.90 (0.81, 0.99)	NA	55.9
	CVD incidence	Total dairy	Cohort	High vs. low intake	4	NA	RR	0.93 (0.84, 1.04)	NA	32.5
	CVD mortality	Total dairy	Cohort	High vs. low intake	6 (8)	NA	RR	0.87 (0.74, 1.03)	NA	64.6
	CHD	Total dairy	Cohort	High vs. low intake	17 (21)	11,806/47,190	RR	0.99 (0.92, 1.06)	NA	51.6
	CHD incidence	Total dairy	Cohort	High vs. low intake	8 (9)	7,787	RR	1.03 (0.88, 1.21)	NA	44.9

	CHD mortality	Total dairy	Cohort	High vs. low intake	9 (12)	4,019	RR	0.97 (0.97, 1.04)	NA	51.6
	Stroke	Total dairy	Cohort	High vs. low intake	16 (19)	29,300/765,026	RR	0.88 (0.82, 0.95)	NA	63.1
	Stroke incidence	Total dairy	Cohort	High vs. low intake	8	13,979	RR	0.96 (0.88, 1.04)	NA	49.7
	Stroke mortality	Total dairy	Cohort	High vs. low intake	8 (11)	15,321	RR	0.80 (0.76, 0.83)	NA	0.0
Guo, et al. (2017) [9]	CVD	Total dairy	Cohort	Per 200 g/d	8	5,525/76,207	RR	0.97 (0.91, 1.02)	NA	59.9
	CVD	High-fat dairy	Cohort	Per 200 g/d	7	5,408/95,242	RR	0.93 (0.84, 1.03)	NA	37.4
	CVD	Low-fat dairy	Cohort	Per 200 g/d	7	5,408/95,242	RR	0.98 (0.95, 1.01)	NA	0.0
	CVD	Milk	Cohort	244 g/d	9 (12)	21,580/249,779	RR	1.01 (0.93, 1.10)	NA	92.4
	CVD	Cheese	Cohort	10 g/d	9 (11)	15,519/234,447	RR	0.98 (0.95, 1.00)	NA	82.6
	CVD	Yogurt	Cohort	50 g/d	3	817/36,624	RR	1.03 (0.97, 1.09)	NA	0.0
	CHD	Total dairy	Cohort	Per 200 g/d	11 (12)	8,298/330,350	RR	0.99 (0.96, 1.02)	NA	38.9

	CHD	High-fat dairy	Cohort	Per 200 g/d	9	6,661/171,627	RR	0.99 (0.93, 1.05)	NA	22.9
	CHD	Low-fat dairy	Cohort	Per 200 g/d	9 (10)	6,244/262,228	RR	1.00 (0.97, 1.03)	NA	27.3
	CHD	Milk	Cohort	244 g/d	11 (12)	8,612/230,621	RR	1.01 (0.96, 1.06)	NA	45.5
	CHD	Cheese	Cohort	10 g/day	9 (10)	4,022/256,091	RR	0.99 (0.97, 1.02)	NA	40.3
	CHD	Yogurt	Cohort	50 g/d	3	1,143/98,936	RR	1.03 (0.97, 1.09)	NA	0.0
Heidari, et al. (2021) [10]	HTN	Total dairy	Cohort	High vs. low intake	15	NA	RR	0.90 (0.87, 0.94)	NA	49.4
	HTN	High-fat dairy	Cohort	High vs. low intake	8	NA	RR	0.99 (0.94, 1.06)	NA	81.2
	HTN	Low-fat dairy	Cohort	High vs. low intake	8	NA	RR	0.86 (0.77, 0.96)	NA	72.0
	HTN	Milk	Cohort	High vs. low intake	11	NA	RR	0.94 (0.90, 0.99)	NA	74.3
	HTN	Cheese	Cohort	High vs. low intake	9	NA	RR	0.97 (0.92, 1.01)	NA	53.0

	HTN	Yogurt	Cohort	High vs. low intake	7	NA	RR	0.95 (0.90, 1.01)	NA	60.1
Jakobsen, et al. (2021) [11]	CHD	Milk	Cohort	High vs. low intake	6	16,478/ 619,460	RR	1.02 (0.92, 1.13)	NA	67.0
	CHD	Milk	Cohort	Per 200 g/d increase	5 (6)	NA	RR	1.02 (1.00, 1.04)	NA	0
	CHD	High-fat milk	Cohort	High vs. low intake	6	NA	RR	1.16 (1.01, 1.33)	NA	53.0
	CHD	High-fat milk	Cohort	Per 200 g/d increase	4 (5)	NA	RR	1.08 (1.00, 1.16)	NA	0.0
	CHD	Low-fat milk	Cohort	High vs. low intake	5	NA	RR	1.05 (0.92, 1.20)	NA	59.0
	CHD	Low-fat milk	Cohort	Per 200 g/d increase	3 (4)	NA	RR	1.05 (0.96, 1.13)	NA	24.0
	CHD	Cheese	Cohort	High vs. low intake	7	14,698/554, 323	RR	0.91 (0.84, 0.99)	NA	37.0
	CHD	Cheese	Cohort	Per 20 g/d increase	6 (8)	NA	RR	0.96 (0.93, 0.98)	NA	3.0
	CHD	Yogurt	Cohort	High vs. low intake	6	14,226/552, 342	RR	0.99 (0.91, 1.08)	NA	49.0

	CHD	Yogurt	Cohort	Per 100 g/d increase	5 (7)	NA	RR	0.98 (0.93, 1.03)	NA	42.0
	CHD	Butter	Cohort	High vs. low intake	4 (6)	6,562/128,757	RR	0.99 (0.92, 1.07)	NA	0.0
	CHD	Butter	Cohort	Per 6 g/d increase	4	6,562/128,757	RR	1.00 (0.99, 1.01)	NA	0.0
	Ischemic Stroke	Milk	Cohort	High vs. low intake	3	3,691/163,128	RR	0.88 (0.79, 0.98)	NA	0.0
	Ischemic Stroke	Milk	Cohort	Per 200 g/d increase	3	NA	RR	0.96 (0.91, 1.01)	NA	16.0
	Ischemic Stroke	Cheese	Cohort	High vs. low intake	3	6,208/187,281	RR	0.89 (0.78, 1.01)	NA	37.0
	Ischemic Stroke	Cheese	Cohort	Per 20 g/d increase	2 (3)	NA	RR	0.96 (0.91, 1.01)	NA	67.0
	Ischemic Stroke	Yogurt	Cohort	High vs. low intake	3	6,208/187,281	RR	1.04 (0.95, 1.13)	NA	0.0
	Ischemic Stroke	Yogurt	Cohort	Per 100 g/d increase	2	NA	RR	0.99 (0.90, 1.10)	NA	12.0
	Ischemic Stroke	Butter	Cohort	Per 6 g/d increase	2	NA	RR	1.00 (0.99, 1.01)	NA	0.0

Kazemi, et al. (2023) [12]	CVD mortality	Yogurt	Cohort	High vs. low intake	4	9,927/211,567	HR	0.92 (0.85, 0.99)	NA	0.0
	CVD mortality	Yogurt	Cohort	Per 200 g/d increase	4	9,927/211,567	HR	0.86 (0.78, 0.95)	NA	0.0
Mazidi, et al. (2019) [13]	CHD mortality	Total dairy	Cohort	High vs. low intake	5	NA	RR	1.01 (0.98, 1.03)	NA	10.2
	CHD mortality	High-fat dairy	Cohort	High vs. low intake	4	NA	RR	1.03 (0.97, 1.07)	NA	9.3
	CHD mortality	Low-fat dairy	Cohort	High vs. low intake	3	NA	RR	0.97 (0.93, 1.01)	NA	15.3
	CHD mortality	Milk	Cohort	High vs. low intake	3	NA	RR	1.04 (1.02, 1.06)	NA	10.4
Mishali, et al. (2019) [14]	CVD	Total dairy	Cohort	High vs. low intake	13	NA/460,798	RR	0.94 (0.89, 0.99)	NA	48.8
Mullie, et al. (2016) [15]	CHD	Milk	Cohort	Per 200 mL/d increase	9	37,049/403,776	RR	1.01 (0.98, 1.05)	NA	16.0
	Stroke	Milk	Cohort	Per 200 mL/d increase	10	39,352/564,717	RR	0.91 (0.82, 1.02)	NA	92.0

Naghshi, et al. (2022) [16]	CVD mortality	Total dairy	Cohort	High vs. low intake	16	29,359/675,916	PES	0.93 (0.88, 0.98)	0.01	59.7
	CVD mortality	Total dairy	Cohort	Per 1 serving increase	13	NA	PES	0.98 (0.96, 1.00)	0.10	67.7
	CVD mortality	High-fat dairy	Cohort	High vs. low intake	5	6,029/86,288	PES	0.92 (0.78, 1.10)	0.36	59.5
	CVD mortality	High-fat dairy	Cohort	Per 1 serving increase	6	NA	PES	0.96 (0.85, 1.09)	0.53	71.1
	CVD mortality	Low-fat dairy	Cohort	High vs. low intake	6	NA	PES	0.94 (0.83, 1.06)	0.30	50.2
	CVD mortality	Low-fat dairy	Cohort	Per 1 serving increase	7	NA	PES	0.97 (0.91, 1.03)	0.25	54.0
	CVD mortality	Milk	Cohort	High vs. low intake	15	42,859/1,360,032	PES	1.01 (0.93, 1.10)	0.82	93.2
	CVD mortality	Milk	Cohort	Per 1 serving increase	9	NA	PES	1.02 (0.98, 1.06)	0.40	89.1
	CVD mortality	High-fat milk	Cohort	High vs. low intake	5	17,327/324,844	PES	1.00 (0.92, 1.09)	0.97	47.3

	CVD mortality	High-fat milk	Cohort	Per 1 serving increase	4	NA	PES	1.01 (0.97, 1.04)	0.70	69.4
	CVD mortality	Low-fat milk	Cohort	High vs. low intake	4	17,163/319,412	PES	1.09 (1.02, 1.16)	0.008	4.5
	CVD mortality	Low-fat milk	Cohort	Per 1 serving increase	4	NA	PES	1.06 (0.95, 1.18)	0.29	65.3
Pimpin, et al. (2016) [17]	CVD (Total)	Butter	Cohort	Per 14 g/d increase	4	9,783/ 175,612	RR	1.00 (0.98, 1.02)	NA	0.0
	CVD	Butter	Cohort	Per 14 g/d increase	2	6,051/147,297	RR	0.99 (0.96, 1.02)	NA	0.0
	CHD	Butter	Cohort	Per 14 g/d increase	3	4,484/ 149,056	RR	0.99 (0.96, 1.03)	NA	0.0
	Stroke	Butter	Cohort	Per 14 g/d increase	2	5,299/ 173,853	RR	1.01 (0.98, 1.03)	NA	0.0
Qin, et al. (2015) [18]	CVD	Total dairy	Cohort	High vs. low intake	7 (9)	7,641/91,057	RR	0.88 (0.81, 0.96)	NA	29.6
	CHD	Total dairy	Cohort	High vs. low intake	10 (12)	8,792/253,260	RR	0.94 (0.82 ,1.07)	NA	58.5
	CHD	High-fat dairy	Cohort	High vs. low intake	7	NA	RR	1.08 (0.99, 1.17)	0.99	0.0

	CHD	Low-fat dairy	Cohort	High vs. low intake	8	NA	RR	1.02 (0.92, 1.14)	0.16	33.5
	CHD	Cheese	Cohort	High vs. low intake	7	NA	RR	0.84 (0.71, 1.00)	0.18	31.8
	CHD	Yogurt	Cohort	High vs. low intake	5	NA	RR	1.06 (0.90, 1.34)	0.13	42.9
	CHD	Butter	Cohort	High vs. low intake	5	NA	RR	1.02 (0.88, 1.20)	0.2	30.7
	Stroke	Total dairy	Cohort	High vs. low intake	10 (12)	21,801/504,803	RR	0.87 (0.77, 0.99)	NA	69.8
	Stroke	High-fat dairy	Cohort	High vs. low intake	4	NA	RR	0.95 (0.83, 1.08)	0.01	72.1
	Stroke	Low-fat dairy	Cohort	High vs. low intake	6	NA	RR	0.93 (0.88, 0.99)	0.28	20.0
	Stroke	Cheese	Cohort	High vs. low intake	4	NA	RR	0.91 (0.84, 0.98)	0.46	0.0
	Stroke	Yogurt	Cohort	High vs. low intake	2	NA	RR	0.98 (0.92, 1.06)	0.59	0.0
	Stroke	Butter	Cohort	High vs. low intake	3	NA	RR	0.94 (0.84, 1.06)	0.31	12.9
Ralston, et al. (2012) [19]	HTN	Total dairy	Cohort	High vs. low intake	5	11,584/44,769	RR	0.87 (0.81, 0.94)	NA	0.0

	HTN	High-fat dairy	Cohort	High vs. low intake	4	10,896/40465	RR	1.00 (0.89, 1.11)	NA	27.0
	HTN	Low-fat dairy	Cohort	High vs. low intake	4	10.266/40465	RR	0.84 (0.74, 0.95)	NA	38.0
	HTN	Cheese	Cohort	High vs. low intake	4	10,739/38,886	RR	1.00 (0.89, 1.12)	NA	11.0
	HTN	Fluid dairy (milk and yogurt)	Cohort	High vs. low intake	4	20,323/72,079	RR	0.92 (0.87, 0.98)	NA	0.0
Soedamah-Muthu, et al. (2011) [20]	CVD	Milk	Cohort	Per 200 mL/d	4	2,283/13, 518	RR	0.94 (0.89, 0.99)	NA	0.0
	CHD	Total dairy	Cohort	Per 200 mL/d	4	3,330/274,680	RR	1.02 (0.93, 1.11)	NA	26.2
	CHD	High-fat dairy	Cohort	Per 200 mL/d	4	3,418/274,680	RR	1.04 (0.89, 1.21)	NA	0.0
	CHD	Low-fat dairy	Cohort	Per 200 mL/d	3	3,018/240,194	RR	0.93 (0.74, 1.17)	NA	55.7
	CHD	Milk	Cohort	Per 200 mL/d	6	4,391/259,162	RR	1.00 (0.96, 1.04)	NA	26.9

	Stroke	Milk	Cohort	Per 200 mL/d	6	15,554/375,381	RR	0.87 (0.72, 1.07)	NA	94.6
Sun, et al. (2023) [22]	CVD incidence	Yogurt	Cohort	High vs. low intake	5	11,585/187,201	HR	0.92 (0.84, 1.02)	NA	59.8
	CVD incidence	Yogurt	Cohort	Per 80 g/d increase	5	11,585/187,201	HR	0.98 (0.94, 1.02)	NA	54.0
	CVD mortality	Yogurt	Cohort	High vs. low intake	7	11,620/245,728	HR	0.90 (0.83, 0.98)	NA	0.0
	CVD mortality	Yogurt	Cohort	Per 80 g/d increase	7	11,620/245,728	HR	0.96 (0.93, 0.99)	NA	0.0
Tutunchi, et al. (2023) [24]	CVD mortality	Yogurt	Cohort	High vs. low intake	11	14,623/331,261	RR	0.89 (0.81, 0.98)	NA	33.2
	CVD mortality	Yogurt	Cohort	Per 1 serving/d increase	10	NA	RR	0.86 (0.77, 0.97)	NA	36.6
Wu, et al. (2017) [25]	CVD	Yogurt	Cohort	High vs. low intake	1 (2)	NA	RR	0.87 (0.77, 0.98)	NA	11.0
	CHD	Yogurt	Cohort	High vs. low intake	5 (6)	NA	RR	1.04 (0.95, 1.15)	NA	40.4
	Stroke	Yogurt	Cohort	High vs. low intake	5 (7)	NA	RR	1.02 (0.92, 1.13)	NA	48.6

Zhang, et al. (2020) [26]	CVD risk	Yogurt	Cohort	High vs. low intake	NR (10)	15,129/299, 315	OR	0.78 (0.67–0.89)	NA	NA
	CVD risk	Cheese	Cohort	High vs. low intake	NR (8)	58,305/279, 289	OR	0.87 (0.80–0.94)	NA	NA

CVD: Cardiovascular Disease; CHD: Coronary Heart Disease; HTN: Hypertension; HR: Hazard Ratio; RR: Relative Risk; OR: Odds Ratio; SRRE: Summary Relative Risk Estimates; PES: Pooled Effect Size; NA: Not Available.

Supplementary Table S10. Association between dairy consumption (highest vs. lowest intake level) and cardiovascular outcomes

Cardiovascular Outcomes	Exposure	95% prediction interval	Small-study effects (Egger's test)	Excess statistical significance	Power of largest Study	Sig. largest Study
**Total CVD**	Total Dairy	0.87- 1.06	No (p = 0.11)	No	0.99	False
	Milk[#]	0.89-1.05	Yes (p = 0.006)	No	0.16	True
	Yogurt	0.80-1.07	No (p = 0.77)	No	0.34	True
**CHD**	Total Dairy	0.89-1.08	No (p = 0.99)	No	0.53	False
	High Fat Dairy	0.94-1.09	No (p = 0.78)	No	0.35	False
	Low fat dairy	0.92-1.05	No (p = 0.41)	No	0.07	False
	Milk	0.98-1.06	No (p = 0.97)	Yes	0.05	True
	Yogurt	0.83-1.16	No (p= 0.35)	No	0.58	False
	Butter	0.94-1.04	N/A*		0.74	False
**Stroke**	Total Dairy	0.65-1.17	No (p= 0.49)	No	0.99	True
	High fat dairy	0.84-0.99	N/A*	No	0.65	False
	Low fat dairy	0.83-0.96	N/A*	No	0.79	True
	Milk	0.67-1.22	Yes (p = 0.07)	No	0.99	True
	Yogurt	0.81-1.24	N/A*	No	0.99	False
**Hypertension**	Total dairy	0.73-1.09	No (p = 0.76)	No	0.99	True
	High fat dairy	0.90-1.03	N/A*	No	0.13	False
	Low fat dairy	0.81-0.94	N/A*	No	0.70	True
	Milk	0.86-1.04	No (p = 0.69)	No	0.99	True
	Yogurt	0.72-1.30	N/A*	Yes	0.51	True

CVD: Cardiovascular Disease; CHD: Coronary Heart Disease; RR: Relative Risk; N/A: Not Applicable. *Egger's test was not applicable due to the availability of fewer than 10 studies. [#]Only the meta-analysis by Naghshi et at reported subgroup analysis for low fat and high fat milk.

Supplementary Table S11. Adjusted Relative Risk for milk and CVD and milk and Stroke using Two methods of SSE Correction

	Trim and Fill				Copas	
	K	RR (95%CI)	P value	I^2	N	RR (95%CI)
Milk and CVD	29 (With 6 added studies)	0.9843 (0.9539-1.0158)	0.3	56%	(Added studies=0)	0.97 (0.9408-1.0001)
Milk and Stroke	18 (with added 6 studies)	1.0014 (0.9067-1.1059	0.9	93.3%	Added studied=0	0.9039 (0.8346-0.9789)

Supplementary Table S12. Description of meta-analyses on dairy consumption and bone health outcomes included in the umbrella review

Authors, year	Outcome	Exposure	Study design	Comparison/ Intake	# of studies (populations)	Cases or intervention /Total	Metric	Effect size (95% CI)	P value	I^2 (%)
Hidayat, et al. (2020) [28]	Hip fracture	Milk	Cohort	High vs. low intake	9 (14)	9,564/363,383	RR	0.86 (0.73, 1.02)	NA	60.1
	Hip fracture	Milk	Cohort	Per 1 glass/d increase	7	NA	RR	0.97 (0.92, 1.03)	NA	NA
	Hip fracture	Yogurt	Cohort	High vs. low intake	4 (6)	8,217/234,654	RR	0.78 (0.68, 0.90)	NA	14.3
	Hip fracture	Cheese	Cohort	High vs. low intake	4 (8)	8,860/305,157	RR	0.85 (0.66, 1.08)	NA	76.9
Hidayat, et al. (2022) [29]	BMD (whole body)	Milk	RCT	Milk supplementation vs. placebo or habitual diet	3	313/629	WMD	0.005 (-0.006, 0.016)	NA	53.5
	BMD (Hip)	Milk	RCT	Milk supplementation vs. placebo or habitual diet	9	628/1,229	WMD	0.004 (0.002, 0.007)	NA	0

	BMD (femoral neck)	Milk	RCT	Milk supplementation vs. placebo or habitual diet	7	406/781	WMD	0.002 (-0.003, 0.007)	NA	74.7
	BMD (lumbar spine)	Milk	RCT	Milk supplementation vs. placebo or habitual diet	7	566/1,105	WMD	0.025 (0.005, 0.045)	NA	75.5
	Osteocalcin	Milk	RCT	Milk supplementation vs. placebo or habitual diet	9	529/1,050	WMD	-0.11 (-1.23, 1.00)	NA	82.3
	P1NP	Milk	RCT	Milk supplementation vs. placebo or habitual diet	9	587/1,179	WMD	-5.2 (-9.07, -1.33)	NA	64.3
	BALP	Milk	RCT	Milk supplementation vs. placebo or habitual diet	3	227/458	WMD	0.25 (-0.39, 0.89)	NA	52.4
	CTx	Milk	RCT	Milk supplementation vs. placebo or habitual diet	9	543/1,089	WMD	-0.16 (-0.23, -0.10)	NA	98.2

	NTx	Milk	RCT	Milk supplementation vs. placebo or habitual diet	3	99/200	WMD	-8.66 (-13.57, -3.75)	NA	38.2
Ma, et al. (2013) [30]	BMD (whole body)	Milk	RCT	Milk intake vs. control/placebo	6	NA/2,091	WMD	0.01 (-0.01, 0.03)	NA	NA
	BMC (total)	Milk	RCT	Milk intake vs. control/placebo	4	NA/1,511	WMD	40.32 (17.58, 63.05)	NA	NA
	Osteocal cin	Milk	RCT	Milk intake vs. control/placebo	5	NA/338	WMD	-5.9 (-7.5, -4.3)	NA	NA
	NTx	Milk	RCT	Milk intake vs. control/placebo	3	NA/154	WMD	-5.41 (-10.35, -0.47)	NA	NA
Malmir, et al. (2020) [31]	Osteopor osis	Total dairy	Cohort	High vs. low intake	3 (4)	866/5,924	RR	0.82 (0.56, 1.18)	NA	71.6
	Osteopor osis	Total dairy	CS/CC	High vs. low intake	8	3,161/12,723	RR	0.63 (0.55, 0.73)	NA	0.0
	Osteopor osis	Total dairy	CS/CC	Per 200 g/d increase	4	NA	RR	0.78 (0.69, 0.89)	NA	NA
	Osteopor osis	Milk	Cohort	High vs. low intake	1 (2)	NA	RR	1.08 (0.52, 2.24)	NA	81.6

	Osteoporosis	Milk	CS/CC	High vs. low intake	5 (6)	NA	RR	0.68 (0.50, 0.94)	NA	42.2
	Osteoporosis	Milk	CS/CC	Per 200 g/d increase	3	NA	RR	0.63 (0.49, 0.81)	NA	NA
	Osteoporosis	Milk	Cohort/ CS/CC	High vs. low intake	6 (8)	1,576/6,875	RR	0.79 (0.57, 1.08)	NA	63.3
	Osteoporosis	Milk	Cohort/ CS/CC	Per 200 g/d increase	NA	NA	RR	0.61 (0.50, 0.75)	NA	NA
	Hip fracture	Total dairy	Cohort	High vs. low intake	6 (7)	9,134/192,725	RR	0.90 (0.73, 1.11)	NA	79.8
	Hip fracture	Total dairy	Cohort	Per 200 g/d increase	5 (6)	NA	RR	0.98 (0.95, 1.01)	NA	NA
	Hip fracture	Total dairy	CS/CC	High vs. low intake	3	2,610/12,526	RR	0.86 (0.53, 1.38)	NA	69.0
	Hip fracture	Milk	Cohort	High vs. low intake	10 (14)	28,075/295,897	RR	0.93 (0.75, 1.15)	NA	86.7
	Hip fracture	Milk	Cohort	Per 200 g/d increase	8 (11)	NA	RR	1.09 (1.07, 1.11)	NA	NA
	Hip fracture	Milk	CS/CC	High vs. low intake	9	5,060/18,364	RR	0.75 (0.57, 0.99)	NA	73.2

Matia-Martin, et al. (2019) [32]	Osteoporotic fracture	Total dairy	Cohort	High vs. low intake	3	22,962/110,134	HR	0.95 (0.87, 1.03)	NA	82.9
	Osteoporotic fracture	Milk	Cohort	High vs. low intake	3	22,962/110,134	HR	1.05 (0.94, 1.18)	NA	61.3
	Osteoporotic fracture	Cheese	Cohort	High vs. low intake	2	23,077/108,254	HR	0.89 (0.81, 0.98)	NA	59.0
	Osteoporotic fracture	Yogurt	Cohort	High vs. low intake	2	23,077/108,254	HR	0.92 (0.87, 0.98)	NA	0.0
	Hip fracture	Total dairy	Cohort	High vs. low intake	5 (7)	7,295/236,136	HR	0.87 (0.75, 1.01)	NA	86.7
	Hip fracture	Total dairy	Cohort	Each increment in dairy intake	4 (6)	NA/231,442	HR	0.98 (0.95, 1.01)	NA	86.2
	Hip fracture	Milk	Cohort	High vs. low intake	5 (7)	NA/236,136	HR	0.91 (0.69, 1.21)	NA	87.9
	Hip fracture	Milk	Cohort	Each increment in dairy intake	3 (5)	NA	HR	1.01 (0.96, 1.06)	NA	83.7
	Hip fracture	Cheese	Cohort	High vs. low intake	4 (6)	7,295/205,955	HR	0.80 (0.62, 1.03)	NA	86.5

	Hip fracture	Cheese	Cohort	Each increment in dairy intake	3 (5)	NA	HR	0.96 (0.88, 1.04)	NA	90.8
	Hip fracture	Yogurt	Cohort	High vs. low intake	5 (7)	7,338/209,162	HR	0.87 (0.71, 1.05)	NA	66.8
	Hip fracture	Yogurt	Cohort	Each increment in dairy intake	3 (5)	NA	HR	0.96 (0.91, 1.01)	NA	71.7
	Vertebral fracture	Total dairy	Cohort	High vs. low intake	3 (4)	NA/11,893	HR	0.82 (0.68, 0.99)	NA	0.0
	Vertebral fracture	Milk	Cohort	High vs. low intake	3 (4)	NA/11,893	HR	0.81 (0.66 1.00)	NA	0.0
Shi, et al. (2020) [33]	BMD (whole body)	Total dairy	RCT	Dairy product supplementation vs. placebo or no intervention	3	225/433	SMD	0.58 (0.39, 0.77)	NA	54.0
	BMD (Hip)	Total dairy	RCT	Dairy product supplementation vs. placebo or no intervention	4	269/507	SMD	0.37 (0.20, 0.55)	NA	0.0
	BMD (femoral neck)	Total dairy	RCT	Dairy product supplementation vs. placebo or no intervention	5	289/547	SMD	0.36 (0.19, 0.53)	NA	7.0
	BMD (lumbar spine)	Total dairy	RCT	Dairy product supplementation	6	328/622	SMD	0.21 (0.05, 0.37)	NA	0.0

				vs. placebo or no intervention						

BMD: Bone Mineral Density; P1NP: Procollagen Type I N-Terminal Propeptide; BALP: Bone Alkaline Phosphatase; CTx: C-Terminal Telopeptide of Type I Collagen; NTx: N-Terminal Telopeptide; HR: Hazard Ratio; RR: Relative Risk; WMD: Weighted Mean Difference; SMD: Standardized Mean Difference; CS/CC: Cross-Sectional/Case Control; RCT: Randomized Controlled Trial; NA: Not Available.

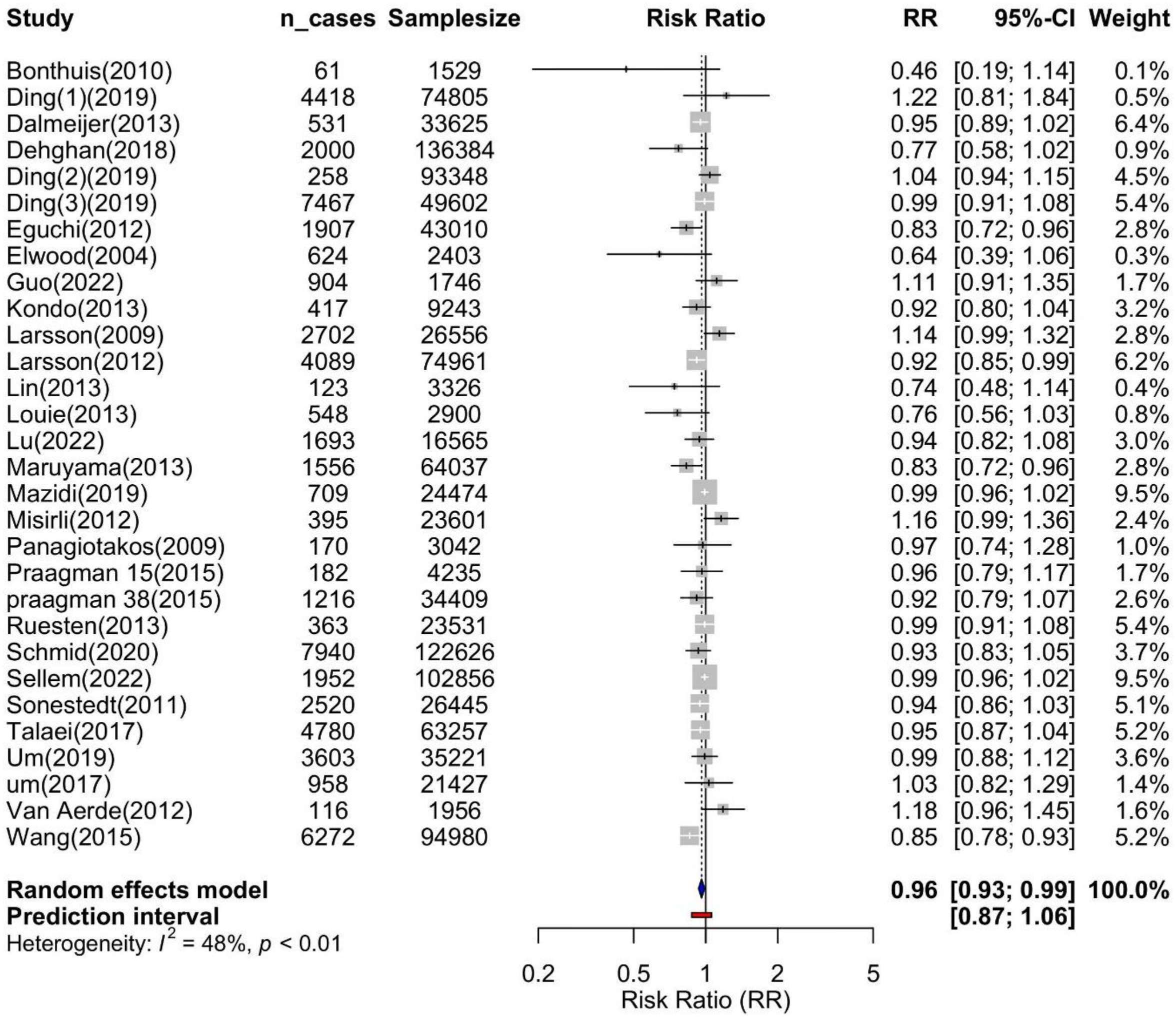

Supplementary Figure S1. Association between total dairy consumption (highest vs. lowest intake level) and CVD risk.

Study-specific effect sizes are represented by squares, with the size of each square proportional to the study's weight in the overall meta-analysis. Horizontal lines indicate 95% confidence intervals (CIs). Diamonds represent the pooled RR along with its 95% CI.

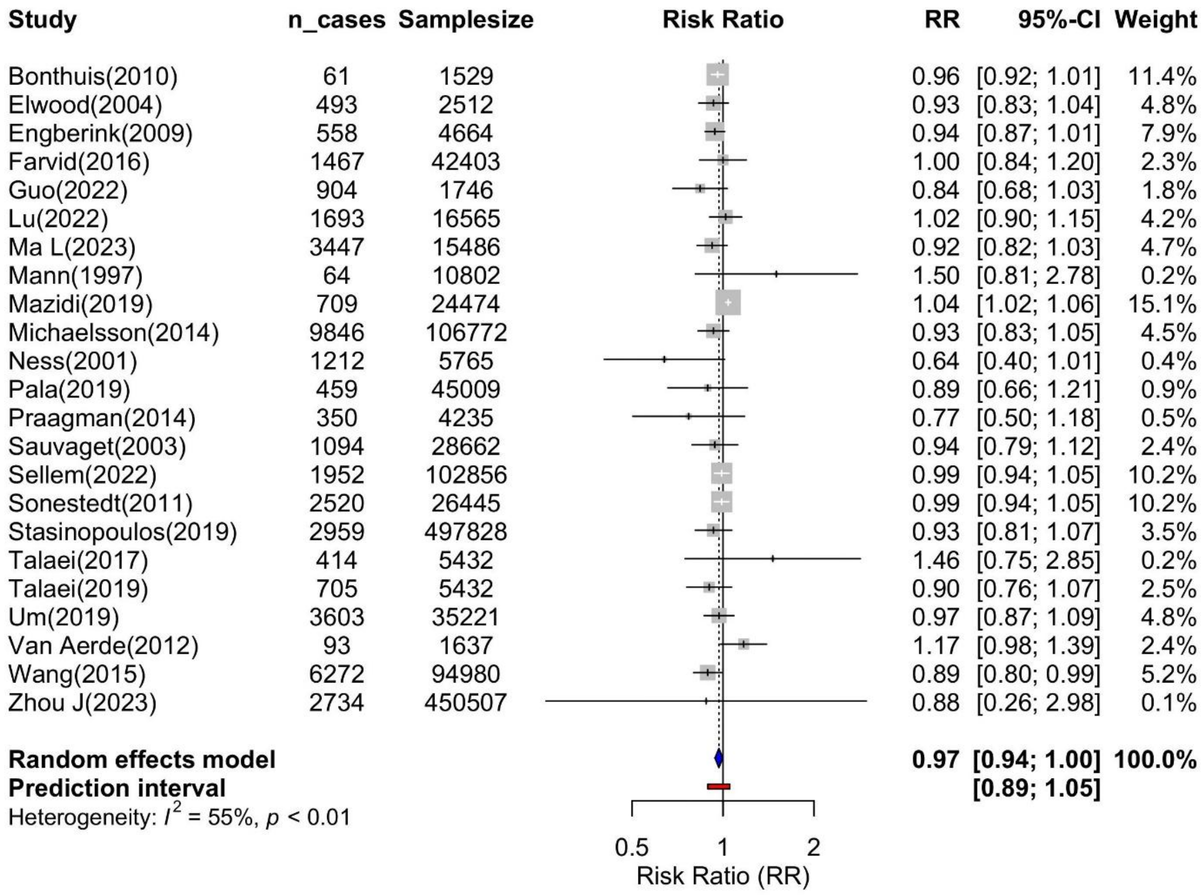

Supplementary Figure S2. Association between milk consumption (highest vs. lowest intake level) and CVD risk.

Study-specific effect sizes are represented by squares, with the size of each square proportional to the study's weight in the overall meta-analysis. Horizontal lines indicate 95% confidence intervals (CIs). Diamonds represent the pooled RR along with its 95% CI.

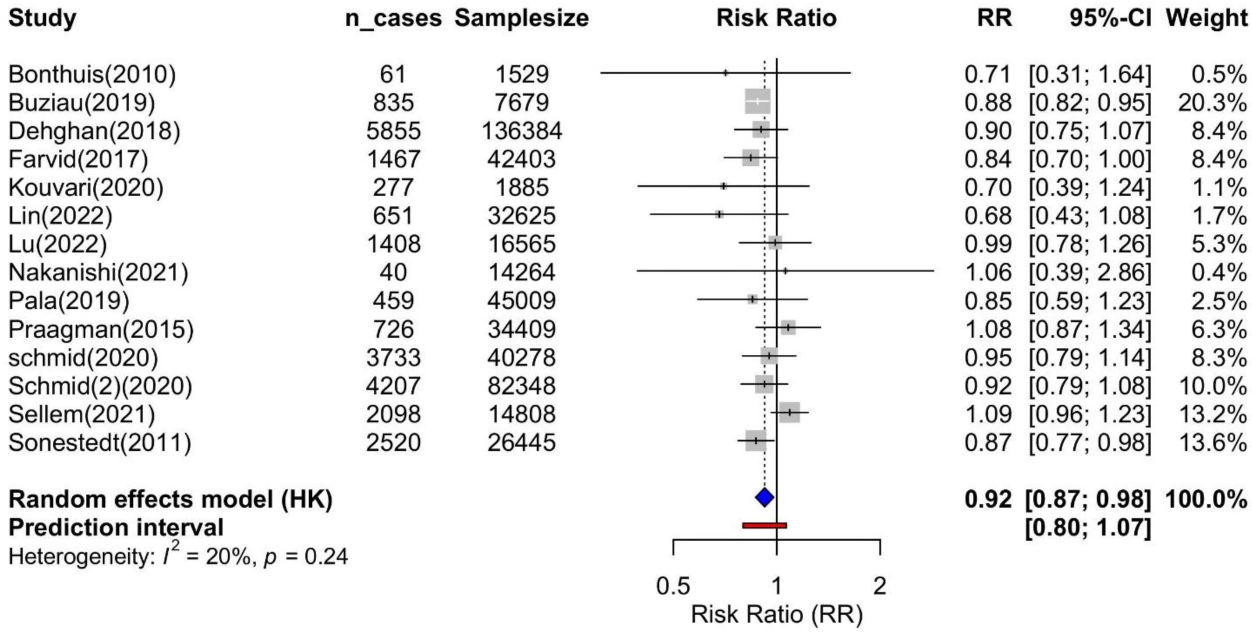

Supplementary Figure S3. Association between yogurt consumption (highest vs. lowest intake level) and CVD risk.

Study-specific effect sizes are represented by squares, with the size of each square proportional to the study's weight in the overall meta-analysis. Horizontal lines indicate 95% confidence intervals (CIs). Diamonds represent the pooled RR along with its 95% CI.

Study	n_cases	Sample size	RR	95%-CI	Weight
Maruyama(2013)	479	64037	1.03	[0.75; 1.41]	0.8%
Mazidi(2018)	709	24474	0.99	[0.96; 1.02]	9.4%
Al-Delaimy(2003)	14468	39800	0.98	[0.93; 1.03]	7.9%
Avalos(2012)	451	1759	1.00	[0.77; 1.30]	1.1%
Bergholdt(2015)	2777	70709	0.96	[0.86; 1.07]	4.1%
Bernstein(2011)	3162	84136	0.99	[0.91; 1.08]	5.4%
Bostick(1999)	387	34486	0.94	[0.66; 1.34]	0.6%
Buckland(2009)	606	40757	1.52	[1.22; 1.89]	1.5%
Dalmeijer(2013)	1648	33625	0.99	[0.94; 1.05]	7.6%
Dehghan(2018)	2594	136384	0.89	[0.71; 1.11]	1.4%
Dilis(2012)	636	23929	1.05	[0.93; 1.19]	3.5%
Elwood(2004)	493	2403	0.71	[0.40; 1.26]	0.2%
Farvid(2017)	1467	42403	0.73	[0.57; 0.93]	1.2%
Goldbohm(2011)	3234	20782	0.95	[0.86; 1.05]	4.6%
Guo(2022)	332	1746	0.76	[0.52; 1.11]	0.5%
Haring(2014)	1147	12066	1.04	[0.84; 1.29]	1.5%
Hu(1999)	939	80082	1.05	[1.01; 1.09]	8.9%
Johansson(2019)	3102	48341	0.95	[0.86; 1.05]	4.6%
Kelemen(2005)	739	29017	1.41	[1.07; 1.86]	1.0%
Key(2019)	7198	409885	0.91	[0.87; 0.96]	8.0%
Kondo(2013)	174	9243	1.49	[0.91; 2.44]	0.3%
Koskinen(2018)	472	1981	0.97	[0.71; 1.33]	0.7%
Louie(2013)	158	2662	0.71	[0.51; 0.99]	0.7%
Mann(1997)	64	10802	1.50	[0.81; 2.78]	0.2%
Martinez-Gonzalez(2011)	68	13609	1.51	[0.91; 2.50]	0.3%
Patterson(2013)	1392	33636	0.77	[0.63; 0.95]	1.6%
Praagman(2014)	567	4235	0.98	[0.77; 1.25]	1.2%
Sellem(2022)	1952	102856	0.99	[0.96; 1.02]	9.4%
Soedamah-Muthu(2013)	323	4255	0.91	[0.68; 1.22]	0.9%
Sonestedt(2011)	1344	26445	0.86	[0.73; 1.02]	2.3%
Talaei(2017)	4780	63257	0.94	[0.82; 1.07]	3.2%
Um(2019)	3603	35221	0.99	[0.88; 1.12]	3.6%
Van Aerde(2013)	50	1956	1.16	[0.92; 1.47]	1.3%
Van der Pols(2009)	378	4374	1.34	[0.75; 2.39]	0.2%
Warensjo(2010)	174	1000	1.17	[0.64; 2.13]	0.2%
**Random effects model**			**0.98**	**[0.95; 1.01]**	**100.0%**
**Prediction interval**				**[0.89; 1.08]**	

Heterogeneity: $I^2 = 56\%$, $p < 0.01$

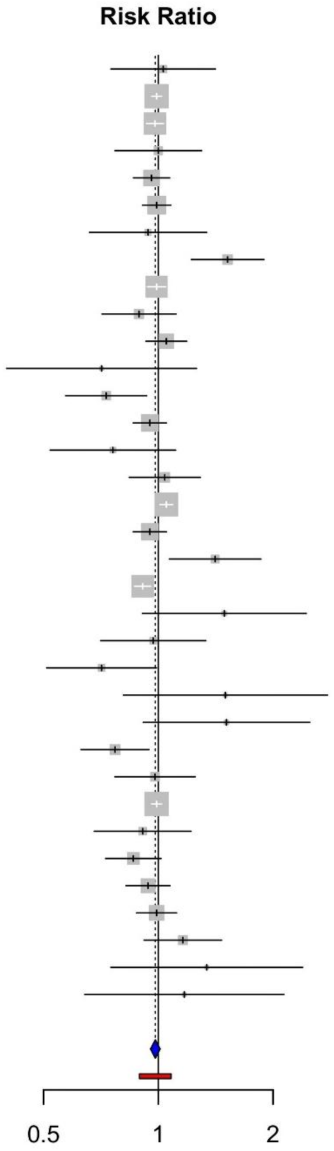

Supplementary Figure S4. Association between total dairy consumption (highest vs. lowest intake level) and CHD risk.

Study-specific effect sizes are represented by squares, with the size of each square proportional to the study's weight in the overall meta-analysis. Horizontal lines indicate 95% confidence intervals (CIs). Diamonds represent the pooled RR along with its 95% CI.

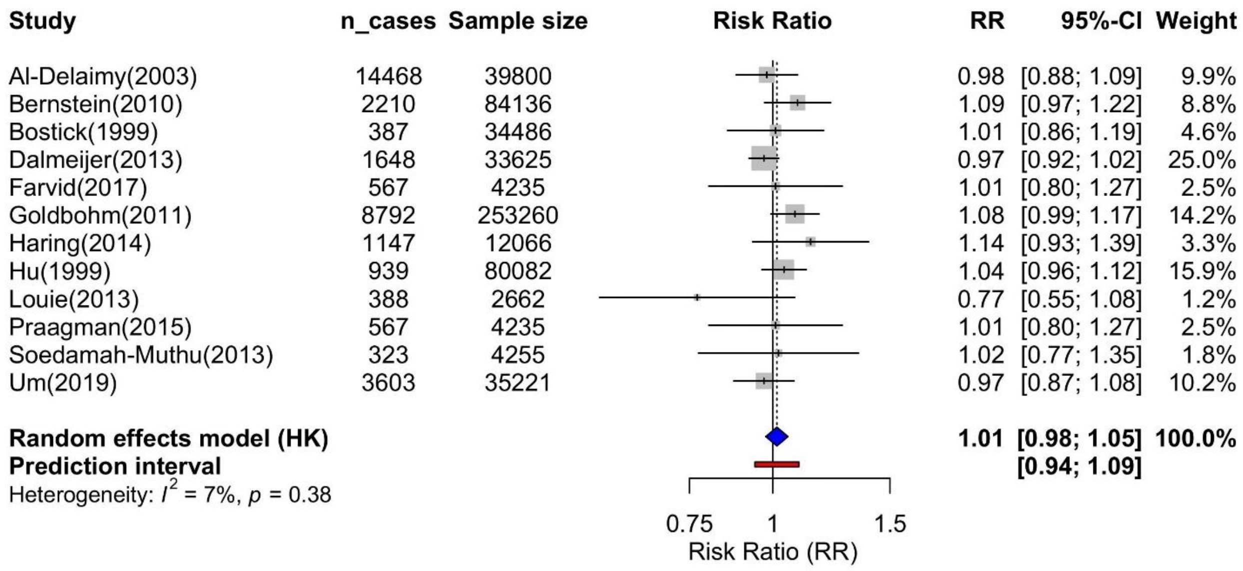

Supplementary Figure S5. Association between high-fat dairy consumption (highest vs. lowest intake level) and CHD risk.

Study-specific effect sizes are represented by squares, with the size of each square proportional to the study's weight in the overall meta-analysis. Horizontal lines indicate 95% confidence intervals (CIs). Diamonds represent the pooled RR along with its 95% CI.

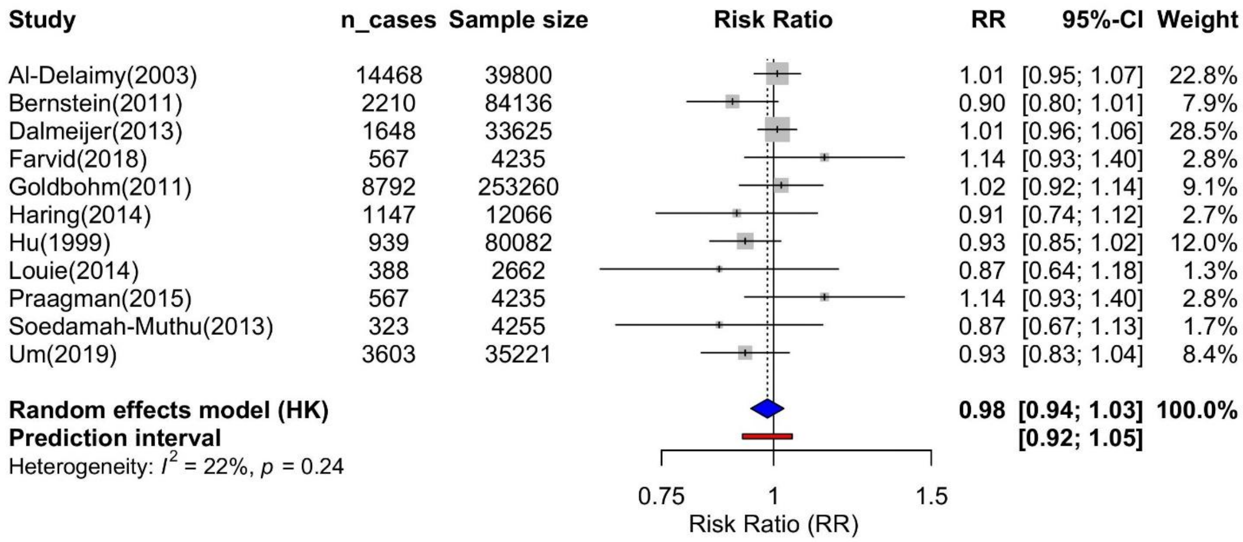

Supplementary Figure S6. Association between low-fat dairy consumption (highest vs. lowest intake level) and CHD risk.

Study-specific effect sizes are represented by squares, with the size of each square proportional to the study's weight in the overall meta-analysis. Horizontal lines indicate 95% confidence intervals (CIs). Diamonds represent the pooled RR along with its 95% CI.

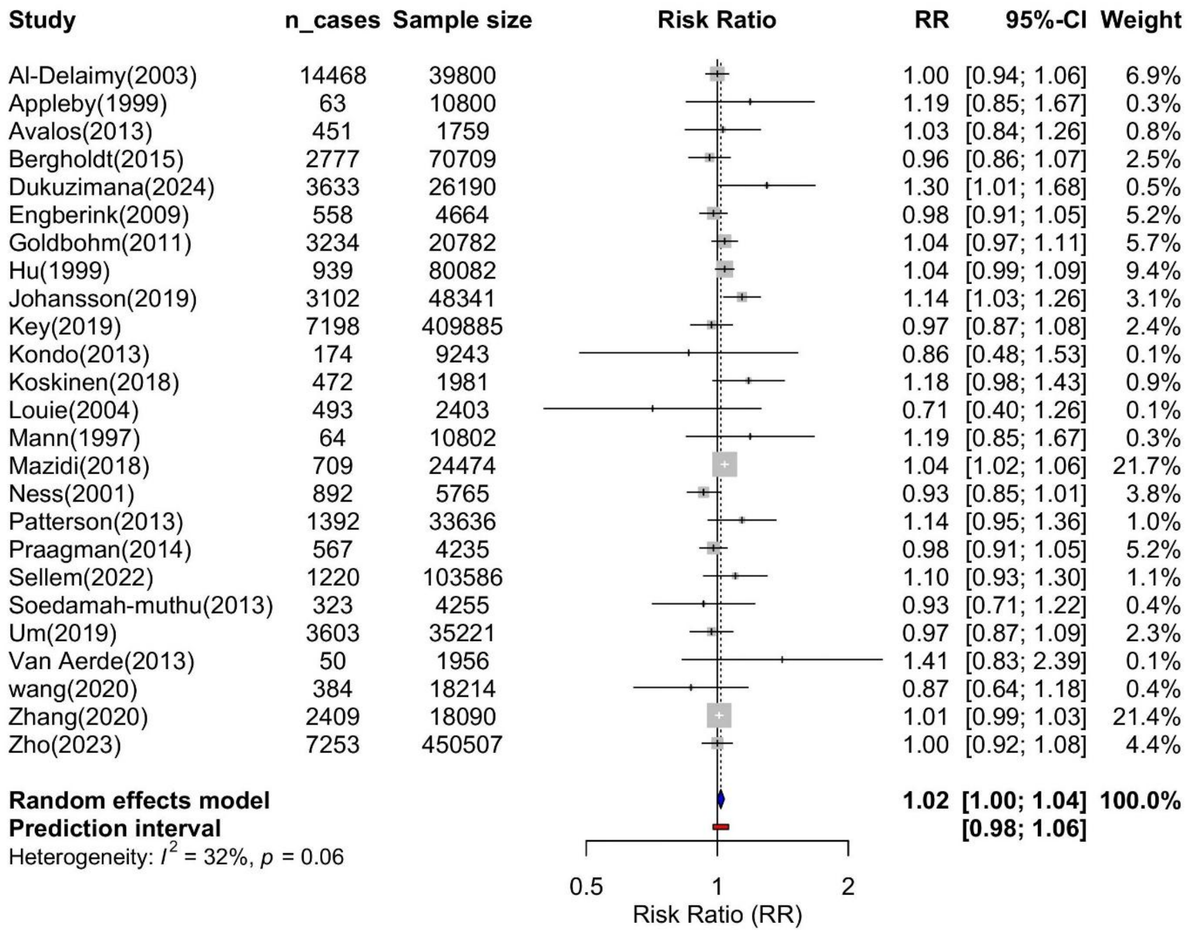

Supplementary Figure S7. Association between milk consumption (highest vs. lowest intake level) and CHD risk.

Study-specific effect sizes are represented by squares, with the size of each square proportional to the study's weight in the overall meta-analysis. Horizontal lines indicate 95% confidence intervals (CIs). Diamonds represent the pooled RR along with its 95% CI.

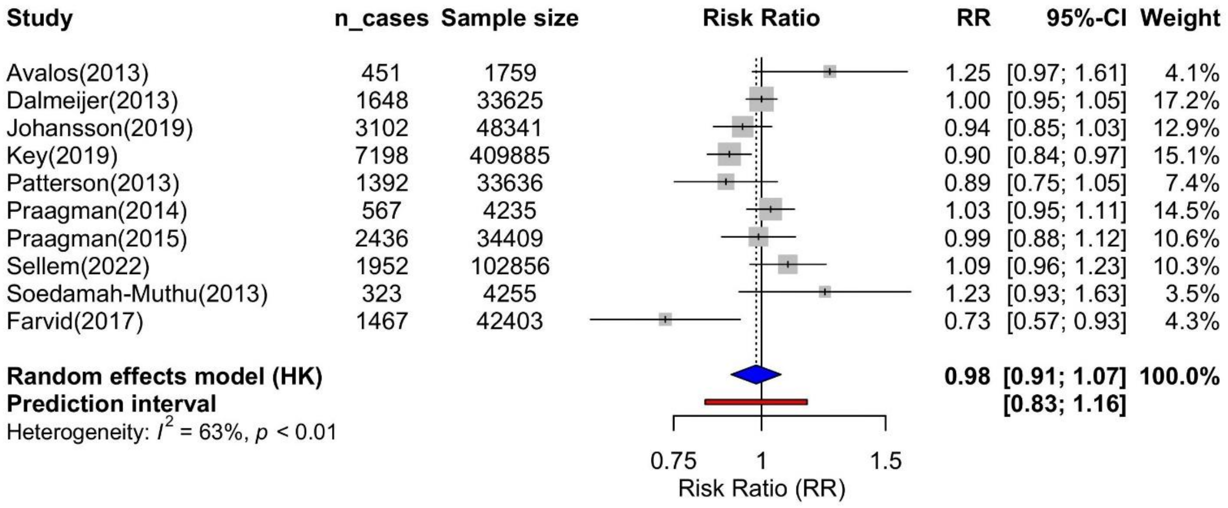

Supplementary Figure S8. Association between yogurt consumption (highest vs. lowest intake level) and CHD risk.

Study-specific effect sizes are represented by squares, with the size of each square proportional to the study's weight in the overall meta-analysis. Horizontal lines indicate 95% confidence intervals (CIs). Diamonds represent the pooled RR along with its 95% CI.

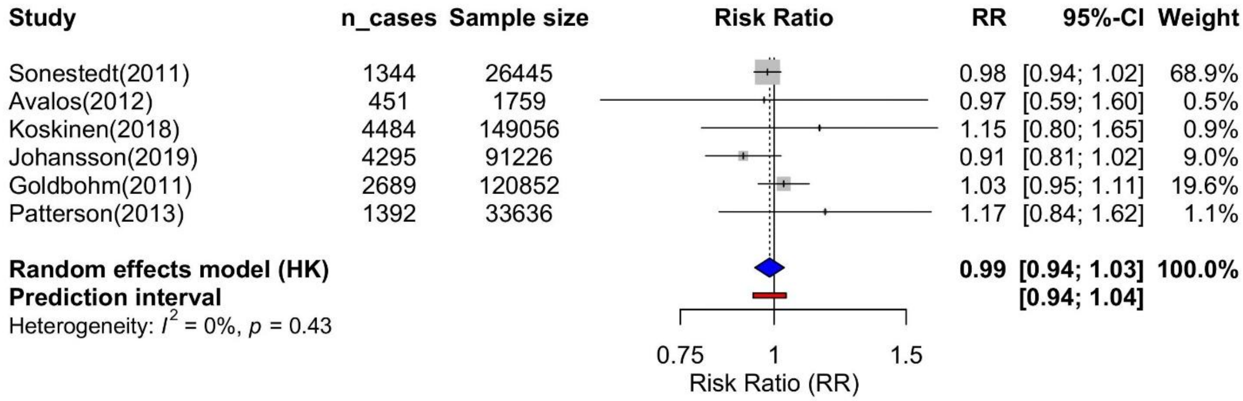

Supplementary Figure S9. Association between butter consumption (highest vs. lowest intake level) and CHD risk.

Study-specific effect sizes are represented by squares, with the size of each square proportional to the study's weight in the overall meta-analysis. Horizontal lines indicate 95% confidence intervals (CIs). Diamonds represent the pooled RR along with its 95% CI.

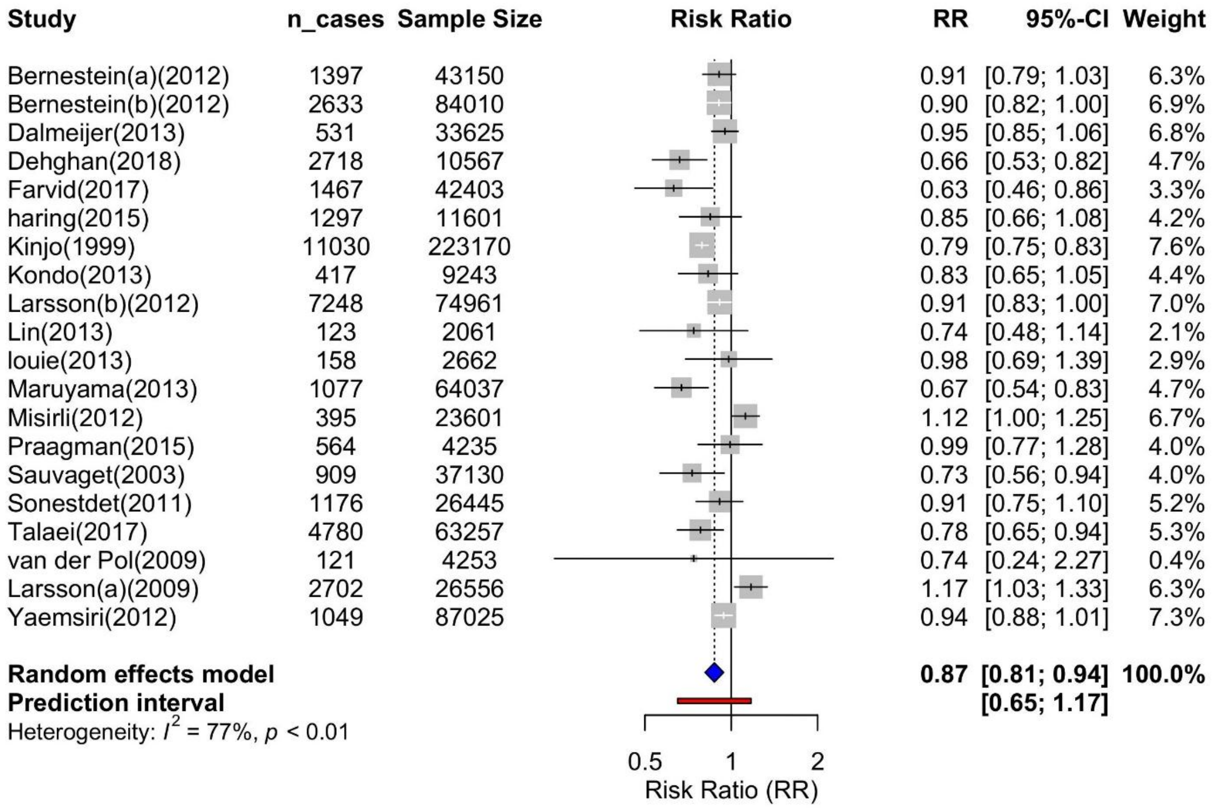

Supplementary Figure S10. Association between total dairy consumption (highest vs. lowest intake level) and stroke risk.

Study-specific effect sizes are represented by squares, with the size of each square proportional to the study's weight in the overall meta-analysis. Horizontal lines indicate 95% confidence intervals (CIs). Diamonds represent the pooled RR along with its 95% CI.

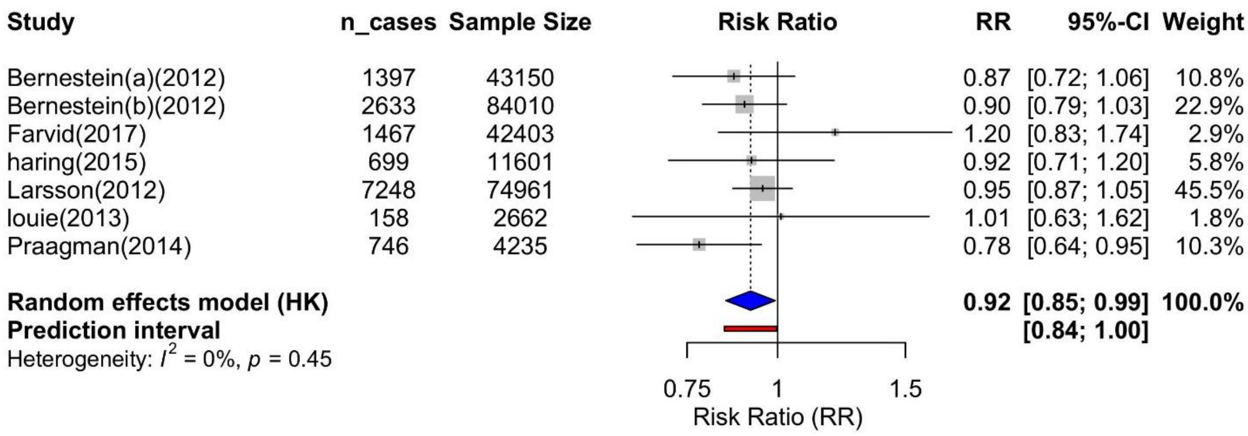

Supplementary Figure S11. Association between high-fat dairy consumption (highest vs. lowest intake level) and stroke risk.

Study-specific effect sizes are represented by squares, with the size of each square proportional to the study's weight in the overall meta-analysis. Horizontal lines indicate 95% confidence intervals (CIs). Diamonds represent the pooled RR along with its 95% CI.

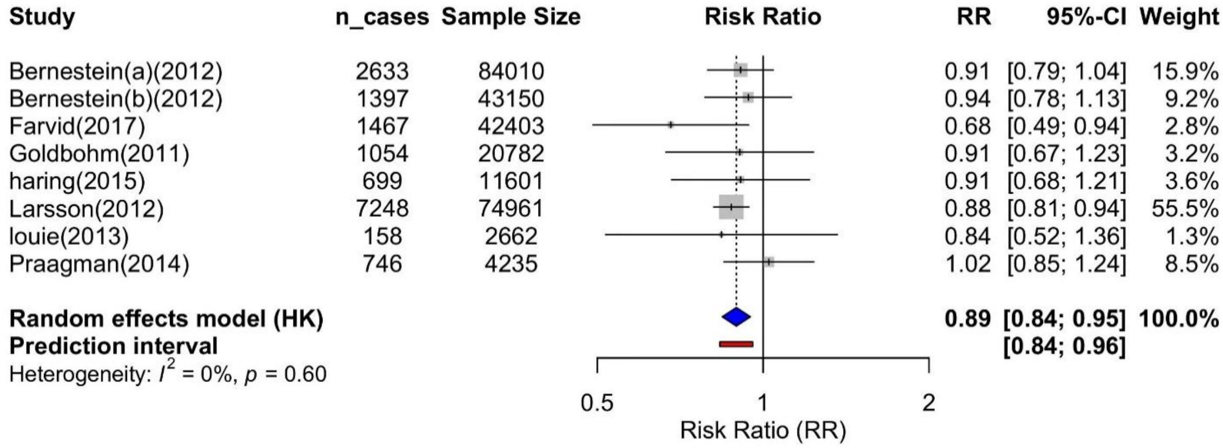

Supplementary Figure S12. Association between low-fat dairy consumption (highest vs. lowest intake level) and stroke risk.

Study-specific effect sizes are represented by squares, with the size of each square proportional to the study's weight in the overall meta-analysis. Horizontal lines indicate 95% confidence intervals (CIs). Diamonds represent the pooled RR along with its 95% CI.

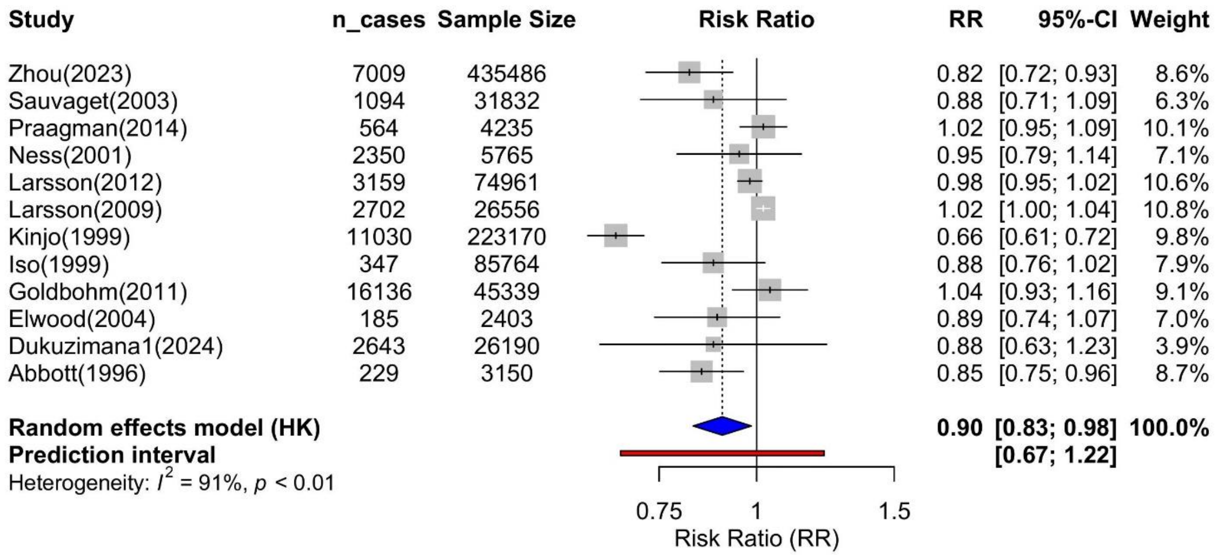

Supplementary Figure S13. Association between milk consumption (highest vs. lowest intake level) and stroke risk.

Study-specific effect sizes are represented by squares, with the size of each square proportional to the study's weight in the overall meta-analysis. Horizontal lines indicate 95% confidence intervals (CIs). Diamonds represent the pooled RR along with its 95% CI.

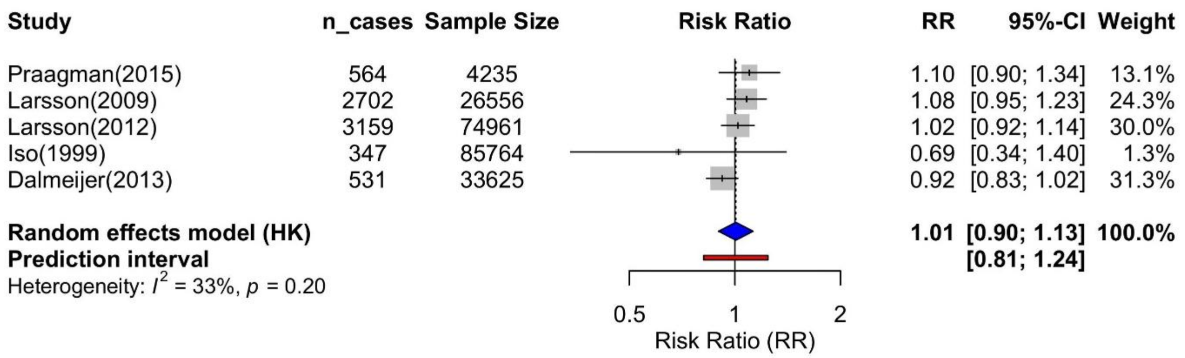

Supplementary Figure S14. Association between yogurt consumption (highest vs. lowest intake level) and stroke risk.

Study-specific effect sizes are represented by squares, with the size of each square proportional to the study's weight in the overall meta-analysis. Horizontal lines indicate 95% confidence intervals (CIs). Diamonds represent the pooled RR along with its 95% CI.

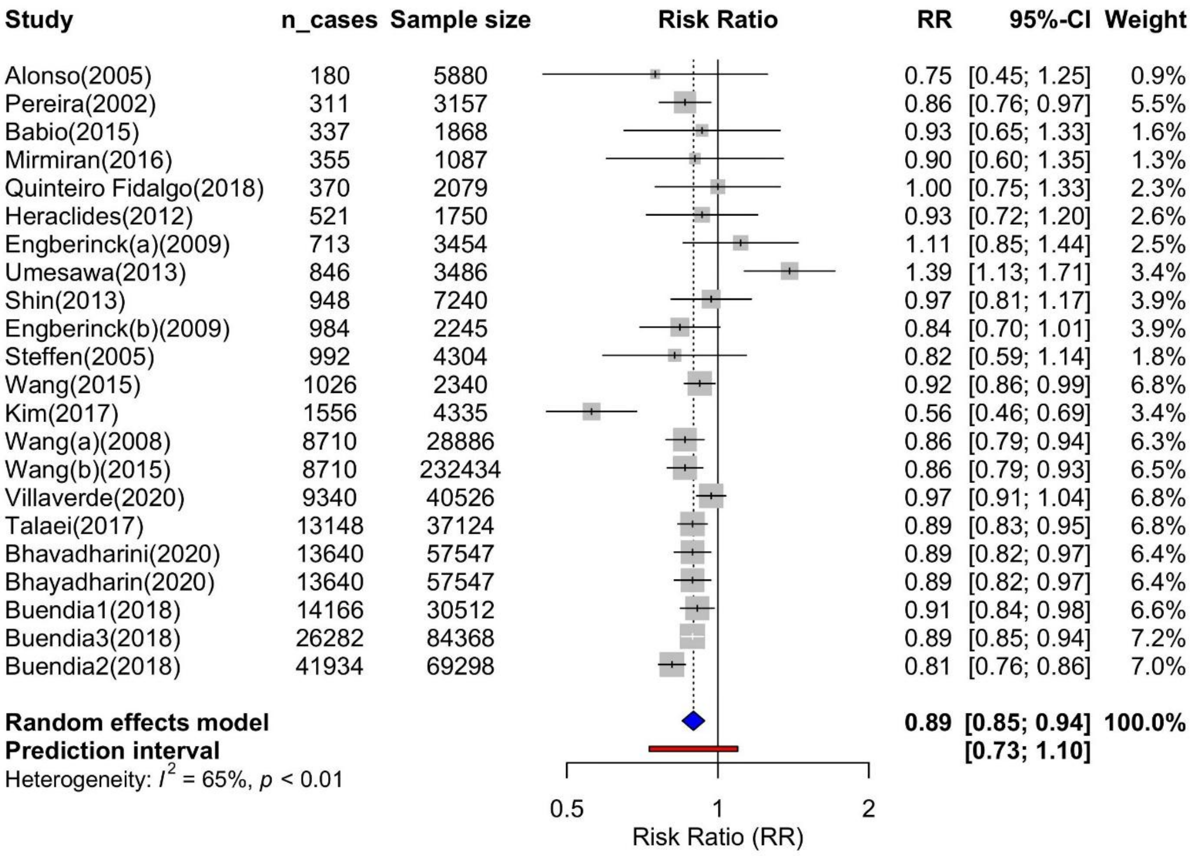

Supplementary Figure S15. Association between total dairy consumption (highest vs. lowest intake level) and hypertension.

Study-specific effect sizes are represented by squares, with the size of each square proportional to the study's weight in the overall meta-analysis. Horizontal lines indicate 95% confidence intervals (CIs). Diamonds represent the pooled RR along with its 95% CI.

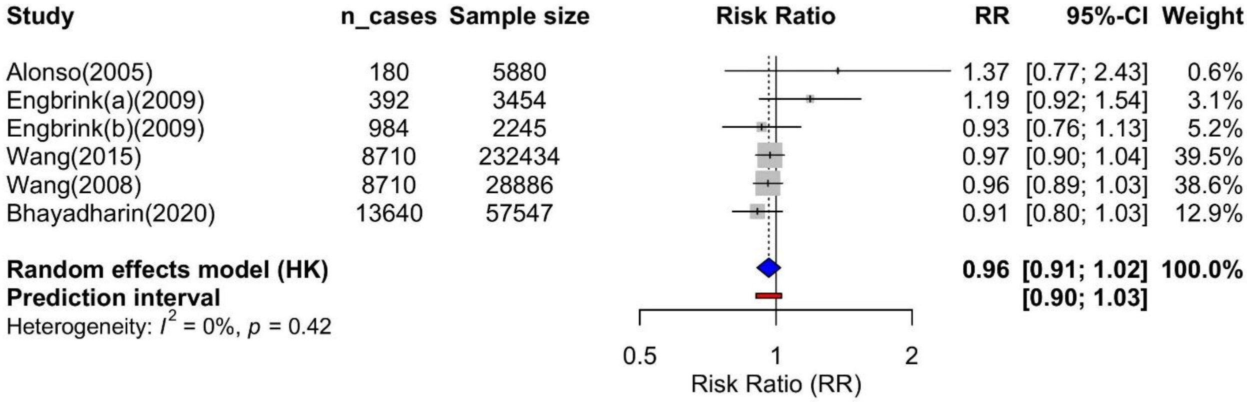

Supplementary Figure S16. Association between high-fat dairy consumption (highest vs. lowest intake level) and hypertension.

Study-specific effect sizes are represented by squares, with the size of each square proportional to the study's weight in the overall meta-analysis. Horizontal lines indicate 95% confidence intervals (CIs). Diamonds represent the pooled RR along with its 95% CI.

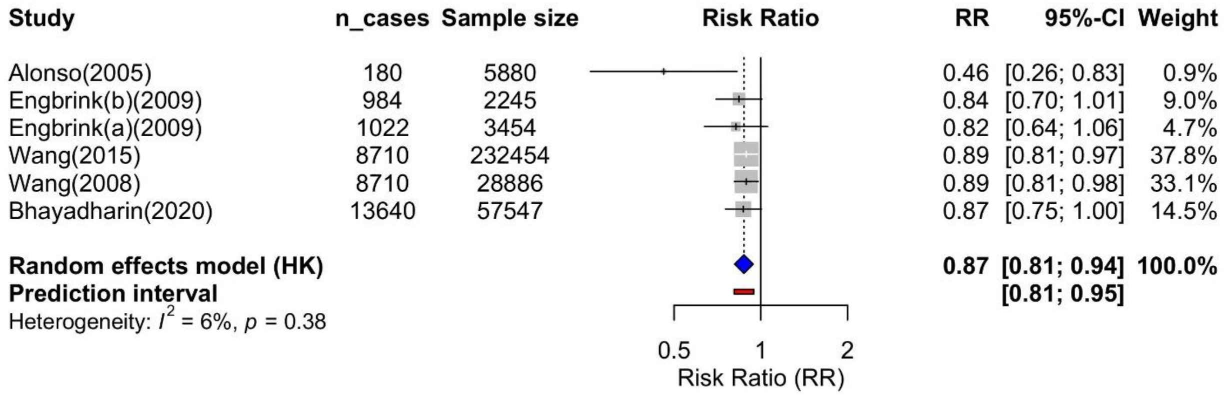

Supplementary Figure S17. Association between low-fat dairy consumption (highest vs. lowest intake level) and hypertension.

Study-specific effect sizes are represented by squares, with the size of each square proportional to the study's weight in the overall meta-analysis. Horizontal lines indicate 95% confidence intervals (CIs). Diamonds represent the pooled RR along with its 95% CI.

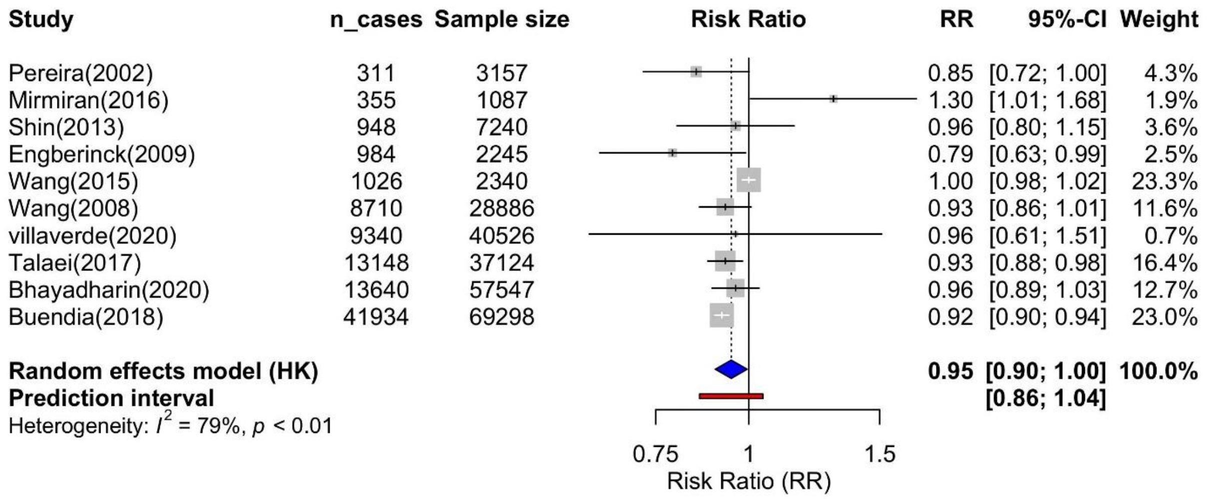

Supplementary Figure S18. Association between milk consumption (highest vs. lowest intake level) and hypertension.

Study-specific effect sizes are represented by squares, with the size of each square proportional to the study's weight in the overall meta-analysis. Horizontal lines indicate 95% confidence intervals (CIs). Diamonds represent the pooled RR along with its 95% CI.

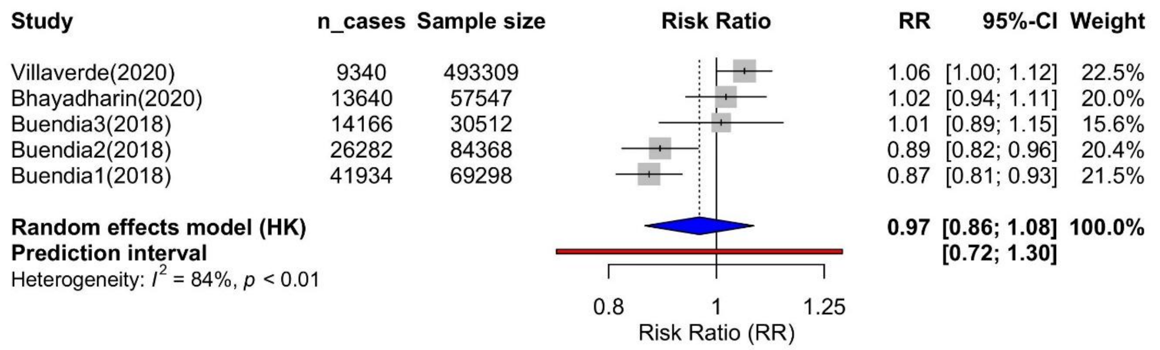

Supplementary Figure S19. Association between yogurt consumption (highest vs. lowest intake level) and hypertension.

Study-specific effect sizes are represented by squares, with the size of each square proportional to the study's weight in the overall meta-analysis. Horizontal lines indicate 95% confidence intervals (CIs). Diamonds represent the pooled RR along with its 95% CI.

References

1. Alexander, D.D.; Bylsma, L.C.; Vargas, A.J.; Cohen, S.S.; Doucette, A.; Mohamed, M.; Irvin, S.R.; Miller, P.E.; Watson, H.; Fryzek, J.P. Dairy consumption and CVD: a systematic review and meta-analysis. *British Journal of Nutrition* **2016**, *115*, 737-750.
2. Bechthold, A.; Boeing, H.; Schwedhelm, C.; Hoffmann, G.; Knüppel, S.; Iqbal, K.; De Henauw, S.; Michels, N.; Devleesschauwer, B.; Schlesinger, S. Food groups and risk of coronary heart disease, stroke and heart failure: a systematic review and dose-response meta-analysis of prospective studies. *Critical reviews in food science and nutrition* **2019**, *59*, 1071-1090.
3. Bhandari, B.; Liu, Z.; Lin, S.; Macniven, R.; Akombi-Inyang, B.; Hall, J.; Feng, X.; Schutte, A.E.; Xu, X. Long-term consumption of 10 food groups and cardiovascular mortality: a systematic review and dose response meta-analysis of prospective cohort studies. *Advances in Nutrition* **2023**, *14*, 55-63.
4. Chen, G.-C.; Wang, Y.; Tong, X.; Szeto, I.M.; Smit, G.; Li, Z.-N.; Qin, L.-Q. Cheese consumption and risk of cardiovascular disease: a meta-analysis of prospective studies. *European journal of nutrition* **2017**, *56*, 2565-2575.
5. Chen, Z.; Ahmed, M.; Ha, V.; Jefferson, K.; Malik, V.; Ribeiro, P.A.; Zuchinali, P.; Drouin-Chartier, J.-P. Dairy product consumption and cardiovascular health: a systematic review and meta-analysis of prospective cohort studies. *Advances in Nutrition* **2022**, *13*, 439-454.
6. Feng, Y.; Zhao, Y.; Liu, J.; Huang, Z.; Yang, X.; Qin, P.; Chen, C.; Luo, X.; Li, Y.; Wu, Y. Consumption of dairy products and the risk of overweight or obesity, hypertension, and type 2 diabetes mellitus: a dose–response meta-analysis and systematic review of cohort studies. *Advances in Nutrition* **2022**, *13*, 2165-2179.
7. Gao, X.; Jia, H.-y.; Chen, G.-c.; Li, C.-y.; Hao, M. Yogurt intake reduces all-cause and cardiovascular disease mortality: a meta-analysis of eight prospective cohort studies. *Chinese journal of integrative medicine* **2020**, *26*, 462-468.
8. Gholami, F.; Khoramdad, M.; Esmailnasab, N.; Moradi, G.; Nouri, B.; Safiri, S.; Alimohamadi, Y. The effect of dairy consumption on the prevention of cardiovascular diseases: A meta-analysis of prospective studies. *Journal of cardiovascular and thoracic research* **2017**, *9*, 1.
9. Guo, J.; Astrup, A.; Lovegrove, J.A.; Gijsbers, L.; Givens, D.I.; Soedamah-Muthu, S.S. Milk and dairy consumption and risk of cardiovascular diseases and all-cause mortality: dose–response meta-analysis of prospective cohort studies. **2017**, *32*, 269-287.
10. Heidari, Z.; Fard, N.R.P.; Clark, C.C.; Haghighatdoost, F. Dairy products consumption and the risk of hypertension in adults: An updated systematic review and dose–response meta-analysis of prospective cohort studies. *Nutrition, Metabolism and Cardiovascular Diseases* **2021**, *31*, 1962-1975.
11. Jakobsen, M.U.; Trolle, E.; Outzen, M.; Mejborn, H.; Grønberg, M.G.; Lyndgaard, C.B.; Stockmarr, A.; Venø, S.K.; Bysted, A. Intake of dairy products and associations with major atherosclerotic cardiovascular diseases: a systematic review and meta-analysis of cohort studies. *Scientific reports* **2021**, *11*, 1303.

12. Kazemi, A.; Soltani, S.; Mokhtari, Z.; Khan, T.; Golzarand, M.; Hosseini, E.; Jayedi, A.; Ebrahimpour-Koujan, S.; Akhlaghi, M. The relationship between major food sources of fructose and cardiovascular disease, cancer, and all-cause mortality: a systematic review and dose-response meta-analysis of cohort studies. *Critical Reviews in Food Science and Nutrition* **2023**, *63*, 4274-4287.
13. Mazidi, M.; Mikhailidis, D.P.; Sattar, N.; Howard, G.; Graham, I.; Banach, M.; Lipid; Collaboration, B.P.M.-a. Consumption of dairy product and its association with total and cause specific mortality–A population-based cohort study and meta-analysis. *Clinical Nutrition* **2019**, *38*, 2833-2845.
14. Mishali, M.; Prizant-Passal, S.; Avrech, T.; Shoenfeld, Y. Association between dairy intake and the risk of contracting type 2 diabetes and cardiovascular diseases: a systematic review and meta-analysis with subgroup analysis of men versus women. *Nutrition reviews* **2019**, *77*, 417-429.
15. Mullie, P.; Pizot, C.; Autier, P. Daily milk consumption and all-cause mortality, coronary heart disease and stroke: a systematic review and meta-analysis of observational cohort studies. *BMC Public Health* **2016**, *16*, 1-8.
16. Naghshi, S.; Sadeghi, O.; Larijani, B.; Esmaillzadeh, A. High vs. low-fat dairy and milk differently affects the risk of all-cause, CVD, and cancer death: A systematic review and dose-response meta-analysis of prospective cohort studies. *Critical reviews in food science and nutrition* **2022**, *62*, 3598-3612.
17. Pimpin, L.; Wu, J.H.; Haskelberg, H.; Del Gobbo, L.; Mozaffarian, D. Is butter back? A systematic review and meta-analysis of butter consumption and risk of cardiovascular disease, diabetes, and total mortality. *PLoS one* **2016**, *11*, e0158118.
18. Qin, L.-Q.; Xu, J.-Y.; Han, S.-F.; Zhang, Z.-L.; Zhao, Y.-Y.; Szeto, I.M. Dairy consumption and risk of cardiovascular disease: an updated meta-analysis of prospective cohort studies. *Asia Pacific journal of clinical nutrition* **2015**, *24*, 90-100.
19. Ralston, R.; Lee, J.; Truby, H.; Palermo, C.; Walker, K. A systematic review and meta-analysis of elevated blood pressure and consumption of dairy foods. *Journal of human hypertension* **2012**, *26*, 3-13.
20. Soedamah-Muthu, S.S.; Ding, E.L.; Al-Delaimy, W.K.; Hu, F.B.; Engberink, M.F.; Willett, W.C.; Geleijnse, J.M. Milk and dairy consumption and incidence of cardiovascular diseases and all-cause mortality: dose-response meta-analysis of prospective cohort studies. *The American journal of clinical nutrition* **2011**, *93*, 158-171.
21. Soedamah-Muthu, S.S.; De Goede, J. Dairy consumption and cardiometabolic diseases: systematic review and updated meta-analyses of prospective cohort studies. *Current nutrition reports* **2018**, *7*, 171-182.
22. Sun, T.; Zhang, Y.; Ding, L.; Zhang, Y.; Li, T.; Li, Q. The relationship between major food sources of fructose and cardiovascular outcomes: a systematic review and dose-response meta-analysis of prospective studies. *Advances in Nutrition* **2023**, *14*, 256-269.
23. Trieu, K.; Bhat, S.; Dai, Z.; Leander, K.; Gigante, B.; Qian, F.; Korat, A.V.A.; Sun, Q.; Pan, X.-F.; Laguzzi, F. Biomarkers of dairy fat intake, incident cardiovascular disease,

and all-cause mortality: A cohort study, systematic review, and meta-analysis. *PLoS medicine* **2021**, *18*, e1003763.
24. Tutunchi, H.; Naghshi, S.; Naemi, M.; Naeini, F.; Esmaillzadeh, A. Yogurt consumption and risk of mortality from all causes, CVD and cancer: a comprehensive systematic review and dose–response meta-analysis of cohort studies. *Public Health Nutrition* **2023**, *26*, 1196-1209.
25. Wu, L.; Sun, D. Consumption of yogurt and the incident risk of cardiovascular disease: a meta-analysis of nine cohort studies. *Nutrients* **2017**, *9*, 315.
26. Zhang, K.; Chen, X.; Zhang, L.; Deng, Z. Fermented dairy foods intake and risk of cardiovascular diseases: a meta-analysis of cohort studies. *Critical reviews in food science and nutrition* **2020**, *60*, 1189-1194.
27. Goncerz, G.; Kojm, P.; Skocelas, S.; Więckowski, K.; Gallina, T.; Pietrzyk, P.; Goncerz, S. Higher milk consumption is not associated with fracture risk reduction: systematic review and meta-analysis. *Folia Medica Cracoviensia* **2022**, 137-153-137-153.
28. Hidayat, K.; Du, X.; Shi, B.-M.; Qin, L.-Q. Systematic review and meta-analysis of the association between dairy consumption and the risk of hip fracture: critical interpretation of the currently available evidence. *Osteoporosis International* **2020**, *31*, 1411-1425.
29. Hidayat, K.; Chen, J.-S.; Wang, T.-C.; Liu, Y.-J.; Shi, Y.-J.; Su, H.-W.; Liu, B.; Qin, L.-Q. The effects of milk supplementation on bone health indices in adults: a meta-analysis of randomized controlled trials. *Advances in Nutrition* **2022**, *13*, 1186-1199.
30. Ma, D.F.; Zheng, W.; Ding, M.; Zhang, Y.M.; Wang, P.Y. Milk intake increases bone mineral content through inhibiting bone resorption: Meta-analysis of randomized controlled trials. *e-SPEN Journal* **2013**, *8*, e1-e7, doi:https://doi.org/10.1016/j.clnme.2012.10.005.
31. Malmir, H.; Larijani, B.; Esmaillzadeh, A. Consumption of milk and dairy products and risk of osteoporosis and hip fracture: a systematic review and Meta-analysis. *Critical reviews in food science and nutrition* **2020**, *60*, 1722-1737.
32. Matía-Martín, P.; Torrego-Ellacuría, M.; Larrad-Sainz, A.; Fernández-Pérez, C.; Cuesta-Triana, F.; Rubio-Herrera, M.Á. Effects of milk and dairy products on the prevention of osteoporosis and osteoporotic fractures in Europeans and non-Hispanic Whites from North America: a systematic review and updated meta-analysis. *Advances in Nutrition* **2019**, *10*, S120-S143.
33. Shi, Y.; Zhan, Y.; Chen, Y.; Jiang, Y. Effects of dairy products on bone mineral density in healthy postmenopausal women: a systematic review and meta-analysis of randomized controlled trials. *Archives of Osteoporosis* **2020**, *15*, 1-8.
34. Dukuzimana, J.; Janzi, S.; Habberstad, C.; Zhang, S.; Borné, Y.; Sonestedt, E. High consumption of dairy products and risk of major adverse coronary events and stroke in a Swedish population. *British Journal of Nutrition* **2024**, *131*, 500-511.
35. Guo, J.; Givens, D.I.; Heitmann, B.L. Association between dairy consumption and cardiovascular disease events, bone fracture and all-cause mortality. *PloS one* **2022**, *17*, e0271168.
36. Koskinen, T.T.; Virtanen, H.E.; Voutilainen, S.; Tuomainen, T.-P.; Mursu, J.; Virtanen, J.K. Intake of fermented and non-fermented dairy products and risk of incident CHD:

the Kuopio Ischaemic Heart Disease Risk Factor Study. *British Journal of Nutrition* **2018**, *120*, 1288-1297.
37. Olsson, E.; Larsson, S.C.; Höijer, J.; Kilander, L.; Byberg, L. Milk and fermented milk consumption and risk of stroke: longitudinal study. *Nutrients* **2022**, *14*, 1070.
38. Sellem, L.; Srour, B.; Jackson, K.G.; Hercberg, S.; Galan, P.; Kesse-Guyot, E.; Julia, C.; Fezeu, L.; Deschasaux-Tanguy, M.; Lovegrove, J.A. Consumption of dairy products and CVD risk: results from the French prospective cohort NutriNet-Santé. *British Journal of Nutrition* **2022**, *127*, 752-762.
39. Talaei, M.; Koh, W.-P.; Yuan, J.-M.; Pan, A. The association between dairy product intake and cardiovascular disease mortality in Chinese adults. *European Journal of Nutrition* **2017**, *56*, 2343-2352.
40. Um, C.Y.; Prizment, A.; Hong, C.-P.; Lazovich, D.; Bostick, R.M. Associations of calcium and dairy product intakes with all-cause, all-cancer, colorectal cancer and CHD mortality among older women in the Iowa Women's Health Study. *British Journal of Nutrition* **2019**, *121*, 1188-1200.
41. Wang, X.J.; Jiang, C.Q.; Zhang, W.S.; Zhu, F.; Jin, Y.L.; Woo, J.; Cheng, K.K.; Lam, T.H.; Xu, L. Milk consumption and risk of mortality from all-cause, cardiovascular disease and cancer in older people. *Clinical Nutrition* **2020**, *39*, 3442-3451.
42. Zhou, J.; Wu, Z.; Lin, Z.; Wang, W.; Wan, R.; Liu, T. Association of milk consumption with all-cause mortality and cardiovascular outcomes: a UK Biobank based large population cohort study. *Journal of Translational Medicine* **2023**, *21*, 130.
43. Benatar, J.R.; Sidhu, K.; Stewart, R.A. Effects of high and low fat dairy food on cardio-metabolic risk factors: a meta-analysis of randomized studies. *PloS one* **2013**, *8*, e76480.
44. Companys, J.; Pla-Pagà, L.; Calderón-Pérez, L.; Llauradó, E.; Solà, R.; Pedret, A.; Valls, R.M. Fermented dairy products, probiotic supplementation, and cardiometabolic diseases: a systematic review and meta-analysis. *Advances in nutrition* **2020**, *11*, 834-863.
45. Dixon, A.; Robertson, K.; Yung, A.; Que, M.; Randall, H.; Wellalagodage, D.; Cox, T.; Robertson, D.; Chi, C.; Sun, J. Efficacy of probiotics in patients of cardiovascular disease risk: a systematic review and meta-analysis. *Current hypertension reports* **2020**, *22*, 1-27.
46. Dong, J.-Y.; Szeto, I.M.; Makinen, K.; Gao, Q.; Wang, J.; Qin, L.-Q.; Zhao, Y. Effect of probiotic fermented milk on blood pressure: a meta-analysis of randomised controlled trials. *British Journal of Nutrition* **2013**, *110*, 1188-1194.
47. Fabiani, R.; Naldini, G.; Chiavarini, M. Dietary patterns in relation to low bone mineral density and fracture risk: a systematic review and meta-analysis. *Advances in Nutrition* **2019**, *10*, 219-236.
48. Fontecha, J.; Calvo, M.V.; Juarez, M.; Gil, A.; Martínez-Vizcaino, V. Milk and dairy product consumption and cardiovascular diseases: an overview of systematic reviews and meta-analyses. *Advances in nutrition* **2019**, *10*, S164-S189.
49. Hidayat, K.; Du, H.-Z.; Yang, J.; Chen, G.-C.; Zhang, Z.; Li, Z.-N.; Qin, L.-Q. Effects of milk proteins on blood pressure: a meta-analysis of randomized control trials. *Hypertension Research* **2017**, *40*, 264-270.

50. Kanis, J.A.; Johansson, H.; Oden, A.; De Laet, C.; Johnell, O.; Eisman, J.A.; McCloskey, E.; Mellstrom, D.; Pols, H.; Reeve, J. A meta-analysis of milk intake and fracture risk: low utility for case finding. *Osteoporosis international* **2005**, *16*, 799-804.
51. Larsson, S.C.; Crippa, A.; Orsini, N.; Wolk, A.; Michaëlsson, K. Milk consumption and mortality from all causes, cardiovascular disease, and cancer: a systematic review and meta-analysis. *Nutrients* **2015**, *7*, 7749-7763.
52. Liang, J.; Zhou, Q.; Kwame Amakye, W.; Su, Y.; Zhang, Z. Biomarkers of dairy fat intake and risk of cardiovascular disease: A systematic review and meta analysis of prospective studies. *Critical reviews in food science and nutrition* **2018**, *58*, 1122-1130.
53. Micha, R.; Shulkin, M.L.; Penalvo, J.L.; Khatibzadeh, S.; Singh, G.M.; Rao, M.; Fahimi, S.; Powles, J.; Mozaffarian, D. Etiologic effects and optimal intakes of foods and nutrients for risk of cardiovascular diseases and diabetes: systematic reviews and meta-analyses from the Nutrition and Chronic Diseases Expert Group (NutriCoDE). *PloS one* **2017**, *12*, e0175149.
54. Mishali, M.; Kisner, M.; Avrech, T. Funding sources and outcomes of dairy consumption research–a meta-analysis of cohort studies: the case of type-2 diabetes and cardiovascular diseases. *International Dairy Journal* **2019**, *95*, 65-70.
55. Ong, A.M.; Kang, K.; Weiler, H.A.; Morin, S.N. Fermented milk products and bone health in postmenopausal women: a systematic review of randomized controlled trials, prospective cohorts, and case-control studies. *Advances in nutrition* **2020**, *11*, 251-265.
56. Schwingshackl, L.; Schwedhelm, C.; Hoffmann, G.; Knüppel, S.; Iqbal, K.; Andriolo, V.; Bechthold, A.; Schlesinger, S.; Boeing, H. Food groups and risk of hypertension: a systematic review and dose-response meta-analysis of prospective studies. *Advances in nutrition* **2017**, *8*, 793-803.
57. Soedamah-Muthu, S.S.; Verberne, L.D.; Ding, E.L.; Engberink, M.F.; Geleijnse, J.M. Dairy consumption and incidence of hypertension: a dose-response meta-analysis of prospective cohort studies. *Hypertension* **2012**, *60*, 1131-1137.
58. Soto-Méndez, M.J.; Rangel-Huerta, O.D.; Ruiz-López, M.D.; de Victoria, E.M.; Anguita-Ruiz, A.; Gil, A. Role of functional fortified dairy products in cardiometabolic health: a systematic review and meta-analyses of randomized clinical trials. *Advances in Nutrition* **2019**, *10*, S251-S271.
59. Sun, J.; Buys, N. Effects of probiotics consumption on lowering lipids and CVD risk factors: a systematic review and meta-analysis of randomized controlled trials. *Annals of medicine* **2015**, *47*, 430-440.
60. Xu, J.-Y.; Qin, L.-Q.; Wang, P.-Y.; Li, W.; Chang, C. Effect of milk tripeptides on blood pressure: a meta-analysis of randomized controlled trials. *Nutrition* **2008**, *24*, 933-940.
